# Consumption of breast milk, formula and other non-human milk by children aged under 2 years: analysis of eighty-six low- and middle-income countries

**DOI:** 10.1017/S1368980020004061

**Published:** 2022-03

**Authors:** Paulo AR Neves, Aluísio JD Barros, Phillip Baker, Ellen Piwoz, Thiago M Santos, Giovanna Gatica-Domínguez, Juliana S Vaz, Nigel Rollins, Cesar G Victora

**Affiliations:** 1 International Center for Equity in Health, Federal University of Pelotas, Rua Marechal Deodoro, 1160, 3rd floor, Pelotas 96020-220, Brazil; 2 Institute for Physical Activity and Nutrition, Deakin University, Melbourne, Australia; 3 Global Development Program, The Bill & Melinda Gates Foundation, Seattle, WA, USA; 4 Department of Maternal, Newborn, Child and Adolescent Health, World Health Organization, Geneva, Switzerland

**Keywords:** Breast-feeding, Infant formula, Breast milk substitutes, Socio-economic factors, Economic status, Developing countries, Nutrition surveys

## Abstract

**Objective:**

To investigate the prevalence and socio-economic inequalities in breast milk, breast milk substitutes (BMS) and other non-human milk consumption, by children under 2 years in low- and middle-income countries (LMIC).

**Design:**

We analysed the prevalence of continued breast-feeding at 1 and 2 years and frequency of formula and other non-human milk consumption by age in months. Indicators were estimated through 24-h dietary recall. Absolute and relative wealth indicators were used to describe within- and between-country socio-economic inequalities.

**Setting:**

Nationally representative surveys from 2010 onwards from eighty-six LMIC.

**Participants:**

394 977 children aged under 2 years.

**Results:**

Breast-feeding declined sharply as children became older in all LMIC, especially in upper-middle-income countries. BMS consumption peaked at 6 months of age in low/lower-middle-income countries and at around 12 months in upper-middle-income countries. Irrespective of country, BMS consumption was higher in children from wealthier families, and breast-feeding in children from poorer families. Multilevel linear regression analysis showed that BMS consumption was positively associated with absolute income, and breast-feeding negatively associated. Findings for other non-human milk consumption were less straightforward. Unmeasured factors at country level explained a substantial proportion of overall variability in BMS consumption and breast-feeding.

**Conclusions:**

Breast-feeding falls sharply as children become older, especially in wealthier families in upper-middle-income countries; this same group also consumes more BMS at any age. Country-level factors play an important role in explaining BMS consumption by all family wealth groups, suggesting that BMS marketing at national level might be partly responsible for the observed differences.

Breast-feeding is essential for children, their mothers and societies as it is associated with reduced risk of short- and long-term harmful health outcomes in all country contexts^(1,2)^. Breast-feeding also associates with increases in intelligence and educational attainment^(3)^, hence contributing to workforce productivity and sustainable development^(2,3)^. Despite the recommendation by the WHO for all children to be breastfed to 24 months or longer, the prevalence of continued breast-feeding at 1 year remains below 60 % in upper-middle-income countries and under 30 % in high-income countries^(1)^, being inversely correlated with national wealth^(1,4)^.

The types of milk consumed by infant and young children include three broad categories: breast milk, commercial breast milk substitutes (BMS - formula and BMS are used interchangeably) or other types of non-human milk. One of the major barriers to achieving optimal breast-feeding practices in all countries is the aggressive marketing of BMS^(2,4,5)^. Recent studies show an escalation in BMS sales worldwide, mostly concentrated in high- and upper-middle-income countries^(6,7)^. This not only includes standard (for infants aged 0–6 months) and follow-up (6–12 months) formulas but also toddler (13–36 months) category. This is despite the WHO having long considered follow-up and toddler milks as unnecessary and unsuitable substitutes for breast milk^(8)^. Recent evidence from the USA shows that toddler milk sales grew by 158 % between 2006 and 2015, whereas infant formula sales decreased by 7 %^(7)^. Similar trends have been reported for emerging economies with rapid increases in per capita toddler milk sales volumes, especially in China, Brazil, Peru, and Turkey^(6)^.

Between-country inequalities in BMS consumption by infants aged under 6 months have been reported in low- and middle-income countries (LMIC), where formula consumption is directly correlated with national gross domestic product^(4)^. In addition, within-country inequalities have been documented with wide gaps between rich and poor households in some regions of the world, including Latin America & the Caribbean and in East Asia & the Pacific. Additionally, infant formula consumption was inversely correlated with national-level breast-feeding prevalence at 1 year^(4)^. In addition, breast-feeding was more common among children from poorer than richer households^(4)^.

As per capita, volume sales of all BMS categories are rapidly escalating in highly populated LMIC – and particularly in upper-middle-income countries^(6)^ – it is important to document the prevalence of BMS consumption. However, no studies to date have reported on BMS consumption among children aged 6–23 months using household survey data, nor on socio-economic inequalities in BMS consumption. Therefore, we aimed to investigate the prevalence and socio-economic inequalities in the consumption of breast milk, BMS and other non-human milks by children under 2 years in LMIC, in order to help guide policymaking and programming in these countries.

We explore the relationship between the consumption of different types of milk with relative wealth and absolute income levels. We hypothesised that, as national wealth increases, BMS consumption increases and breast-feeding decreases. We also postulated that, formula being an expensive product for most LMIC families, its consumption would be directly associated with a certain absolute level of income.

## Methods

We sourced data from nationally representative cross-sectional surveys conducted periodically in LMIC, namely Demographic Health Surveys^(9)^ and Multiple Indicator Cluster Surveys^(10)^. These surveys cover a large number of reproductive, maternal, newborn and child health indicators, employing multistage sampling strategies to collect household-level data. We selected the most recent publicly available survey for each country carried out since 2010. In both types of surveys, face-to-face interviews were performed through standardised questionnaires, so that both types of surveys are highly comparable^(11)^. Women of childbearing age (15–49 years) were interviewed by trained fieldworkers, who collected data on infant and young child feeding (IYCF) practices using 24-h recall for the youngest child born in the last 2 years before the survey^(12,13)^. We also included the nationally representative surveys conducted in Ecuador and Peru, after harmonisation of its data set and variables in accordance with the Demographic Health Surveys/Multiple Indicator Cluster Surveys standards^(14,15)^.

Data for more than 100 surveys were available at the time of analysis. However, some surveys had to be excluded, as detailed in Supplemental Fig. 1. Therefore, our analysis is based on data for eighty-six LMIC.

We calculated the following feeding indicators: continued breast-feeding at 1 and 2 years (proportion of children between 12–15 and 20–23 months of age, respectively, who were breastfed)^(12,13)^; formula consumption under 6 and between 6 and 23 months (proportion of children between 0–5 months and 6–23 months, respectively, who were fed formula) and other non-human milk consumption under 6 and between 6 and 23 months (proportion of children between 0–5 months and 6–23 months, respectively, who were fed non-human milk, other than formula, e.g. cow and goat milk). The consistency and quality of the calculated estimates were checked by comparison with the published figures in the Demographic Health Surveys/Multiple Indicator Cluster Surveys reports for continued breast-feeding at 1 and 2 years^(12,13)^; all differences were <1 percentage point. In our analyses, we use the terms formula and BMS as synonyms.

Countries were grouped according to World Bank income classification at the year of survey implementation^(16,17)^. Supplemental Table 1 provides a list of countries included in the analyses, their classification and the sample size by age range. All regional and income estimates were weighted by the population size of children in each age range in the country retrieved from the World Bank Population Estimates and Projections, in the year the survey was carried out^(18)^.

Within-country relative socio-economic inequalities were assessed using household wealth indices that are included in the survey data sets. These are based on household assets (television, refrigerator, car, etc.) and building characteristics (wood floor, brick wall and roof). The index was derived using principal component analysis^(19)^. Two separate analyses were carried for urban and rural households, later combined into a single score using a scaling procedure to allow comparability between areas of residence. The definition of area of residence is country-specific. The resulting index was then split into five groups of equal sample size – the quintiles. The first quintile represents the households with the poorest 20 % of the population, and the fifth quintile the wealthiest 20 % of the sample^(20,21)^.

Furthermore, we attributed absolute income values to each wealth quintile based on Fink *et al.*^(22)^. Briefly, the absolute income is estimated for each wealth quintile based on the national income levels (gross domestic product) retrieved from the World Bank, and the national income inequality data obtained from the Standardized World Income Inequality Database Gini index, to generate parameters of a log-normal distribution^(23,24)^. Considering the household asset index, dollar values are then assigned to each wealth quintile. Absolute income is expressed in 2011 purchasing power parity adjusted international dollars.

We estimated consumption of each type of milk by age in months and then used local polynomial smoothing to graph trajectories of consumption of the three types of milk by age for countries and for the poorest and richest quintiles in each country. We also explored the share of formula consumption among all types of non-human milk (formula plus other non-human milk) consumed by children aged under 2 years (see online supplementary material, Supplemental Table 2).

### Statistical analysis

Multilevel linear regression models were performed to explore the relationship between feeding indicators and absolute income (log-transformed to better fit the models). Countries were deemed the highest hierarchical level, and quintiles within each country as the second hierarchical level. Analysis was adjusted by the World Bank income classification, and interaction terms were fitted. We present beta coefficients (in percentage points) along with 95 % CI for each outcome, as well as the proportion of the overall variance explained by the highest level (country). We graphed scatter plots to illustrate the relation between feeding indicators and absolute income, including predicted lines for each income level group. Fractional polynomials were used to describe non-linear associations in graphical form. In the multilevel analyses, however, using polynomials did not improve the fit of the model compared with a linear equation, and for the sake of simplicity, we adopted the linear regression. Lastly, we plotted within-country inequalities for the top three and bottom three countries in terms of the consumption of formula and breast milk. All analyses were run using Stata 16.0 (Stata Corp.), and the graphs were built up on R (version 3.6.1). National estimates and by wealth quintiles are given for each indicator with the 95 % CI (see online supplementary material, Supplemental Tables 3–8).

## Results

Data were available from eighty-six countries (see online supplementary material, Supplemental Fig. 1), with sample sizes ranging from 635 children aged under 2 years in Kosovo to 94 371 in India. The median year of the surveys was 2014, ranging from 2010 (Bhutan, Burkina Faso, Central African Republic, Colombia and South Sudan) to 2018 (Iraq, Kyrgyzstan, Peru, Suriname and Tunisia). Studied countries represented 41·6 % of upper middle, 70·2 % of lower middle and 90·3 % of low-income countries.

**Fig. 1:**
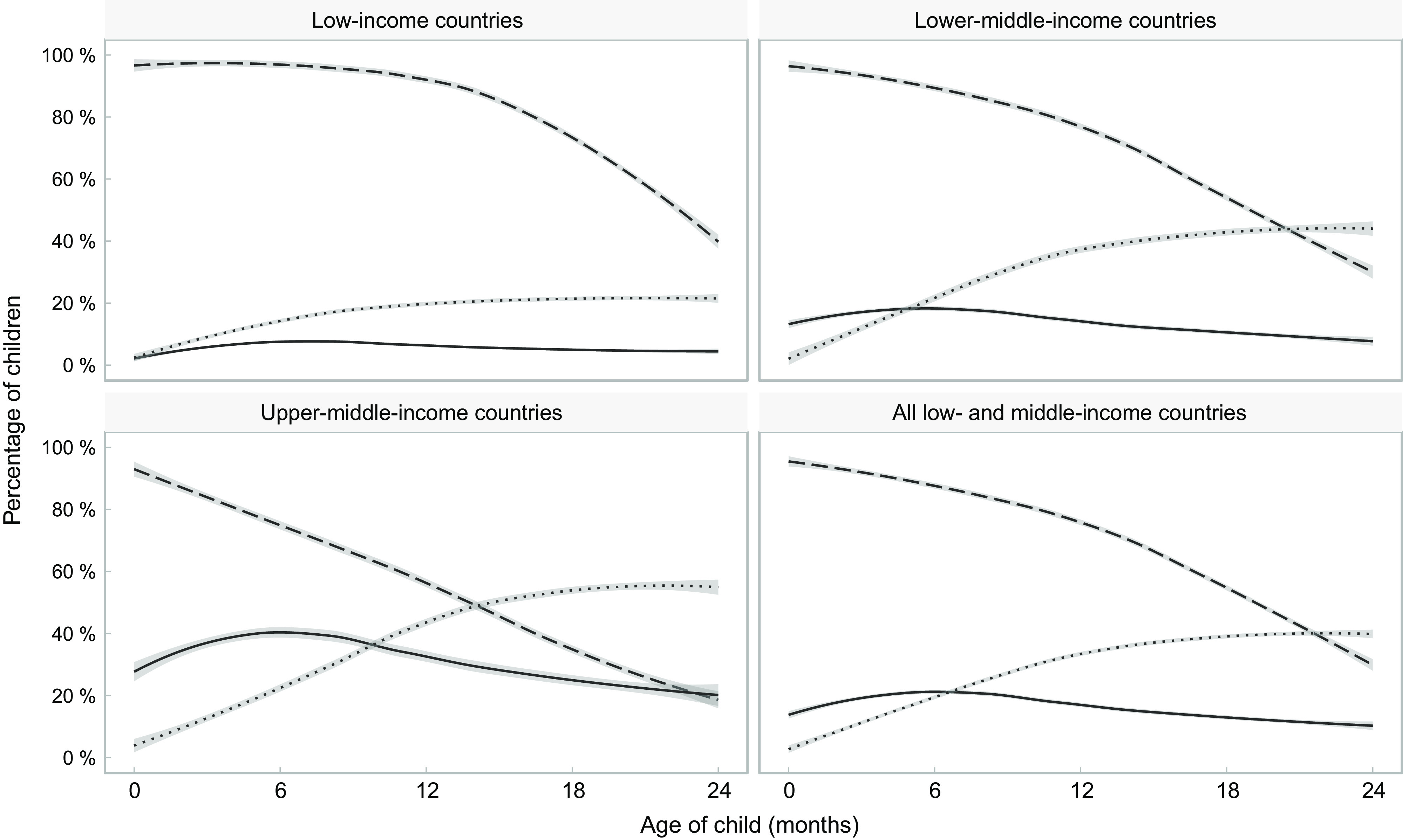
Trajectories of the frequency of consumption of different types of milk by children’s age. Shaded lines represent 95 % CI. 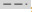, Any breast-feeding; 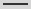, formula consumption; 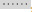, other milk consumption

Figure 1 shows the proportions of children receiving breast milk, formula and other types of milk, by age and country income groups. Breast-feeding prevalence falls with increasing age in all income groups, below the global recommendation for continued breast-feeding beyond 2 years, particularly in upper-middle-income countries. Formula use is most common in middle-income groups. It increases slightly with age in all country income groups, but after 6–9 months, it tends to decline. Other milk consumption increases with age during the first year of life and remains stable thereafter. In low-income countries, formula and other types of milk are used by similar proportions of children at birth, but formula consumption remains stable at very low levels throughout the age range under study, whereas other milks increase in frequency up to around 12 months, remaining stable thereafter. A higher proportion of children consumed formula than other types of milk during the first 5 months in lower-middle-income countries, and during the first 10–11 months in upper-middle-income countries. In all groups of countries, other milks are more frequently used than formula and less than half of children were breastfed at 24 months of age.

**Fig. 2:**
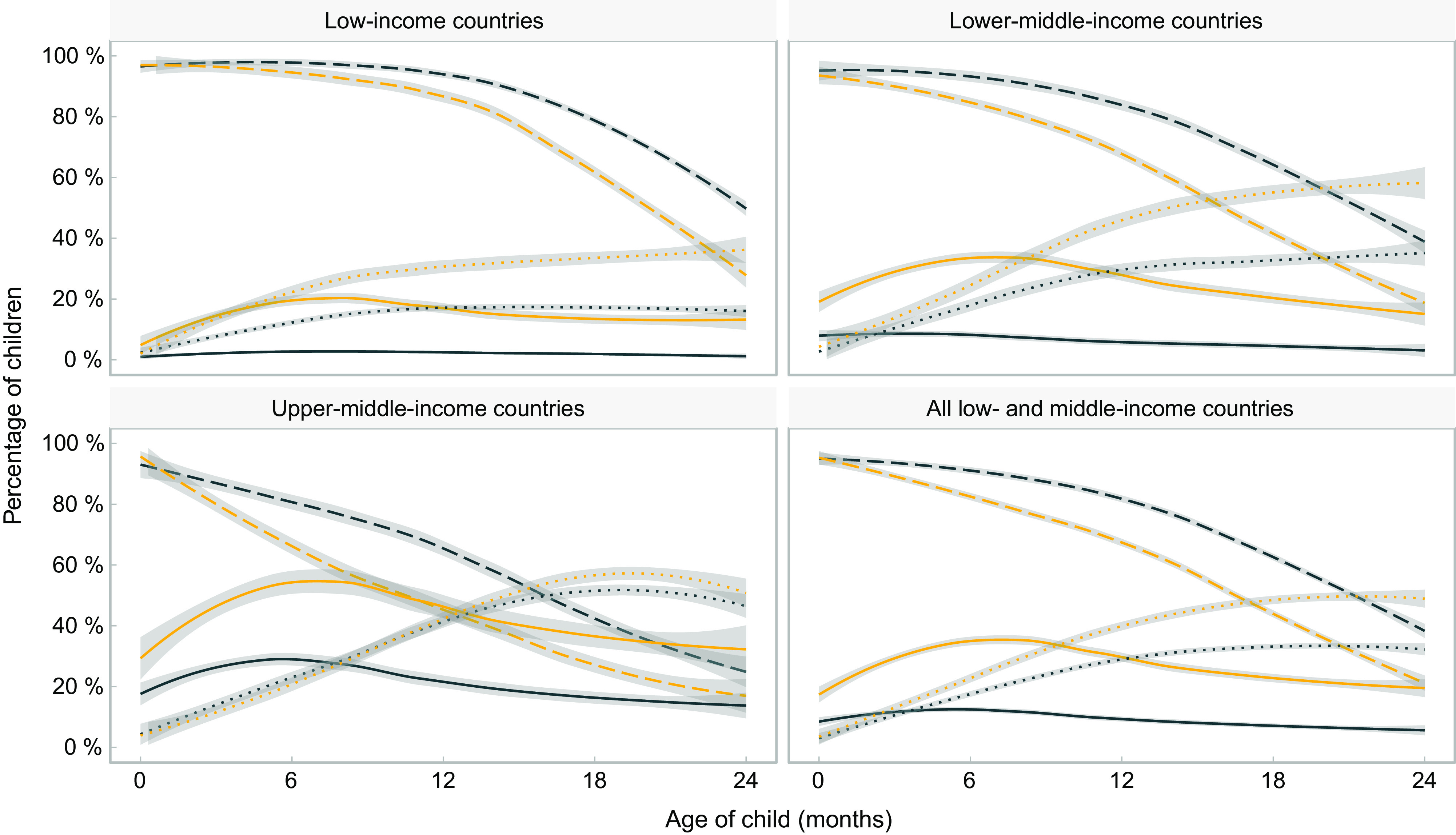
Trajectories of the frequency of consumption of different types of milk by children’s age, by poorest and wealthiest quintiles. Shaded lines represent 95 % CI. 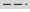, Any breast-feeding – Poorest; 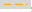, any breast-feeding – Richest; 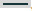, formula consumption – Poorest; 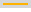, formula consumption – Richest; 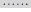, other milk consumption – Poorest; 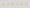, other milk consumption − Richest

Figure 2 expands upon the results in Fig. 1, showing feeding patterns for the wealthiest and poorest quintiles in each group of countries. In all income groups, breast-feeding is less common, while the consumption of formula and non-human milk is more common among children from the richest families, compared with children from the poorest families. The exception occurs in upper-middle-income countries, where consumption of other types of milk is almost equally prevalent for children from different quintiles. The only group where formula becomes more common than breast-feeding is children from the wealthiest quintile in upper-middle-income countries, where this occurs at around 20 months of age.

Based on the fitted curves shown in Figs. 1 and 2, formula consumed by children as a proportion of all types of non-human milk combined was 69 %, 35 %, 26 % and 17 % at 0, 6, 12 and 24 months of age, respectively, in low-income countries; for the same age groups, in lower-middle-income countries, the shares were 94 %, 45 %, 29 % and 16 %, and in upper-middle-income countries 97 %, 63 %, 44 % and 28 %. When the analyses are broken down by wealth quintiles, the results confirm the higher use of formula among children from wealthier families, compared with those in poor families, in all country income groups (see online supplementary material, Supplemental Table 2).

**Fig. 3:**
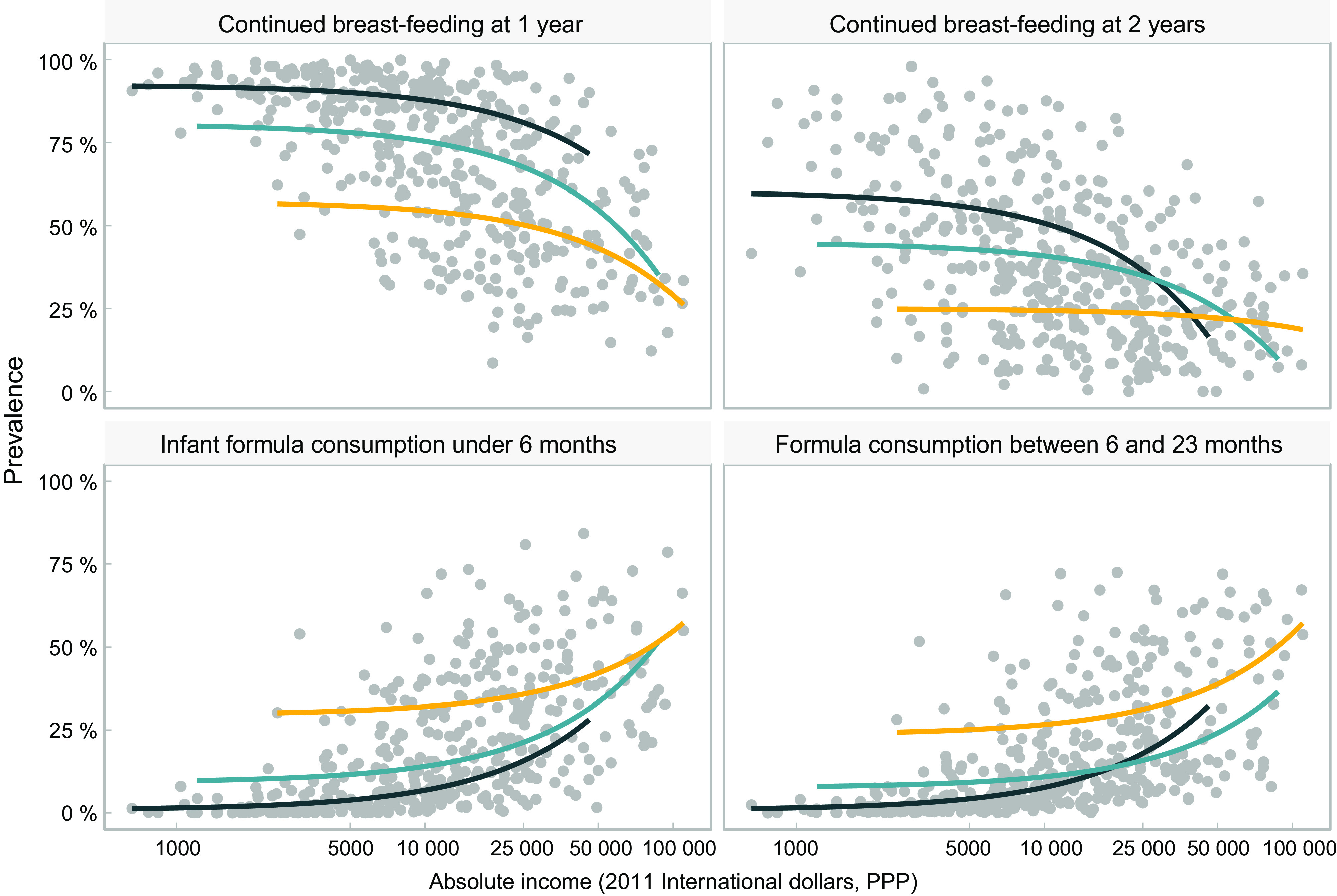
Relationship between feeding practices of children and absolute income (2011 international dollars, purchasing power parity). Each dot represents a wealth quintile in each survey (430 dots). World Bank income group: 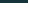, low-income; 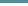, lower middle income; 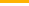, upper middle income

Figure 3 illustrates the inverse, non-linear relationship between breast-feeding at 1 and 2 years and absolute income across all age ranges and income groups, although the pattern is less marked for continued breast-feeding at 2 years in upper-middle-income countries. Findings for formula use are almost a mirror image of those for breast-feeding. In all age ranges, consumption of formula increases with higher absolute income. At a higher absolute income level, use of formula escalates in all groups of countries.

Patterns for other types of milk are less straightforward (see online supplementary material, Supplemental Fig. 2). Among young infants, the association with household income is weak; however, for children aged 6–23 months, the use of other milks becomes more common with increasing household income, particularly in low- and lower-middle-income countries. As observed for formula and breast milk, there are large differences between countries for high or low consumption of other milks, at the same level of absolute income.

**Fig. 4:**
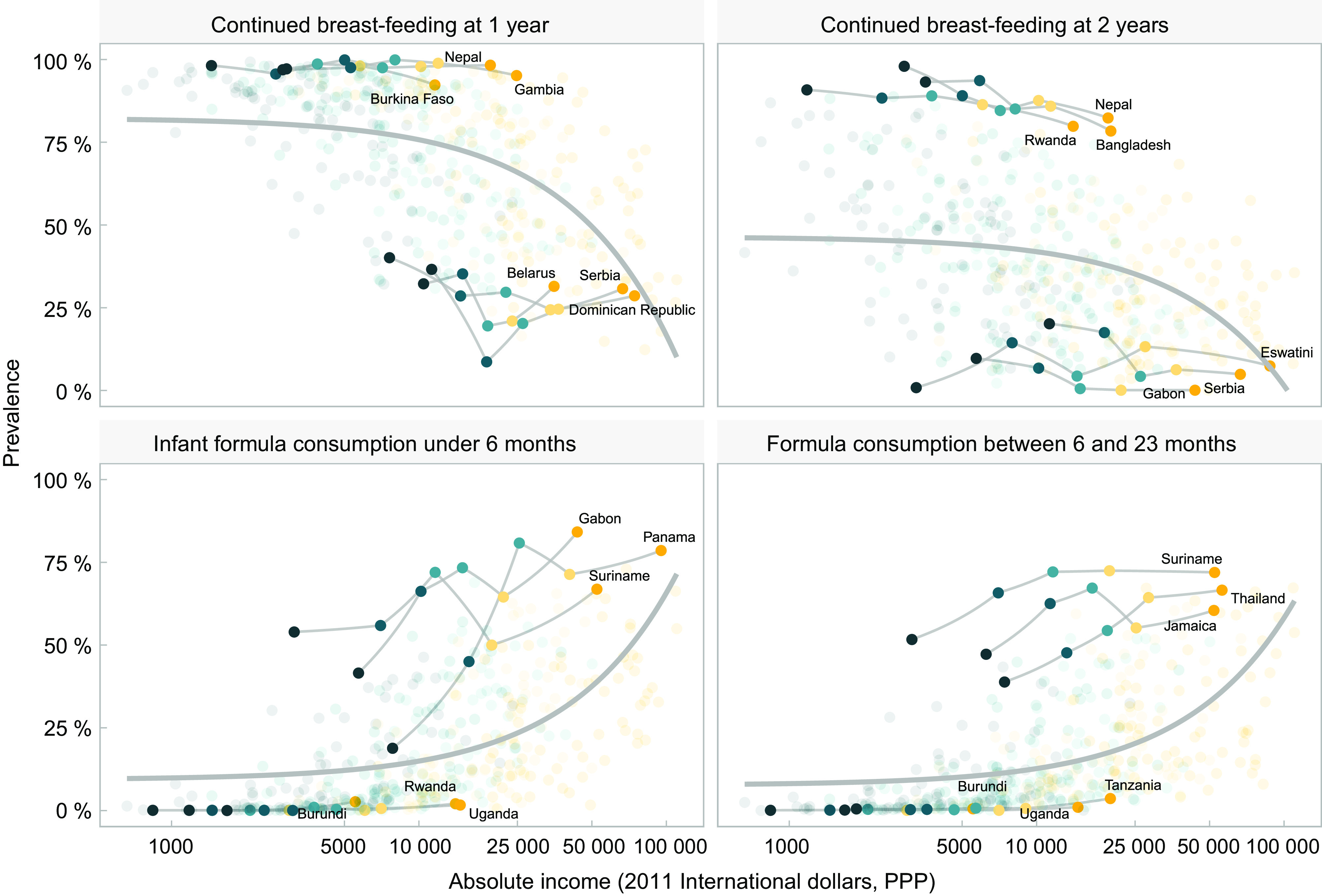
Top and bottom countries in relation to the feeding practices and absolute income (2011 international dollars, purchasing power parity) and relative wealth (represented as shaded dots in the background). Each dot represents a wealth quintile in each survey (430 dots). Wealth quintiles: 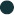, Poorest; 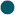, second; 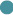, third; 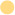, fourth; 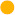, wealthiest

A common finding in the four charts included in Fig. 3 is that for the same level of absolute household income, breast-feeding rates tend to be higher in low-income than in middle-income countries, while the reverse pattern is observed for formula. This suggests that characteristics of the countries other than income alone may be driving feeding patterns. Figure 4 is an expanded version of Fig. 3, showing the top three and bottom three countries according to national prevalence of use of each type of milk. All countries in the top formula consumption groups belong to the upper-middle-income category, whereas those in the low-consumption group are low-income countries. For breast-feeding, all quintiles in the high-consumption group of countries show substantially higher rates, regardless of household income levels. The opposite pattern is observed for formula, where at similar household income levels, there are striking differences between the three top and bottom consumer countries. Even the poorest households in the top consumers countries used more formula than the wealthiest quintiles in bottom consumers countries.

We further explored the heterogeneity among countries using multilevel analyses, with countries representing the first level and log household income per quintile within each country in the second level (Table 1). In this type of analysis, it is common to observe that the higher level variable - countries - explains a small proportion of the overall variance. Yet in this analysis, we found that this level explained nearly 30 % of the variance in infant formula consumption under 6 months and of continued breast-feeding at 1 year, thus suggesting the existence of country characteristics that markedly influence feeding patterns by all groups of wealth, which deserve more in-depth analysis in the future.

**Table 1:** Multilevel association between feeding indicators and log absolute income (2011 international dollars, purchasing power parity), adjusted by World Bank income level

Indicator	Unadjusted *β* *	95 % CI	Adjusted *β* *	95 % CI	Variance (%)†	*P* ‡
Continued breast-feeding at 1 year	−8·0	−9·0, −7·0	−9·0	−10·8, −7·2	27·3	<0·0001
Continued breast-feeding at 2 years	−8·2	−9·3, −7·1	−3·4	−5·3, −1·5	21·2	<0·0001
Infant formula consumption under 6 months	8·1	7·2, 9·0	8·5	6·8, 10·1	29·4	<0·0001
Formula consumption between 6 and 23 months	8·7	8·0, 9·4	10·6	9·4, 11·8	17·6	<0·0001
Other milk consumption under 6 months	1·0	0·4, 1·5	−0·7	−0·1, 0·3	20·0	0·171
Other milk consumption between 6 and 23 months	6·1	5·2, 7·0	2·2	0·7, 3·7	15·4	0·004

* Betas represent changes in each indicator as percentage points by increments of 1 log of absolute income.

† Variance percentage that is explained by the highest hierarchical level in the adjusted analysis: countries.

‡ *P* value.

## Discussion

This is the first multi-country investigation describing the prevalence of BMS and other non-human milk consumption among children aged 6–23 months. We confirm the well-known finding that breast milk consumption decreases with the age of the child, across all country income groups, and that early cessation of breast-feeding is more common among children from wealthier families in all countries^(1,25)^. BMS consumption peaks at around 6–9 months of age in low- and lower-middle-income countries, and somewhat later (up to 12 months) in upper-middle-income countries. About 40 % of all children from wealthier households in upper-middle-income countries were reported as consuming BMS in the second year of life, suggesting that toddler milk consumption is relatively common in this group. At the other extreme, <10 % of children from the poorest households in low-income countries consumed BMS in the first 2 years of life.

Our findings further show that other types of non-human milk are an important food source for children under 2 years in all LMIC, especially in middle-income countries, and that BMS are progressively replaced by these types of milk as children become older. However, this varies markedly by family wealth and country income group. These foods are rarely explored in the IYCF literature with most available studies reporting on associations with health outcomes, such as allergy risk or child growth^(26,27)^. Our findings also show that continued breast-feeding in LMIC declines in all income groups as children become older. The early cessation of exclusive breast-feeding and shorter the duration of continued breast-feeding have implications for growth and development of children, as this increases the likelihood of exposure to unhealthy foods, sub-optimal dietary diversity and lower consumption of nutrient-rich foods^(28)^.

These results add further evidence that follow-on and toddler milks are now prominent in the diets of children worldwide, and especially in children living in middle-income countries^(6,29)^. Guidance from WHO deems follow-up and toddler formula unnecessary and unsuitable as BMS and recognises that these products have partially or totally replaced the consumption of breast milk in some countries^(8,30,31)^. Following this guidance, in 2016, the World Health Assembly adopted a new resolution clarifying that BMS, as defined by the International Code of Marketing of Breast milk Substitutes (*The Code*), includes any milk products marketed for feeding infants and children aged 0–36 months^(30)^. As of 2018, however, only forty-four countries restricted the marketing of BMS beyond 12 months of age, and just 16 % of countries with existing provisions of *The Code* in place cover products for 0–36 months^(8)^.

Subsequently, the marketing of BMS for children beyond 12 months of age is not legally restricted in the large majority of countries. Companies have exploited this legislative gap. In the USA, for example, advertising expenditure on toddler milks increased 4-fold between 2006 and 2015 and sales volume increased 2·6-fold, while advertising expenditure and volumes of infant formula declined^(7)^. Another reason for concern is that the branding, packaging and labelling of these products frequently resemble and are often mistaken by parents and caregivers for infant formula^(32)^. Companies use this ‘cross-promotion’ strategy to boost brand loyalty across their entire BMS product range, make actual or implied product claims and indirectly promote infant formula in countries where legislation prohibits this^(30,33)^. The importance of national-level policies and marketing regulations is reinforced by our finding in the multi-level analyses, showing a substantial proportion of the variation in formula use is explained at the country level; in particular, consumption by children in households with the same level of absolute wealth varies markedly depending to the country were the children live.

Furthermore, a long-standing body of evidence demonstrates that the commercial marketing of BMS, through health facilities, in mass and digital media, and via labelling and point-of-sale promotion is highly prevalent, and undermines optimal breast-feeding practices worldwide^(6,34)^. Different levels of exposure to this marketing are likely to at least partially explain the substantial variation in BMS consumption between countries and households with the same level of absolute wealth. This in-turn reflects variations in national-level policy frameworks to promote, support and protect breast-feeding, including the extent to which countries have adopted provisions of *The Code* into national law. Additionally, promotion of BMS among health professionals and facilities negatively affects breast-feeding practices, as women tend to follow practitioners advice and replace the breast milk by BMS^(32)^. This marketing occurs in countries despite national adoption of *The CODE*, being its enforcement urgently needed. In 2020, 136 (70 %) of 194 reporting countries had adopted at least some provisions of *The Code* into national law. However, just 25 (13 %) had enacted a significant set of provisions, 42 (22 %) the majority of provisions, 69 (36 %) less than half of provisions and 58 (30 %) had no restrictions whatsoever^(35)^. Even in countries with legislation in place, violations are frequently reported.

The main drivers of breast-feeding, and conversely of formula feeding, were summarised by Rollins *et al.*^(2)^ These include urbanisation; changing social norms; media influences; the degree of breast-feeding promotion and support in health systems, families, communities and workplaces; and individual-level factors, including mother attributes and relationship between the mother–child dyad^(2)^. The changing relationship between breast-feeding, formula use, the nature of women’s work and income is also crucial^(2)^. Paid employment can make BMS more affordable, and formula can enable the return to work earlier after childbirth. However, millions of working women who want to breastfeed are unable to because of absent or inadequate maternity leave protections and unsupportive working environments. Part of this can be attributed to the miss commitment and lack of obligation by countries in adopting comprehensively the implementation of *The Code* that protects women and children against aggressive BMS marketing and advertisings, enabling women to breastfeed longer^(2,8,32,36)^.

The strengths of our analyses include the large number of countries studied, high comparability in methods and field procedures in all surveys, reliance on feeding data collected through 24-h recall (thus reducing recall bias) and the use of absolute income, given the high cost of formula around the world, especially for most families in LMIC. Absolute income has also been shown to predict other maternal and child health outcomes^(22,37)^. Limitations include the lack of data for high-income and emerging economies countries that represent large markets for formula companies; the exclusion of six surveys (out of the 100 eligible) due to small sample sizes in each wealth quintile, and the lack of information to calculate absolute income for three surveys.

## Conclusion

Globally, there is growing interest in so-called ‘commercial determinants of health’, represented by unhealthy promotion of tobacco, alcohol and ultraprocessed foods, among others. BMS promotion clearly constitutes one type of such determinants^(38)^. Our analyses of breast milk and BMS consumption confirmed that wealth is an important determinant of IYCF practices. However, we also find wide variability in consumption at the same level of household income across countries, and that country level variables may play an important role. This suggests that formula marketing at country level can lead to greater use by all socio-economic groups within a country. Further analyses are required to explore the country level determinants of such variability.

## Supplementary material

For supplementary material accompanying this paper visit https://doi.org/10.1017/S1368980020004061. click here to view supplementary material
