# Supplementary material for: Consumption of breast milk, formula and other non-human milk by children aged under 2 years: analysis of eighty-six low- and middle-income countries
Source: Public Health Nutr. 2020 Oct 16;25(3):680–8. doi: 10.1017/S1368980020004061 (PMC9991621; doi:10.1017/S1368980020004061)
Supplement: Supplementary file 1 [file S1368980020004061sup001.docx]

Title: Consumption of breast milk, formula and other non-human milk by children aged under two years: analysis of 86 low and middle-income countries

**Supplementary figure 1. Flow chart of included surveys**

Most recent survey from 2010 onwards

**100 surveys**

**Excluded surveys (9)**

Lack of data on BF indicators

a. Uruguay 2012

Small sample size per wealth quintile

(n < 25 children)

1. Barbados 2012
2. Bosnia and Herzegovina 2011
3. Macedonia 2011
4. Montenegro 2013
5. Saint Lucia 2012
6. Trinidad and Tobago 2011

Lack of data on household wealth

1. Cuba 2014
2. Qatar 2012

Number of surveys in the ICEH database

(until May 14, 2020)

**389 surveys from 115 countries**

**Excluded surveys (5)**

Unavailable data to generate absolute

dollar values

1. Maldives 2016
2. São Tomé and Príncipe 2014
3. Yemen 2013

Lack of data on formula consumption

1. Mozambique 2015
2. Philippines 2017

Absolute wealth analysis

ICEH – International Center for Equity in Health

BF – Breastfeeding

Included surveys

**86 surveys**

**Supplementary figure 2. Relationship between other non-human milk consumption and absolute income (2011 international dollars, purchasing power parity; top graphs), and the top three and bottom three countries (bottom graphs)^*^ in the prevalence of the indicator by age group. Each dot represents a wealth quintile in each survey (430 dots).**


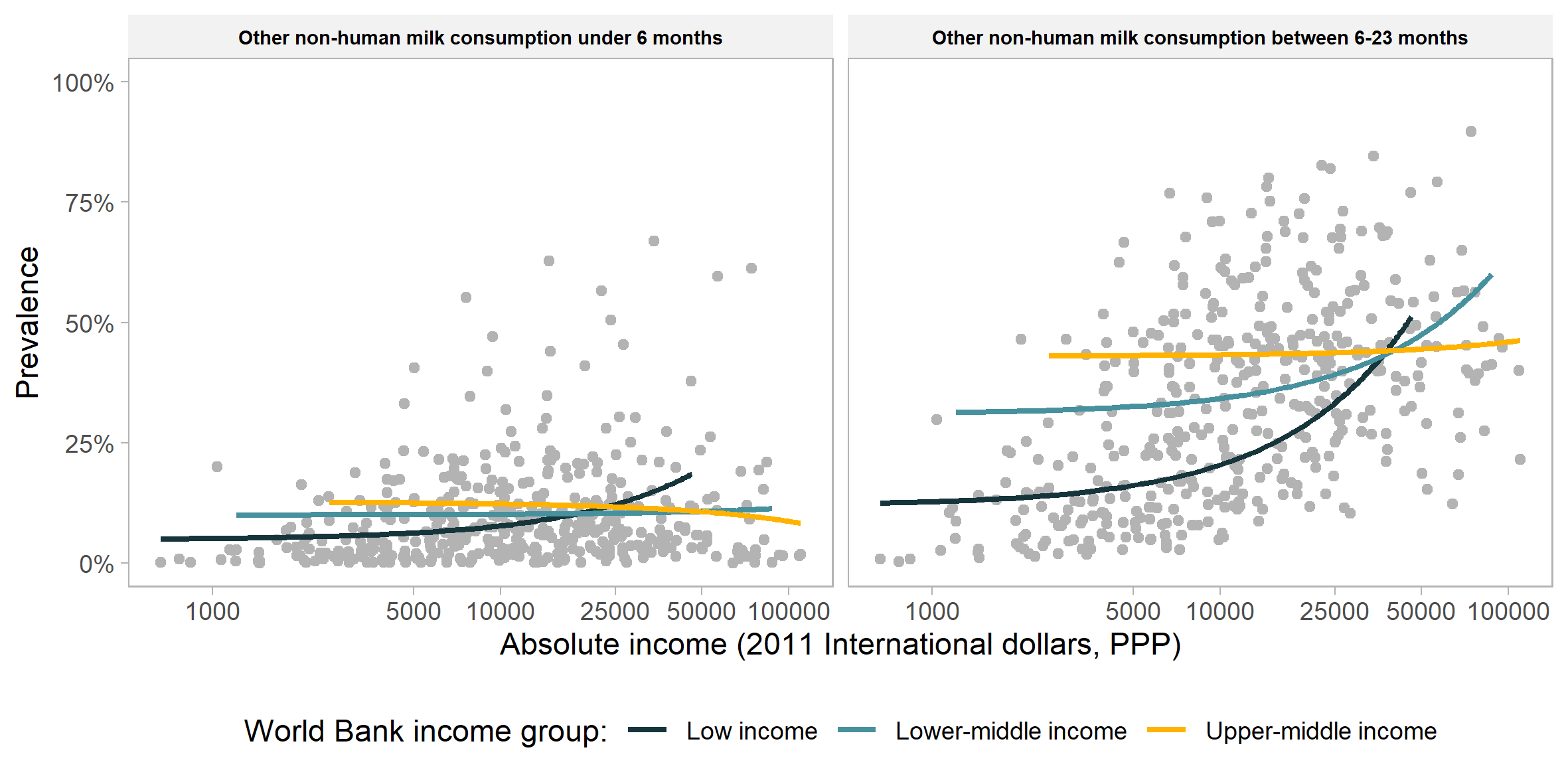


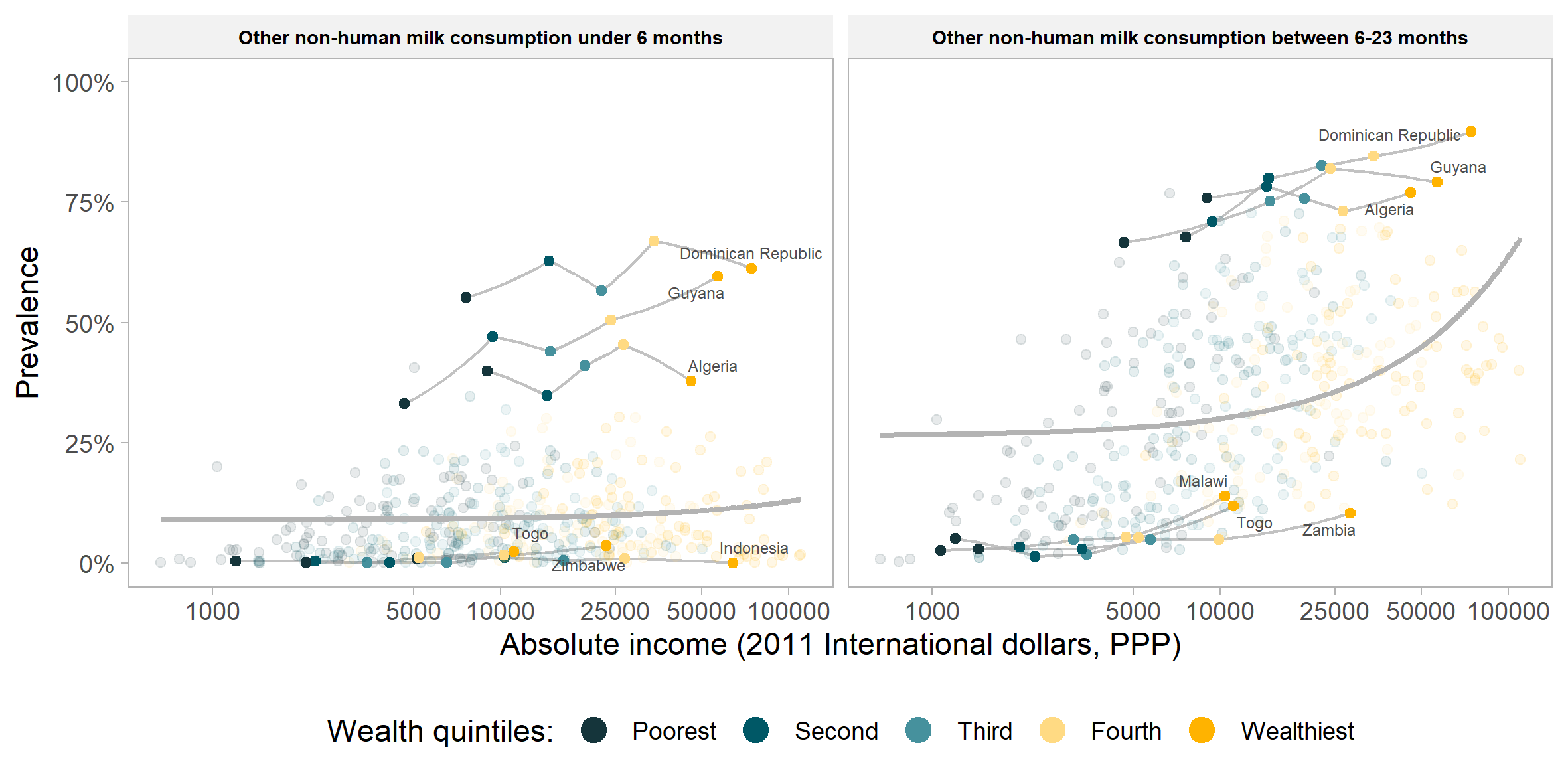


^*^Highlighted countries above the predicted curved represent the three countries with the highest national prevalence of consumption of other non-human milk, and countries below the curve represent the bottom three with the lowest national prevalence of the indicator.

**Supplementary table 1. Countries and surveys included in the breastfeeding and other types of non-human milk (formula and other types of milk, other than formula) inequalities analyses. Source: DHS, MICS, ENSANUT, and ENDES, 2010-2018^*^**

| **Country** | **ISO code^†^** | **Survey and year** | **UNICEF Regions** | **Income Group^‡^** | **Number of children under 6 months** | **Number of children aged 12-15 months** | **Number of children aged 20-23 months** | **Number of children aged 6-23 months** |
| --- | --- | --- | --- | --- | --- | --- | --- | --- |
| Afghanistan | AFG | DHS 2015 | South Asia | LI | 3203 | 2467 | 963 | 8076 |
| Albania | ALB | DHS 2017 | Eastern Europe & Central Asia | UMI | 285 | 188 | 173 | 766 |
| Algeria | DZA | MICS 2012 | Middle East & North Africa | UMI | 1624 | 947 | 936 | 4398 |
| Angola | AGO | DHS 2015 | Eastern & Southern Africa | UMI | 1620 | 944 | 800 | 4008 |
| Argentina | ARG | MICS 2011 | Latin America & Caribbean | UMI | 788 | 615 | 524 | 2638 |
| Armenia | ARM | DHS 2015 | Eastern Europe & Central Asia | LMI | 177 | 118 | 86 | 499 |
| Bangladesh | BGD | DHS 2014 | South Asia | LMI | 632 | 552 | 486 | 2336 |
| Belarus | BLR | MICS 2012 | Eastern Europe & Central Asia | UMI | 247 | 211 | 253 | 1115 |
| Belize | BLZ | MICS 2015 | Latin America & Caribbean | UMI | 165 | 209 | 147 | 730 |
| Benin | BEN | DHS 2017 | West & Central Africa | LI | 1381 | 764 | 819 | 3882 |
| Bhutan | BTN | MICS 2010 | South Asia | LMI | 608 | 423 | 418 | 1928 |
| Burkina Faso | BFA | DHS 2010 | West & Central Africa | LI | 1454 | 986 | 820 | 4148 |
| Burundi | BDI | DHS 2016 | Eastern & Southern Africa | LI | 1236 | 894 | 749 | 3858 |
| CAR^§^ | CAF | MICS 2010 | West & Central Africa | LI | 1283 | 768 | 617 | 3266 |
| Cambodia | KHM | DHS 2014 | East Asia & the Pacific | LI | 688 | 450 | 486 | 2127 |
| Cameroon | CMR | MICS 2014 | West & Central Africa | LMI | 703 | 493 | 436 | 2128 |
| Chad | TCD | DHS 2014 | West & Central Africa | LI | 1823 | 1094 | 713 | 4403 |
| Colombia | COL | DHS 2010 | Latin America & Caribbean | UMI | 1515 | 1089 | 1061 | 5023 |
| Comoros | COM | DHS 2012 | Eastern & Southern Africa | LI | 327 | 210 | 141 | 869 |
| Congo Brazzaville | COG | MICS 2014 | West & Central Africa | LMI | 899 | 567 | 617 | 2765 |
| CDR^\|\|^ | COD | DHS 2013 | West & Central Africa | LI | 1934 | 1294 | 863 | 4991 |
| Costa Rica | CRI | MICS 2011 | Latin America & Caribbean | UMI | 216 | 141 | 150 | 688 |
| Cote d’Ivoire | CIV | MICS 2016 | West & Central Africa | LMI | 981 | 657 | 538 | 2668 |
| Dominican Republic | DOM | MICS 2014 | Latin America & Caribbean | UMI | 1656 | 1082 | 1512 | 6235 |
| Ecuador | ECU | ENSANUT 2012 | Latin America & Caribbean | UMI | 920 | 747 | 624 | 3193 |
| Egypt | EGY | DHS 2014 | Middle East & North Africa | LMI | 1487 | 1018 | 1007 | 4834 |
| El Salvador | SLV | MICS 2014 | Latin America & Caribbean | LMI | 515 | 490 | 539 | 2266 |
| Eswatini | SWZ | MICS 2014 | Eastern & Southern Africa | LMI | 235 | 191 | 171 | 789 |
| Ethiopia | ETH | DHS 2016 | Eastern & Southern Africa | LI | 1092 | 754 | 462 | 2821 |
| Gabon | GAB | DHS 2012 | West & Central Africa | UMI | 631 | 381 | 356 | 1719 |
| Gambia | GMB | DHS 2013 | West & Central Africa | LI | 951 | 640 | 411 | 2423 |
| Ghana | GHA | DHS 2014 | West & Central Africa | LMI | 606 | 362 | 337 | 1656 |
| Guatemala | GTM | DHS 2014 | Latin America & Caribbean | LMI | 1175 | 825 | 715 | 3509 |
| Guinea | GIN | MICS 2016 | West & Central Africa | LI | 686 | 514 | 391 | 2122 |
| Guinea Bissau | GNB | MICS 2014 | West & Central Africa | LI | 830 | 573 | 489 | 2268 |
| Guyana | GUY | MICS 2014 | Latin America & Caribbean | LMI | 290 | 204 | 200 | 1034 |
| Haiti | HTI | DHS 2016 | Latin America & Caribbean | LI | 700 | 425 | 285 | 1652 |
| Honduras | HND | DHS 2011 | Latin America & Caribbean | LMI | 1084 | 769 | 649 | 3237 |
| India | IND | DHS 2015 | South Asia | LMI | 22626 | 16237 | 14274 | 71745 |
| Indonesia | IDN | DHS 2017 | East Asia & the Pacific | LMI | 1666 | 1202 | 1066 | 5033 |
| Iraq | IRQ | MICS 2018 | Middle East & North Africa | UMI | 1681 | 1106 | 990 | 4786 |
| Jamaica | JAM | MICS 2011 | Latin America & Caribbean | UMI | 167 | 118 | 89 | 476 |
| Jordan | JOR | DHS 2017 | Middle East & North Africa | UMI | 1218 | 581 | 536 | 2680 |
| Kazakhstan | KAZ | MICS 2015 | Eastern Europe & Central Asia | UMI | 508 | 381 | 361 | 1632 |
| Kenya | KEN | DHS 2014 | Eastern & Southern Africa | LMI | 856 | 666 | 523 | 2809 |
| Kosovo | XKX | MICS 2013 | Eastern Europe & Central Asia | LMI | 145 | 99 | 104 | 490 |
| Kyrgyzstan | KGZ | MICS 2018 | Eastern Europe & Central Asia | LMI | 390 | 221 | 217 | 992 |
| Lao | LAO | MICS 2017 | East Asia & the Pacific | LMI | 1134 | 755 | 769 | 3428 |
| Lesotho | LSO | DHS 2014 | Eastern & Southern Africa | LMI | 327 | 260 | 170 | 948 |
| Liberia | LBR | DHS 2013 | West & Central Africa | LI | 717 | 472 | 418 | 2155 |
| Malawi | MWI | DHS 2015 | Eastern & Southern Africa | LI | 1636 | 1118 | 1023 | 4747 |
| Mali | MLI | MICS 2015 | West & Central Africa | LI | 1663 | 1290 | 787 | 4865 |
| Mauritania | MRT | MICS 2015 | West & Central Africa | LMI | 915 | 884 | 561 | 3184 |
| Mexico | MEX | MICS 2015 | Latin America & Caribbean | UMI | 666 | 538 | 515 | 2311 |
| Moldova | MDA | MICS 2012 | Eastern Europe & Central Asia | LMI | 176 | 112 | 131 | 591 |
| Mongolia | MNG | MICS 2013 | East Asia & the Pacific | LMI | 644 | 382 | 377 | 1802 |
| Myanmar | MMR | DHS 2015 | East Asia & the Pacific | LMI | 468 | 333 | 262 | 1339 |
| Namibia | NAM | DHS 2013 | Eastern & Southern Africa | UMI | 525 | 311 | 238 | 1302 |
| Nepal | NPL | DHS 2016 | South Asia | LI | 467 | 340 | 347 | 1463 |
| Niger | NER | DHS 2012 | West & Central Africa | LI | 1303 | 926 | 532 | 3259 |
| Nigeria | NGA | MICS 2016 | West & Central Africa | LMI | 2748 | 2042 | 1499 | 8219 |
| Pakistan | PAK | DHS 2017 | South Asia | LMI | 1117 | 701 | 422 | 2565 |
| Panama | PAN | MICS 2013 | Latin America & Caribbean | UMI | 510 | 410 | 415 | 1925 |
| Paraguay | PRY | MICS 2016 | Latin America & Caribbean | UMI | 394 | 334 | 313 | 1443 |
| Peru | PER | ENDES 2018 | Latin America & Caribbean | UMI | 1737 | 1481 | 1407 | 6534 |
| Rwanda | RWA | DHS 2014 | Eastern & Southern Africa | LI | 703 | 502 | 453 | 2353 |
| Senegal | SEN | DHS 2017 | West & Central Africa | LI | 1142 | 734 | 752 | 3489 |
| Serbia | SRB | MICS 2014 | Eastern Europe & Central Asia | UMI | 169 | 146 | 183 | 795 |
| Sierra Leone | SLE | MICS 2017 | West & Central Africa | LI | 1170 | 808 | 716 | 3411 |
| South Africa | ZAF | DHS 2016 | Eastern & Southern Africa | UMI | 346 | 203 | 172 | 877 |
| South Sudan^¶^ | SSD | MICS 2010 | Eastern & Southern Africa | LI | 877 | 799 | 430 | 2553 |
| State of Palestine | PSE | MICS 2014 | Middle East & North Africa | LMI | 665 | 506 | 509 | 2326 |
| Sudan | SDN | MICS 2014 | Middle East & North Africa | LMI | 1543 | 1032 | 731 | 4064 |
| Suriname | SUR | MICS 2018 | Latin America & Caribbean | UMI | 335 | 250 | 241 | 1182 |
| Tajikistan | TJK | DHS 2017 | Eastern Europe & Central Asia | LI | 553 | 416 | 355 | 1722 |
| Tanzania | TZA | DHS 2015 | Eastern & Southern Africa | LI | 1015 | 724 | 586 | 3019 |
| Thailand | THA | MICS 2015 | East Asia & the Pacific | UMI | 661 | 804 | 848 | 3222 |
| Timor Leste | TLS | DHS 2016 | East Asia & the Pacific | LMI | 743 | 491 | 343 | 1950 |
| Togo | TGO | DHS 2013 | West & Central Africa | LI | 603 | 477 | 416 | 2070 |
| Tunisia | TUN | MICS 2018 | Middle East & North Africa | LMI | 299 | 198 | 234 | 946 |
| Turkmenistan | TKM | MICS 2015 | Eastern Europe & Central Asia | UMI | 342 | 270 | 240 | 1169 |
| Uganda | UGA | DHS 2016 | Eastern & Southern Africa | LI | 1482 | 882 | 839 | 4158 |
| Ukraine | UKR | MICS 2012 | Eastern Europe & Central Asia | LMI | 307 | 310 | 276 | 1256 |
| Vietnam | VNM | MICS 2013 | East Asia & the Pacific | LMI | 358 | 265 | 246 | 1118 |
| Zambia | ZMB | DHS 2013 | Eastern & Southern Africa | LMI | 1189 | 863 | 773 | 3722 |
| Zimbabwe | ZWE | DHS 2015 | Eastern & Southern Africa | LI | 603 | 379 | 328 | 1628 |

^*^DHS: Demographic Health Survey; MICS: Multiple Indicator Cluster Survey; ENSANUT: Encuesta Nacional de Salud y Nutrición; ENDES: Encuesta Demográfica y de Salud Familiar; ^†^ISO: International Organization for Standardization; ^‡^LI: Low income country; LMI: Lower-middle income country; UMI: Upper-middle income country. ^§^CAR: Central African Republic; ^||^CDR: Congo Democratic Republic; ^¶^As South Sudan was not classified on their income by the year of the survey implementation (2010), we used its classification in 2014.

**Supplementary table 2. Share of formula consumption over all non-human milk (formula plus other types of non-human milk) consumed by children under two years of age in low and middle-income countries, and by poorest and wealthiest quintiles.**

| **Age of children (months)** | **Share of formula consumption** | | |
| --- | --- | --- | --- |
|  | **National (%)** | **Poorest (%)** | **Wealthiest (%)** |
| Low income countries | | | |
| 0 | 68.8 | 56.8 | 85.5 |
| 6 | 35.0 | 18.2 | 47.7 |
| 12 | 25.9 | 13.5 | 37.2 |
| 24 | 16.9 | 7.5 | 25.3 |
| Lower-middle income countries | | | |
| 0 | 94.5 | 95.6 | 96.2 |
| 6 | 44.8 | 31.0 | 55.4 |
| 12 | 28.2 | 17.5 | 37.7 |
| 24 | 16.2 | 9.4 | 21.9 |
| Upper-middle income countries | | | |
| 0 | 97.3 | 85.9 | 97.4 |
| 6 | 62.9 | 54.0 | 70.5 |
| 12 | 43.8 | 35.5 | 51.4 |
| 24 | 27.7 | 22.3 | 38.6 |
| All low- and middle-income countries | | | |
| 0 | 96.7 | 87.1 | 99.8 |
| 6 | 51.5 | 40.6 | 59.4 |
| 12 | 34.8 | 25.7 | 42.8 |
| 24 | 21.4 | 15.3 | 28.9 |

**Supplementary table 3. Percentage of children at 1 year who were fed breastmilk at the time of the survey by wealth quintiles. Source: DHS, MICS, ENSANUT, and ENDES, 2010-2018^*^**

| **Country** | **Continued breastfeeding at 1 year (12-15 months)** | | | | | | | | | | | | | | | | | | | | |
| --- | --- | --- | --- | --- | --- | --- | --- | --- | --- | --- | --- | --- | --- | --- | --- | --- | --- | --- | --- | --- | --- |
|  | **Wealth quintiles** | | | | | | | | | | | | | | | | | | | | **National prevalence (%)** |
|  | **Poorest** | | | | **Second** | | | | **Third** | | | | **Fourth** | | | | **Wealthiest** | | | |  |
|  | **%** | **95% CI** | | **N** | **%** | **95% CI** | | **N** | **%** | **95% CI** | | **N** | **%** | **95% CI** | | **N** | **%** | **95% CI** | | **N** |  |
| Afghanistan | 86.1 | 81.8 | 89.4 | 403 | 75.1 | 69.6 | 79.8 | 548 | 79.6 | 69.7 | 87.0 | 557 | 77.5 | 71.4 | 82.5 | 571 | 74.5 | 65.5 | 81.9 | 388 | 75.2 |
| Albania | 54.3 | 33.8 | 73.4 | 55 | 63.8 | 49.0 | 76.4 | 56 | 74.5 | 50.1 | 89.4 | 31 | 63.4 | 40.4 | 81.5 | 31 | 32.9 | 10.5 | 67.1 | 15 | 46.7 |
| Algeria | 45.1 | 36.1 | 54.4 | 231 | 47.0 | 37.7 | 56.5 | 197 | 46.4 | 37.6 | 55.4 | 197 | 50.7 | 41.3 | 60.1 | 177 | 44.2 | 35.0 | 53.8 | 145 | 42.5 |
| Angola | 85.8 | 80.6 | 89.8 | 239 | 80.1 | 73.7 | 85.3 | 288 | 87.9 | 82.0 | 92.0 | 225 | 89.0 | 78.8 | 94.6 | 120 | 87.1 | 73.5 | 94.3 | 72 | 82.2 |
| Argentina | 63.3 | 51.3 | 73.8 | 156 | 46.8 | 32.7 | 61.4 | 121 | 43.7 | 28.9 | 59.6 | 113 | 38.6 | 24.8 | 54.5 | 102 | 34.2 | 23.2 | 47.1 | 123 | 40.1 |
| Armenia | 32.2 | 15.6 | 54.8 | 22 | 51.5 | 35.4 | 67.4 | 30 | 31.1 | 16.3 | 51.0 | 21 | 16.4 | 6.9 | 34.5 | 23 | 43.2 | 21.5 | 67.9 | 22 | 27.4 |
| Bangladesh | 97.8 | 90.0 | 99.5 | 125 | 98.4 | 89.1 | 99.8 | 90 | 95.1 | 86.3 | 98.4 | 120 | 93.3 | 81.2 | 97.8 | 107 | 95.7 | 88.7 | 98.4 | 110 | 93.1 |
| Belarus | 32.2 | 13.7 | 58.8 | 26 | 35.3 | 16.4 | 60.2 | 37 | 19.5 | 10.8 | 32.8 | 52 | 21.0 | 10.3 | 38.1 | 37 | 31.5 | 18.5 | 48.1 | 59 | 20.5 |
| Belize | 54.8 | 36.9 | 71.5 | 50 | 59.5 | 40.7 | 75.8 | 52 | 54.2 | 35.5 | 71.8 | 44 | 41.2 | 24.3 | 60.4 | 30 | 40.4 | 23.5 | 59.9 | 33 | 43.8 |
| Benin | 90.0 | 84.1 | 93.9 | 168 | 95.0 | 89.8 | 97.6 | 156 | 95.7 | 91.4 | 97.9 | 163 | 88.9 | 82.4 | 93.2 | 144 | 85.4 | 77.6 | 90.8 | 133 | 88.9 |
| Bhutan | 96.1 | 91.0 | 98.4 | 94 | 91.7 | 83.7 | 96.0 | 107 | 92.3 | 84.4 | 96.3 | 82 | 96.3 | 88.7 | 98.9 | 72 | 87.7 | 76.2 | 94.1 | 68 | 89.6 |
| Burkina Faso | 98.2 | 94.4 | 99.4 | 178 | 95.6 | 92.1 | 97.6 | 216 | 98.7 | 95.7 | 99.6 | 213 | 98.1 | 95.0 | 99.3 | 216 | 92.3 | 85.5 | 96.1 | 163 | 95.4 |
| Burundi | 96.1 | 91.6 | 98.2 | 179 | 95.6 | 91.2 | 97.9 | 195 | 97.2 | 93.3 | 98.8 | 171 | 95.8 | 91.1 | 98.1 | 170 | 89.3 | 82.5 | 93.6 | 179 | 93.2 |
| CAR^†^ | 90.7 | 83.7 | 94.8 | 209 | 85.0 | 78.0 | 90.0 | 186 | 91.2 | 84.6 | 95.1 | 171 | 92.0 | 84.6 | 96.0 | 130 | 88.6 | 75.6 | 95.1 | 72 | 86.4 |
| Cambodia | 84.4 | 74.8 | 90.7 | 120 | 84.7 | 74.3 | 91.4 | 91 | 92.6 | 82.1 | 97.1 | 65 | 82.3 | 69.8 | 90.3 | 73 | 49.9 | 35.6 | 64.2 | 101 | 75.1 |
| Cameroon | 84.9 | 68.7 | 93.5 | 80 | 88.7 | 81.1 | 93.4 | 118 | 71.6 | 59.9 | 80.9 | 100 | 67.0 | 56.3 | 76.2 | 110 | 25.1 | 16.5 | 36.2 | 85 | 64.9 |
| Chad | 88.2 | 81.5 | 92.6 | 218 | 86.3 | 80.5 | 90.6 | 258 | 90.4 | 85.2 | 94.0 | 235 | 90.3 | 85.0 | 93.9 | 226 | 83.2 | 74.9 | 89.2 | 157 | 85.4 |
| Colombia | 64.0 | 56.6 | 70.8 | 381 | 62.8 | 55.2 | 69.9 | 314 | 62.8 | 53.9 | 70.9 | 172 | 50.7 | 41.3 | 60.1 | 136 | 45.5 | 33.6 | 57.9 | 86 | 54.6 |
| Comoros | 75.4 | 61.2 | 85.6 | 51 | 62.1 | 46.4 | 75.6 | 50 | 67.8 | 48.3 | 82.5 | 43 | 75.3 | 51.9 | 89.6 | 30 | 70.2 | 52.0 | 83.7 | 36 | 61.9 |
| Congo Brazzaville | 80.1 | 74.5 | 84.7 | 262 | 73.0 | 60.9 | 82.5 | 148 | 54.1 | 38.3 | 69.1 | 77 | 40.5 | 27.2 | 55.4 | 50 | 53.3 | 30.5 | 74.8 | 30 | 55.3 |
| CDR^‡^ | 92.5 | 88.7 | 95.1 | 346 | 96.0 | 92.6 | 97.9 | 296 | 91.1 | 85.0 | 94.9 | 272 | 89.9 | 84.4 | 93.6 | 229 | 88.9 | 81.8 | 93.5 | 151 | 89.9 |
| Costa Rica | 63.1 | 39.0 | 82.1 | 38 | 75.2 | 50.8 | 89.9 | 40 | 56.2 | 21.8 | 85.5 | 23 | 42.4 | 16.1 | 73.8 | 29 | 12.3 | 1.7 | 53.9 | 11 | 39.2 |
| Cote d’Ivoire | 94.7 | 90.6 | 97.0 | 230 | 88.9 | 79.4 | 94.4 | 164 | 90.0 | 77.6 | 95.9 | 132 | 91.5 | 75.3 | 97.4 | 78 | 67.6 | 54.5 | 78.4 | 53 | 84.3 |
| Dominican Republic | 40.1 | 33.0 | 47.7 | 378 | 28.6 | 21.3 | 37.2 | 215 | 29.7 | 20.9 | 40.4 | 202 | 24.4 | 16.0 | 35.4 | 155 | 28.6 | 15.5 | 46.7 | 132 | 27.0 |
| Ecuador | 73.7 | 63.6 | 81.8 | 226 | 65.5 | 51.7 | 77.1 | 177 | 58.5 | 45.1 | 70.6 | 155 | 65.4 | 48.8 | 78.9 | 111 | 61.2 | 44.6 | 75.5 | 78 | 59.7 |
| Egypt | 86.4 | 79.4 | 91.2 | 178 | 77.9 | 69.7 | 84.4 | 171 | 85.0 | 77.8 | 90.2 | 222 | 76.5 | 69.3 | 82.5 | 204 | 72.7 | 65.2 | 79.1 | 243 | 76.9 |
| El Salvador | 87.6 | 75.1 | 94.3 | 114 | 75.7 | 62.9 | 85.1 | 105 | 72.4 | 59.2 | 82.5 | 96 | 68.1 | 51.3 | 81.1 | 84 | 60.0 | 45.6 | 72.8 | 91 | 68.2 |
| Eswatini | 58.6 | 43.5 | 72.2 | 53 | 59.0 | 42.0 | 74.0 | 37 | 51.6 | 35.4 | 67.4 | 42 | 41.1 | 23.9 | 60.7 | 30 | 27.4 | 12.1 | 50.8 | 29 | 39.7 |
| Ethiopia | 91.4 | 86.3 | 94.8 | 290 | 90.7 | 79.9 | 95.9 | 119 | 92.7 | 83.8 | 96.9 | 110 | 94.5 | 84.1 | 98.2 | 96 | 89.1 | 80.1 | 94.4 | 139 | 88.8 |
| Gabon | 64.9 | 56.9 | 72.1 | 192 | 54.1 | 36.4 | 70.8 | 71 | 28.9 | 14.5 | 49.3 | 55 | 44.8 | 29.8 | 60.8 | 35 | 30.2 | 12.0 | 57.9 | 28 | 37.9 |
| Gambia | 96.9 | 90.0 | 99.1 | 171 | 97.6 | 92.8 | 99.2 | 167 | 100.0 | - | - | 129 | 98.9 | 94.2 | 99.8 | 103 | 95.2 | 84.3 | 98.6 | 70 | 95.7 |
| Ghana | 98.0 | 92.2 | 99.5 | 132 | 96.6 | 89.4 | 99.0 | 67 | 93.7 | 80.8 | 98.1 | 51 | 90.8 | 74.2 | 97.1 | 61 | 94.0 | 84.1 | 97.9 | 51 | 90.6 |
| Guatemala | 94.2 | 89.5 | 96.8 | 228 | 92.4 | 87.8 | 95.4 | 197 | 84.8 | 76.4 | 90.6 | 174 | 81.4 | 72.9 | 87.7 | 135 | 58.6 | 46.9 | 69.4 | 91 | 82.2 |
| Guinea | 95.4 | 89.5 | 98.1 | 125 | 97.4 | 92.3 | 99.2 | 116 | 94.2 | 88.0 | 97.3 | 108 | 96.7 | 89.6 | 99.0 | 92 | 80.7 | 68.3 | 89.0 | 73 | 90.5 |
| Guinea Bissau | 99.2 | 96.8 | 99.8 | 147 | 96.1 | 90.8 | 98.4 | 156 | 94.9 | 88.2 | 97.9 | 151 | 93.5 | 83.8 | 97.6 | 83 | 84.2 | 69.0 | 92.7 | 36 | 91.1 |
| Guyana | 77.0 | 65.8 | 85.4 | 72 | 53.9 | 34.4 | 72.4 | 42 | 53.1 | 29.5 | 75.4 | 41 | 36.7 | 16.4 | 63.2 | 21 | 35.3 | 19.2 | 55.6 | 28 | 45.9 |
| Haiti | 77.9 | 67.3 | 85.8 | 128 | 82.1 | 71.3 | 89.4 | 100 | 74.4 | 60.2 | 84.8 | 98 | 87.2 | 63.5 | 96.4 | 53 | 62.2 | 47.0 | 75.3 | 46 | 71.0 |
| Honduras | 87.7 | 81.6 | 92.0 | 256 | 77.9 | 70.0 | 84.2 | 187 | 65.9 | 56.5 | 74.1 | 143 | 56.3 | 44.3 | 67.7 | 108 | 48.4 | 35.8 | 61.2 | 75 | 64.9 |
| India | 90.7 | 89.5 | 91.8 | 4293 | 89.4 | 87.9 | 90.7 | 3772 | 85.7 | 83.8 | 87.3 | 3242 | 82.6 | 80.4 | 84.5 | 2725 | 78.8 | 76.0 | 81.3 | 2205 | 85.4 |
| Indonesia | 85.8 | 80.8 | 89.7 | 323 | 83.0 | 77.4 | 87.5 | 244 | 73.8 | 66.3 | 80.2 | 212 | 75.5 | 68.7 | 81.3 | 219 | 63.2 | 54.7 | 70.9 | 204 | 73.6 |
| Iraq | 56.8 | 48.3 | 64.9 | 294 | 46.3 | 36.4 | 56.4 | 232 | 44.7 | 32.0 | 58.1 | 224 | 41.7 | 32.2 | 51.8 | 197 | 33.5 | 21.9 | 47.4 | 159 | 39.4 |
| Jamaica | 44.8 | 26.3 | 64.9 | 37 | 52.5 | 31.3 | 72.8 | 23 | 41.8 | 19.2 | 68.4 | 21 | 46.8 | 24.8 | 70.2 | 21 | 32.3 | 8.0 | 72.2 | 16 | 34.1 |
| Jordan | 36.3 | 27.9 | 45.6 | 222 | 30.2 | 22.1 | 39.8 | 152 | 43.1 | 31.1 | 55.9 | 134 | 24.2 | 11.4 | 44.3 | 51 | 47.3 | 23.0 | 72.9 | 22 | 30.6 |
| Kazakhstan | 52.7 | 39.8 | 65.2 | 76 | 62.1 | 47.2 | 75.1 | 72 | 61.3 | 49.0 | 72.2 | 94 | 56.2 | 42.2 | 69.2 | 75 | 67.5 | 50.3 | 81.0 | 64 | 53.6 |
| Kenya | 90.2 | 85.0 | 93.7 | 253 | 91.0 | 83.1 | 95.4 | 130 | 88.7 | 79.3 | 94.1 | 112 | 88.0 | 74.4 | 94.8 | 103 | 95.3 | 86.7 | 98.4 | 68 | 86.8 |
| Kosovo | 58.1 | 39.0 | 75.0 | 24 | 69.9 | 47.9 | 85.4 | 26 | 42.5 | 22.7 | 65.0 | 19 | 47.2 | 22.4 | 73.4 | 14 | 59.5 | 34.7 | 80.3 | 16 | 45.8 |
| Kyrgyzstan | 88.2 | 70.7 | 95.9 | 35 | 80.1 | 64.4 | 89.9 | 40 | 86.3 | 72.7 | 93.8 | 49 | 76.2 | 61.4 | 86.5 | 58 | 55.3 | 40.7 | 69.1 | 39 | 70.1 |
| Lao | 80.6 | 74.1 | 85.7 | 229 | 72.8 | 64.4 | 79.9 | 175 | 66.6 | 56.9 | 75.0 | 137 | 45.8 | 36.1 | 55.9 | 120 | 45.0 | 34.3 | 56.2 | 94 | 60.7 |
| Lesotho | 88.9 | 78.6 | 94.6 | 73 | 71.1 | 54.2 | 83.6 | 51 | 76.2 | 61.6 | 86.5 | 58 | 60.7 | 39.9 | 78.3 | 38 | 51.3 | 34.3 | 68.1 | 40 | 64.4 |
| Liberia | 95.7 | 91.7 | 97.8 | 167 | 88.6 | 79.7 | 93.9 | 124 | 96.5 | 89.4 | 98.9 | 79 | 82.0 | 68.7 | 90.4 | 63 | 75.0 | 55.7 | 87.8 | 39 | 82.8 |
| Malawi | 93.3 | 89.4 | 95.8 | 269 | 90.3 | 85.4 | 93.7 | 242 | 90.6 | 85.1 | 94.2 | 208 | 90.3 | 84.9 | 94.0 | 204 | 93.1 | 86.7 | 96.5 | 195 | 89.6 |
| Mali | 91.8 | 86.7 | 95.1 | 268 | 92.6 | 88.6 | 95.4 | 271 | 93.9 | 89.5 | 96.5 | 255 | 91.1 | 84.9 | 94.8 | 253 | 91.6 | 86.8 | 94.7 | 243 | 90.3 |
| Mauritania | 81.7 | 73.9 | 87.6 | 197 | 90.5 | 85.4 | 93.9 | 201 | 86.6 | 80.1 | 91.2 | 195 | 92.0 | 84.7 | 96.0 | 163 | 76.1 | 66.4 | 83.7 | 128 | 82.4 |
| Mexico | 70.7 | 58.6 | 80.5 | 165 | 39.8 | 28.5 | 52.2 | 147 | 50.4 | 32.8 | 67.9 | 106 | 24.9 | 13.1 | 42.1 | 76 | 26.6 | 12.9 | 47.0 | 44 | 39.1 |
| Moldova | 67.3 | 43.2 | 84.8 | 21 | 33.6 | 14.4 | 60.3 | 17 | 44.7 | 21.3 | 70.8 | 16 | 44.9 | 22.7 | 69.3 | 19 | 46.3 | 28.2 | 65.4 | 39 | 38.4 |
| Mongolia | 85.5 | 76.0 | 91.6 | 84 | 90.6 | 80.4 | 95.8 | 66 | 89.4 | 80.5 | 94.5 | 78 | 80.7 | 69.6 | 88.4 | 74 | 70.4 | 58.9 | 79.7 | 80 | 78.1 |
| Myanmar | 92.7 | 83.1 | 97.1 | 99 | 89.1 | 78.1 | 94.9 | 81 | 85.6 | 69.0 | 94.1 | 51 | 85.3 | 71.2 | 93.1 | 61 | 82.0 | 60.5 | 93.1 | 41 | 83.2 |
| Namibia | 73.7 | 59.8 | 84.1 | 69 | 76.3 | 63.3 | 85.7 | 67 | 73.9 | 61.2 | 83.5 | 74 | 52.7 | 37.6 | 67.2 | 60 | 31.2 | 17.2 | 49.7 | 41 | 58.4 |
| Nepal | 97.2 | 89.9 | 99.3 | 90 | 100.0 | - | - | 59 | 97.5 | 84.1 | 99.7 | 75 | 97.9 | 91.1 | 99.5 | 75 | 98.3 | 88.6 | 99.8 | 41 | 95.7 |
| Niger | 94.1 | 89.3 | 96.9 | 182 | 93.1 | 87.4 | 96.4 | 176 | 87.3 | 79.9 | 92.3 | 167 | 94.6 | 90.0 | 97.2 | 182 | 93.5 | 88.8 | 96.3 | 219 | 90.3 |
| Nigeria | 90.6 | 86.3 | 93.6 | 474 | 90.7 | 87.5 | 93.2 | 448 | 86.2 | 79.7 | 90.8 | 394 | 81.0 | 76.0 | 85.2 | 372 | 78.5 | 73.2 | 83.0 | 354 | 84.0 |
| Pakistan | 80.1 | 71.8 | 86.4 | 159 | 78.1 | 66.6 | 86.4 | 127 | 71.6 | 60.5 | 80.5 | 139 | 60.8 | 48.9 | 71.4 | 126 | 57.5 | 46.4 | 67.9 | 150 | 64.7 |
| Panama | 69.9 | 57.7 | 79.8 | 205 | 58.2 | 40.1 | 74.3 | 80 | 56.7 | 35.1 | 76.0 | 57 | 29.8 | 14.4 | 51.6 | 48 | 17.9 | 3.3 | 58.0 | 20 | 45.3 |
| Paraguay | 63.5 | 50.3 | 74.9 | 95 | 34.0 | 20.4 | 51.0 | 69 | 57.0 | 41.0 | 71.7 | 52 | 40.6 | 27.3 | 55.5 | 77 | 54.3 | 32.6 | 74.4 | 41 | 40.5 |
| Peru | 93.3 | 89.7 | 95.6 | 466 | 87.5 | 82.6 | 91.1 | 363 | 77.3 | 70.3 | 83.1 | 296 | 80.4 | 72.5 | 86.5 | 205 | 71.5 | 61.5 | 79.8 | 151 | 80.9 |
| Rwanda | 97.4 | 92.3 | 99.2 | 123 | 98.0 | 92.2 | 99.5 | 108 | 98.1 | 92.4 | 99.5 | 94 | 97.3 | 89.6 | 99.4 | 91 | 84.3 | 69.5 | 92.7 | 86 | 92.7 |
| Senegal | 95.9 | 91.7 | 98.0 | 230 | 96.2 | 91.9 | 98.2 | 201 | 92.8 | 87.6 | 95.9 | 150 | 95.0 | 84.9 | 98.5 | 95 | 90.1 | 74.2 | 96.6 | 58 | 91.6 |
| Serbia | 36.6 | 14.1 | 67.0 | 20 | 8.’7 | 2.0 | 31.0 | 23 | 20.3 | 8.9 | 39.9 | 32 | 24.6 | 12.0 | 43.7 | 31 | 30.8 | 15.4 | 52.0 | 40 | 17.1 |
| Sierra Leone | 89.6 | 84.4 | 93.2 | 245 | 85.7 | 80.0 | 90.0 | 201 | 90.0 | 84.0 | 93.8 | 181 | 87.2 | 76.2 | 93.6 | 106 | 65.0 | 52.5 | 75.7 | 75 | 81.7 |
| South Africa | 62.3 | 46.5 | 75.8 | 60 | 41.6 | 27.2 | 57.5 | 48 | 44.2 | 27.1 | 62.7 | 44 | 59.9 | 39.9 | 77.0 | 35 | 44.2 | 19.7 | 71.9 | 16 | 42.5 |
| South Sudan | 88.7 | 81.8 | 93.2 | 162 | 84.7 | 77.5 | 89.9 | 165 | 80.2 | 72.1 | 86.4 | 154 | 81.7 | 74.8 | 87.1 | 178 | 74.5 | 64.8 | 82.3 | 140 | 78.8 |
| State of Palestine | 65.6 | 55.7 | 74.3 | 108 | 60.1 | 49.7 | 69.7 | 92 | 48.0 | 39.2 | 56.9 | 115 | 41.8 | 32.7 | 51.6 | 115 | 47.2 | 36.0 | 58.6 | 76 | 48.3 |
| Sudan | 84.7 | 77.9 | 89.7 | 247 | 90.5 | 86.1 | 93.6 | 278 | 91.6 | 86.6 | 94.8 | 242 | 86.7 | 77.5 | 92.5 | 150 | 94.5 | 86.8 | 97.8 | 115 | 86.8 |
| Suriname | 47.4 | 32.3 | 63.0 | 66 | 33.0 | 20.9 | 47.9 | 66 | 30.9 | 14.7 | 53.5 | 48 | 23.9 | 9.5 | 48.7 | 38 | 25.6 | 9.3 | 53.6 | 32 | 27.1 |
| Tajikistan | 79.1 | 67.5 | 87.3 | 81 | 67.8 | 52.3 | 80.2 | 63 | 66.9 | 54.7 | 77.2 | 94 | 80.5 | 70.8 | 87.5 | 83 | 65.9 | 54.2 | 75.9 | 95 | 67.2 |
| Tanzania | 92.0 | 86.8 | 95.3 | 159 | 86.2 | 77.9 | 91.7 | 151 | 95.1 | 89.0 | 97.9 | 127 | 92.7 | 86.7 | 96.2 | 170 | 95.8 | 89.7 | 98.4 | 117 | 89.4 |
| Thailand | 46.6 | 31.8 | 62.0 | 183 | 35.3 | 22.8 | 50.3 | 194 | 34.1 | 16.2 | 57.9 | 153 | 22.1 | 12.0 | 37.3 | 155 | 14.9 | 7.7 | 26.7 | 119 | 25.6 |
| Timor Leste | 74.1 | 63.5 | 82.5 | 93 | 70.3 | 60.3 | 78.6 | 115 | 68.8 | 56.5 | 78.9 | 94 | 49.0 | 37.8 | 60.3 | 105 | 50.4 | 36.0 | 64.8 | 84 | 56.7 |
| Togo | 96.1 | 91.9 | 98.2 | 157 | 93.1 | 85.8 | 96.8 | 101 | 96.0 | 89.6 | 98.6 | 87 | 93.7 | 84.5 | 97.6 | 65 | 90.6 | 81.5 | 95.4 | 67 | 91.3 |
| Tunisia | 70.0 | 55.0 | 81.7 | 47 | 33.8 | 19.9 | 51.3 | 45 | 59.7 | 39.9 | 76.7 | 30 | 33.2 | 19.6 | 50.4 | 41 | 33.8 | 19.6 | 51.7 | 35 | 37.8 |
| Turkmenistan | 82.0 | 66.5 | 91.3 | 40 | 71.3 | 55.4 | 83.2 | 40 | 63.3 | 49.1 | 75.6 | 63 | 61.9 | 47.6 | 74.3 | 69 | 40.3 | 27.5 | 54.7 | 58 | 57.1 |
| Uganda | 94.1 | 90.1 | 96.6 | 248 | 89.4 | 82.6 | 93.7 | 189 | 84.4 | 77.0 | 89.8 | 154 | 85.5 | 78.6 | 90.5 | 165 | 78.6 | 69.5 | 85.6 | 126 | 84.2 |
| Ukraine | 39.2 | 24.4 | 56.3 | 54 | 41.6 | 28.7 | 55.6 | 84 | 39.2 | 17.8 | 65.7 | 47 | 37.8 | 20.7 | 58.6 | 62 | 32.8 | 18.8 | 50.8 | 63 | 29.6 |
| Vietnam | 81.2 | 67.2 | 90.1 | 64 | 78.9 | 63.9 | 88.8 | 47 | 69.5 | 51.9 | 82.8 | 45 | 58.2 | 43.3 | 71.8 | 56 | 38.9 | 25.6 | 54.1 | 53 | 59.0 |
| Zambia | 96.3 | 92.0 | 98.4 | 214 | 97.3 | 94.0 | 98.8 | 213 | 94.2 | 88.5 | 97.2 | 198 | 90.9 | 82.8 | 95.4 | 130 | 77.1 | 65.0 | 85.9 | 108 | 89.6 |
| Zimbabwe | 90.6 | 81.0 | 95.6 | 90 | 94.1 | 85.7 | 97.7 | 79 | 94.9 | 86.1 | 98.3 | 55 | 92.8 | 86.2 | 96.4 | 90 | 79.7 | 63.9 | 89.6 | 65 | 87.5 |

^*^DHS: Demographic Health Survey; MICS: Multiple Indicator Cluster Survey; ENSANUT: Encuesta Nacional de Salud y Nutrición; ENDES: Encuesta Demográfica y de Salud Familiar; ^†^CAR: Central African Republic; ^‡^CDR: Congo Democratic Republic.

**Supplementary table 4. Percentage of children at 2 years who were fed breastmilk at the time of the survey by wealth quintiles. Source: DHS, MICS, ENSANUT, and ENDES, 2010-2018.^a^**

| **Country** | **Continued breastfeeding at 2 years (20-23 months)** | | | | | | | | | | | | | | | | | | | | |
| --- | --- | --- | --- | --- | --- | --- | --- | --- | --- | --- | --- | --- | --- | --- | --- | --- | --- | --- | --- | --- | --- |
|  | **Wealth quintiles** | | | | | | | | | | | | | | | | | | | | **National prevalence (%)** |
|  | **Poorest** | | | | **Second** | | | | **Third** | | | | **Fourth** | | | | **Wealthiest** | | | |  |
|  | **%** | **95% CI** | | **N** | **%** | **95% CI** | | **N** | **%** | **95% CI** | | **N** | **%** | **95% CI** | | **N** | **%** | **95% CI** | | **N** |  |
| Afghanistan | 62.4 | 51.9 | 71.9 | 182 | 63.7 | 54.3 | 72.1 | 209 | 52.1 | 43.5 | 60.6 | 202 | 58.5 | 44.9 | 70.9 | 222 | 55.1 | 40.9 | 68.5 | 148 | 58.6 |
| Albania | 31.8 | 20.7 | 45.3 | 63 | 40.1 | 22.8 | 60.3 | 32 | 17.1 | 7.7 | 33.8 | 37 | 31.0 | 15.8 | 51.8 | 28 | 28.4 | 8.8 | 62.1 | 13 | 29.7 |
| Algeria | 26.2 | 19.5 | 34.3 | 215 | 28.2 | 21.0 | 36.8 | 192 | 25.3 | 17.6 | 34.9 | 183 | 27.4 | 19.9 | 36.5 | 201 | 25.6 | 15.9 | 38.4 | 145 | 26.6 |
| Angola | 52.4 | 43.7 | 60.9 | 167 | 49.8 | 41.3 | 58.3 | 231 | 42.7 | 34.2 | 51.6 | 214 | 34.9 | 20.7 | 52.4 | 117 | 24.4 | 14.6 | 38.0 | 71 | 41.6 |
| Argentina | 30.4 | 21.1 | 41.8 | 155 | 36.3 | 22.7 | 52.5 | 98 | 23.0 | 13.7 | 36.0 | 108 | 19.8 | 11.3 | 32.6 | 93 | 34.9 | 12.4 | 67.0 | 70 | 29.1 |
| Armenia | 36.5 | 14.1 | 66.9 | 16 | 25.4 | 7.3 | 59.6 | 14 | 32.3 | 15.5 | 55.3 | 28 | 5.7 | 0.7 | 33.5 | 14 | 0.0 | - | - | 14 | 21.6 |
| Bangladesh | 93.2 | 85.6 | 96.9 | 93 | 93.6 | 84.4 | 97.6 | 96 | 85.1 | 73.8 | 92.0 | 104 | 85.9 | 75.7 | 92.3 | 105 | 78.4 | 65.4 | 87.5 | 88 | 87.3 |
| Belarus | 20.1 | 9.4 | 37.8 | 41 | 6.4 | 1.8 | 20.4 | 44 | 8.6 | 1.9 | 31.7 | 52 | 14.2 | 5.1 | 33.9 | 50 | 11.8 | 4.6 | 26.9 | 66 | 11.5 |
| Belize | 48.5 | 27.8 | 69.7 | 32 | 36.4 | 19.3 | 57.9 | 31 | 28.6 | 12.6 | 52.5 | 20 | 34.1 | 18.1 | 54.8 | 39 | 25.6 | 11.0 | 48.9 | 25 | 35.1 |
| Benin | 57.9 | 49.0 | 66.3 | 171 | 58.2 | 49.6 | 66.3 | 162 | 43.7 | 35.8 | 51.8 | 182 | 31.1 | 24.5 | 38.6 | 163 | 15.7 | 10.0 | 23.8 | 141 | 42.4 |
| Bhutan | 77.2 | 65.4 | 85.9 | 83 | 70.4 | 58.6 | 80.0 | 93 | 68.4 | 55.5 | 79.0 | 84 | 58.2 | 45.2 | 70.2 | 99 | 57.6 | 44.0 | 70.2 | 59 | 65.7 |
| Burkina Faso | 83.1 | 76.1 | 88.4 | 150 | 88.8 | 82.7 | 92.9 | 167 | 86.0 | 80.7 | 90.0 | 203 | 79.7 | 72.4 | 85.5 | 166 | 54.5 | 43.5 | 65.2 | 134 | 80.1 |
| Burundi | 86.9 | 80.3 | 91.5 | 158 | 88.2 | 82.6 | 92.2 | 165 | 76.6 | 69.1 | 82.7 | 172 | 75.7 | 66.6 | 83.0 | 120 | 75.2 | 66.1 | 82.6 | 134 | 81.4 |
| CAR^†^ | 41.7 | 32.7 | 51.2 | 136 | 45.4 | 35.1 | 56.1 | 172 | 29.7 | 21.3 | 39.7 | 133 | 15.0 | 8.1 | 26.0 | 106 | 22.3 | 13.6 | 34.4 | 70 | 32.1 |
| Cambodia | 48.4 | 36.4 | 60.6 | 121 | 33.9 | 21.7 | 48.6 | 75 | 38.6 | 26.5 | 52.3 | 75 | 34.7 | 22.7 | 49.0 | 88 | 24.9 | 15.8 | 37.0 | 127 | 37.1 |
| Cameroon | 49.1 | 36.7 | 61.7 | 54 | 23.9 | 15.2 | 35.5 | 106 | 6.3 | 2.5 | 14.6 | 99 | 14.7 | 8.2 | 25.1 | 98 | 3.6 | 1.2 | 10.3 | 79 | 18.5 |
| Chad | 72.9 | 62.2 | 81.4 | 135 | 63.9 | 54.0 | 72.8 | 153 | 63.4 | 54.7 | 71.3 | 149 | 73.1 | 62.8 | 81.4 | 147 | 49.9 | 41.4 | 58.4 | 129 | 65.2 |
| Colombia | 38.0 | 31.7 | 44.6 | 389 | 32.7 | 26.0 | 40.1 | 274 | 35.2 | 27.6 | 43.6 | 205 | 27.6 | 19.5 | 37.4 | 117 | 22.7 | 13.9 | 34.9 | 76 | 32.5 |
| Comoros | 62.2 | 42.7 | 78.4 | 32 | 37.1 | 17.7 | 61.8 | 28 | 65.4 | 44.7 | 81.5 | 29 | 55.4 | 34.8 | 74.3 | 29 | 68.4 | 42.7 | 86.2 | 23 | 56.7 |
| Congo Brazzaville | 26.6 | 20.4 | 33.9 | 274 | 10.5 | 6.1 | 17.3 | 154 | 2.2 | 0.7 | 7.0 | 86 | 6.2 | 2.1 | 16.7 | 60 | 7.9 | 2.2 | 24.8 | 43 | 11.2 |
| CDR^‡^ | 75.2 | 64.9 | 83.2 | 207 | 73.0 | 64.0 | 80.4 | 189 | 72.7 | 61.7 | 81.5 | 197 | 68.7 | 57.9 | 77.9 | 154 | 34.4 | 25.2 | 44.9 | 116 | 66.3 |
| Costa Rica | 46.0 | 25.8 | 67.6 | 46 | 27.5 | 12.3 | 50.7 | 48 | 13.6 | 3.7 | 38.8 | 27 | 12.1 | 2.8 | 40.3 | 20 | 31.4 | 4.7 | 80.9 | 9 | 27.5 |
| Cote d’Ivoire | 42.4 | 32.8 | 52.7 | 154 | 37.4 | 26.9 | 49.3 | 138 | 24.1 | 16.5 | 33.9 | 114 | 24.3 | 14.6 | 37.6 | 82 | 8.0 | 2.9 | 19.9 | 50 | 29.0 |
| Dominican Republic | 16.2 | 11.8 | 21.8 | 471 | 10.3 | 7.0 | 14.8 | 341 | 10.5 | 6.7 | 16.0 | 272 | 13.1 | 6.1 | 25.7 | 230 | 11.1 | 4.1 | 26.9 | 198 | 12.4 |
| Ecuador | 35.5 | 26.0 | 46.3 | 198 | 34.2 | 23.9 | 46.2 | 147 | 38.7 | 23.7 | 56.3 | 111 | 13.6 | 6.2 | 27.0 | 99 | 14.0 | 6.6 | 27.0 | 69 | 28.0 |
| Egypt | 30.2 | 22.8 | 38.8 | 154 | 23.3 | 17.1 | 30.9 | 176 | 20.3 | 14.8 | 27.2 | 210 | 18.6 | 12.5 | 26.7 | 229 | 12.0 | 7.2 | 19.1 | 238 | 20.4 |
| El Salvador | 55.6 | 44.9 | 65.8 | 122 | 62.0 | 50.7 | 72.2 | 127 | 57.2 | 45.3 | 68.2 | 111 | 60.5 | 47.7 | 72.0 | 105 | 44.3 | 29.3 | 60.3 | 74 | 57.0 |
| Eswatini | 0.9 | 0.1 | 6.3 | 48 | 14.4 | 6.3 | 29.6 | 43 | 4.4 | 1.1 | 16.4 | 39 | 13.3 | 3.1 | 42.3 | 22 | 7.4 | 2.3 | 21.8 | 19 | 7.6 |
| Ethiopia | 76.7 | 61.1 | 87.3 | 126 | 74.8 | 59.5 | 85.7 | 94 | 79.0 | 65.6 | 88.1 | 74 | 69.3 | 55.1 | 80.6 | 57 | 77.4 | 58.6 | 89.2 | 111 | 75.5 |
| Gabon | 9.7 | 5.8 | 15.8 | 170 | 6.8 | 2.3 | 18.3 | 80 | 0.6 | 0.1 | 4.2 | 49 | 0.0 | - | - | 34 | 0.0 | - | - | 23 | 3.9 |
| Gambia | 44.7 | 34.6 | 55.3 | 96 | 56.7 | 46.3 | 66.5 | 118 | 37.7 | 28.4 | 48.0 | 97 | 40.0 | 24.4 | 58.0 | 58 | 27.4 | 14.0 | 46.6 | 42 | 42.2 |
| Ghana | 78.9 | 68.0 | 86.8 | 104 | 52.8 | 40.0 | 65.1 | 97 | 57.7 | 37.5 | 75.7 | 53 | 26.9 | 14.2 | 45.1 | 50 | 23.6 | 10.2 | 45.7 | 33 | 50.1 |
| Guatemala | 70.9 | 62.7 | 78.0 | 189 | 63.7 | 55.4 | 71.3 | 167 | 57.7 | 47.8 | 67.1 | 150 | 44.2 | 34.5 | 54.4 | 127 | 34.8 | 23.8 | 47.8 | 82 | 56.8 |
| Guinea | 83.7 | 72.7 | 90.8 | 79 | 69.6 | 55.0 | 81.1 | 82 | 71.9 | 60.2 | 81.2 | 78 | 52.0 | 41.2 | 62.6 | 82 | 25.8 | 16.2 | 38.6 | 70 | 59.8 |
| Guinea Bissau | 61.9 | 52.6 | 70.4 | 149 | 62.9 | 52.2 | 72.4 | 120 | 49.2 | 37.8 | 60.7 | 108 | 44.2 | 34.1 | 54.9 | 72 | 26.0 | 13.6 | 44.1 | 40 | 50.9 |
| Guyana | 51.8 | 38.8 | 64.6 | 83 | 40.4 | 23.7 | 59.7 | 36 | 31.2 | 15.7 | 52.3 | 27 | 39.6 | 19.5 | 64.1 | 30 | 26.2 | 12.6 | 46.7 | 24 | 40.9 |
| Haiti | 36.1 | 26.2 | 47.4 | 108 | 37.2 | 25.7 | 50.3 | 64 | 19.4 | 9.8 | 34.6 | 47 | 7.0 | 1.9 | 22.3 | 42 | 8.4 | 1.7 | 33.7 | 24 | 24.9 |
| Honduras | 61.9 | 54.2 | 69.1 | 205 | 48.7 | 39.9 | 57.7 | 155 | 36.4 | 27.4 | 46.6 | 119 | 36.2 | 26.6 | 47.1 | 101 | 22.2 | 13.5 | 34.3 | 69 | 43.3 |
| India | 82.5 | 80.8 | 84.2 | 3619 | 77.6 | 75.7 | 79.4 | 3244 | 71.2 | 68.7 | 73.5 | 2923 | 63.1 | 60.0 | 66.1 | 2451 | 58.4 | 54.7 | 62.0 | 2037 | 71.7 |
| Indonesia | 61.0 | 53.2 | 68.4 | 285 | 54.4 | 46.0 | 62.6 | 197 | 63.5 | 54.9 | 71.3 | 187 | 49.8 | 41.5 | 58.1 | 201 | 45.2 | 37.0 | 53.6 | 196 | 54.6 |
| Iraq | 32.6 | 25.8 | 40.3 | 284 | 20.0 | 12.9 | 29.7 | 240 | 20.9 | 14.0 | 30.0 | 177 | 27.1 | 18.1 | 38.5 | 164 | 35.6 | 24.4 | 48.5 | 125 | 26.7 |
| Jamaica | 20.2 | 9.0 | 39.3 | 21 | 27.6 | 11.0 | 54.1 | 18 | 52.3 | 26.1 | 77.4 | 20 | 17.9 | 5.3 | 45.7 | 17 | 32.9 | 10.7 | 66.8 | 13 | 31.2 |
| Jordan | 14.4 | 9.0 | 22.2 | 212 | 16.7 | 9.2 | 28.3 | 127 | 15.7 | 8.3 | 27.8 | 121 | 7.5 | 2.3 | 21.5 | 52 | 20.8 | 7.4 | 46.3 | 24 | 14.9 |
| Kazakhstan | 30.3 | 19.3 | 44.0 | 77 | 6.4 | 2.4 | 16.1 | 57 | 17.3 | 10.3 | 27.5 | 85 | 30.1 | 19.2 | 44.0 | 76 | 25.6 | 16.0 | 38.2 | 66 | 21.1 |
| Kenya | 54.9 | 46.1 | 63.3 | 177 | 49.8 | 39.7 | 59.9 | 118 | 51.1 | 37.3 | 64.8 | 75 | 58.1 | 43.0 | 71.8 | 77 | 51.9 | 37.3 | 66.2 | 76 | 53.1 |
| Kosovo | 41.2 | 21.3 | 64.5 | 22 | 30.5 | 15.0 | 52.1 | 24 | 24.4 | 10.2 | 47.8 | 22 | 43.0 | 24.3 | 63.9 | 21 | 18.4 | 5.4 | 47.1 | 15 | 31.8 |
| Kyrgyzstan | 30.8 | 17.4 | 48.5 | 49 | 14.7 | 7.1 | 28.0 | 45 | 29.3 | 13.1 | 53.2 | 37 | 21.1 | 11.3 | 35.9 | 54 | 15.0 | 6.5 | 31.1 | 32 | 22.4 |
| Lao | 51.0 | 42.1 | 59.9 | 183 | 36.6 | 28.9 | 45.1 | 197 | 24.8 | 17.8 | 33.5 | 137 | 11.2 | 6.1 | 19.4 | 137 | 5.9 | 2.7 | 12.5 | 115 | 27.2 |
| Lesotho | 52.0 | 35.8 | 67.8 | 43 | 40.1 | 23.7 | 59.1 | 38 | 25.3 | 12.1 | 45.5 | 30 | 24.1 | 10.5 | 46.3 | 34 | 3.3 | 0.5 | 19.5 | 25 | 29.5 |
| Liberia | 47.3 | 36.3 | 58.5 | 158 | 56.2 | 43.8 | 67.9 | 108 | 42.6 | 30.3 | 55.9 | 77 | 35.7 | 21.6 | 52.7 | 52 | 37.0 | 18.6 | 60.1 | 23 | 44.2 |
| Malawi | 80.7 | 74.8 | 85.6 | 243 | 74.1 | 66.8 | 80.2 | 225 | 73.5 | 65.1 | 80.5 | 219 | 60.4 | 51.0 | 69.1 | 159 | 59.8 | 47.5 | 71.0 | 177 | 71.5 |
| Mali | 49.8 | 40.6 | 59.0 | 132 | 57.1 | 48.1 | 65.6 | 135 | 64.4 | 55.4 | 72.4 | 146 | 54.5 | 46.1 | 62.6 | 187 | 42.8 | 34.9 | 51.0 | 187 | 53.4 |
| Mauritania | 55.5 | 42.6 | 67.7 | 86 | 41.9 | 31.7 | 52.9 | 106 | 34.3 | 25.1 | 44.8 | 120 | 37.2 | 27.4 | 48.1 | 127 | 33.5 | 25.1 | 43.2 | 122 | 39.6 |
| Mexico | 31.1 | 20.1 | 44.7 | 168 | 36.2 | 23.0 | 51.9 | 123 | 18.6 | 10.5 | 30.8 | 103 | 14.6 | 8.2 | 24.7 | 85 | 8.1 | 2.2 | 25.3 | 36 | 24.4 |
| Moldova | 18.8 | 3.7 | 58.0 | 11 | 8.1 | 1.9 | 28.4 | 26 | 6.6 | 1.5 | 24.5 | 19 | 20.2 | 6.6 | 47.6 | 31 | 9.1 | 3.3 | 22.6 | 44 | 12.2 |
| Mongolia | 52.9 | 42.7 | 62.8 | 102 | 49.9 | 37.6 | 62.3 | 65 | 74.2 | 61.3 | 84.0 | 59 | 54.2 | 42.4 | 65.5 | 75 | 38.5 | 28.1 | 50.1 | 76 | 52.9 |
| Myanmar | 71.6 | 53.2 | 84.9 | 70 | 76.0 | 59.2 | 87.4 | 54 | 74.8 | 58.8 | 86.1 | 52 | 49.5 | 31.9 | 67.2 | 46 | 44.6 | 27.3 | 63.4 | 40 | 63.8 |
| Namibia | 21.6 | 12.4 | 34.9 | 49 | 14.3 | 7.2 | 26.4 | 59 | 24.6 | 13.8 | 40.0 | 45 | 30.8 | 16.9 | 49.5 | 49 | 13.5 | 6.6 | 25.8 | 36 | 21.1 |
| Nepal | 98.0 | 91.9 | 99.5 | 71 | 89.1 | 79.3 | 94.6 | 93 | 84.6 | 73.8 | 91.5 | 87 | 87.6 | 75.7 | 94.2 | 62 | 82.4 | 54.6 | 94.8 | 34 | 88.5 |
| Niger | 67.9 | 54.2 | 79.1 | 64 | 59.5 | 47.1 | 70.9 | 80 | 54.4 | 44.6 | 63.9 | 95 | 42.5 | 33.1 | 52.4 | 119 | 37.7 | 29.0 | 47.2 | 174 | 50.2 |
| Nigeria | 56.7 | 49.5 | 63.7 | 288 | 53.7 | 46.8 | 60.5 | 326 | 39.4 | 32.1 | 47.2 | 284 | 18.5 | 13.6 | 24.7 | 308 | 17.0 | 12.5 | 22.6 | 293 | 37.1 |
| Pakistan | 72.1 | 60.4 | 81.3 | 85 | 59.5 | 44.7 | 72.8 | 86 | 44.2 | 32.8 | 56.3 | 95 | 52.3 | 35.8 | 68.3 | 71 | 37.9 | 25.0 | 52.8 | 85 | 53.4 |
| Panama | 47.9 | 35.8 | 60.2 | 213 | 35.8 | 22.1 | 52.2 | 79 | 26.4 | 11.7 | 49.3 | 56 | 23.8 | 12.8 | 40.0 | 47 | 12.3 | 3.2 | 37.6 | 20 | 34.3 |
| Paraguay | 30.4 | 18.2 | 46.0 | 88 | 11.0 | 3.9 | 27.4 | 62 | 13.9 | 7.2 | 25.2 | 64 | 18.3 | 9.8 | 31.6 | 58 | 37.9 | 20.7 | 58.8 | 41 | 21.0 |
| Peru | 50.1 | 44.5 | 55.6 | 388 | 46.6 | 40.1 | 53.2 | 363 | 45.8 | 38.1 | 53.8 | 287 | 54.3 | 45.6 | 62.9 | 206 | 57.4 | 47.5 | 66.8 | 163 | 50.3 |
| Rwanda | 90.8 | 84.0 | 94.9 | 112 | 88.4 | 78.8 | 93.9 | 77 | 89.0 | 80.9 | 94.0 | 96 | 86.4 | 75.0 | 93.1 | 75 | 79.8 | 69.6 | 87.2 | 93 | 87.2 |
| Senegal | 52.8 | 45.3 | 60.2 | 221 | 37.7 | 30.0 | 46.0 | 179 | 36.5 | 28.1 | 45.9 | 171 | 36.2 | 25.5 | 48.4 | 104 | 34.1 | 21.6 | 49.3 | 77 | 40.0 |
| Serbia | 20.2 | 8.2 | 41.6 | 25 | 17.5 | 6.5 | 39.5 | 27 | 4.3 | 1.2 | 14.7 | 40 | 6.3 | 1.8 | 20.1 | 46 | 4.9 | 1.3 | 16.5 | 45 | 8.9 |
| Sierra Leone | 49.8 | 41.3 | 58.4 | 211 | 51.9 | 43.8 | 59.9 | 166 | 36.6 | 28.4 | 45.7 | 154 | 33.3 | 22.6 | 46.0 | 102 | 13.0 | 6.0 | 26.0 | 83 | 38.2 |
| South Africa | 10.1 | 4.3 | 22.2 | 46 | 6.0 | 1.8 | 18.0 | 35 | 12.0 | 4.9 | 26.5 | 33 | 21.0 | 6.6 | 50.2 | 34 | 16.3 | 2.9 | 55.7 | 24 | 13.0 |
| South Sudan | 39.4 | 27.7 | 52.4 | 76 | 37.2 | 26.3 | 49.5 | 80 | 37.0 | 27.0 | 48.3 | 91 | 38.9 | 29.2 | 49.5 | 98 | 37.5 | 26.7 | 49.7 | 85 | 38.0 |
| State of Palestine | 6.7 | 3.0 | 14.5 | 116 | 8.1 | 3.9 | 15.9 | 86 | 18.1 | 12.4 | 25.6 | 110 | 12.5 | 7.5 | 20.2 | 109 | 12.5 | 7.3 | 20.6 | 88 | 11.5 |
| Sudan | 45.7 | 34.4 | 57.6 | 120 | 49.0 | 40.4 | 57.7 | 189 | 55.2 | 43.5 | 66.3 | 158 | 48.2 | 37.2 | 59.4 | 157 | 44.7 | 33.9 | 55.9 | 107 | 48.8 |
| Suriname | 16.7 | 8.5 | 30.4 | 59 | 6.5 | 2.6 | 15.4 | 54 | 15.9 | 6.7 | 33.0 | 50 | 14.7 | 4.7 | 37.3 | 50 | 10.5 | 3.3 | 28.7 | 28 | 13.4 |
| Tajikistan | 32.2 | 20.3 | 47.0 | 52 | 38.8 | 27.7 | 51.3 | 74 | 36.2 | 25.5 | 48.5 | 79 | 39.3 | 26.3 | 54.0 | 64 | 41.6 | 29.9 | 54.4 | 86 | 37.6 |
| Tanzania | 54.6 | 45.0 | 64.0 | 144 | 42.8 | 32.4 | 53.9 | 116 | 42.8 | 32.9 | 53.3 | 109 | 38.1 | 27.9 | 49.4 | 117 | 33.7 | 23.7 | 45.5 | 100 | 43.5 |
| Thailand | 18.9 | 10.6 | 31.4 | 172 | 19.5 | 9.7 | 35.4 | 189 | 17.4 | 8.7 | 31.9 | 191 | 10.7 | 5.2 | 20.8 | 191 | 8.9 | 4.2 | 17.9 | 105 | 15.6 |
| Timor Leste | 53.0 | 38.9 | 66.6 | 52 | 42.6 | 29.2 | 57.0 | 61 | 38.5 | 27.6 | 50.5 | 79 | 33.4 | 21.2 | 48.3 | 84 | 36.2 | 23.4 | 51.3 | 67 | 39.8 |
| Togo | 83.0 | 71.3 | 90.6 | 104 | 75.9 | 64.7 | 84.4 | 85 | 66.4 | 53.5 | 77.2 | 85 | 52.3 | 40.1 | 64.3 | 70 | 33.1 | 21.9 | 46.7 | 72 | 61.4 |
| Tunisia | 15.0 | 7.5 | 27.6 | 56 | 21.7 | 11.9 | 36.3 | 53 | 21.4 | 11.0 | 37.4 | 41 | 11.1 | 4.4 | 25.4 | 52 | 23.5 | 11.5 | 42.1 | 32 | 18.2 |
| Turkmenistan | 26.4 | 15.3 | 41.5 | 39 | 19.9 | 9.5 | 36.9 | 41 | 22.4 | 11.1 | 39.9 | 45 | 22.7 | 13.2 | 36.2 | 55 | 6.3 | 1.5 | 23.2 | 60 | 19.5 |
| Uganda | 55.5 | 47.6 | 63.1 | 209 | 48.1 | 40.6 | 55.8 | 195 | 49.0 | 40.3 | 57.7 | 154 | 31.6 | 23.0 | 41.7 | 134 | 31.3 | 24.2 | 39.5 | 147 | 43.3 |
| Ukraine | 16.9 | 8.3 | 31.4 | 63 | 16.4 | 7.6 | 31.7 | 61 | 13.9 | 5.1 | 32.6 | 40 | 29.5 | 10.9 | 58.9 | 58 | 27.4 | 10.8 | 54.1 | 54 | 22.0 |
| Vietnam | 24.7 | 14.1 | 39.6 | 61 | 31.0 | 16.6 | 50.2 | 44 | 22.3 | 11.2 | 39.5 | 37 | 18.7 | 8.6 | 35.8 | 53 | 13.3 | 6.5 | 25.3 | 51 | 21.8 |
| Zambia | 65.8 | 58.2 | 72.7 | 186 | 49.9 | 41.2 | 58.6 | 190 | 37.8 | 30.0 | 46.4 | 175 | 27.8 | 19.6 | 37.8 | 135 | 7.8 | 3.1 | 18.2 | 87 | 41.8 |
| Zimbabwe | 21.0 | 12.9 | 32.3 | 77 | 8.6 | 3.4 | 20.1 | 55 | 15.7 | 7.9 | 28.9 | 65 | 9.7 | 4.9 | 18.4 | 85 | 14.3 | 4.3 | 38.2 | 46 | 14.2 |

^*^DHS: Demographic Health Survey; MICS: Multiple Indicator Cluster Survey; ENSANUT: Encuesta Nacional de Salud y Nutrición; ENDES: Encuesta Demográfica y de Salud Familiar; ^†^CAR: Central African Republic; ^‡^CDR: Congo Democratic Republic.

**Supplementary table 5. Percentage of children under 6 months who were fed formula at the time of the survey by wealth quintiles. Source: DHS, MICS, ENSANUT, and ENDES, 2010-2018.^a^**

| **Country** | **Infant formula consumption under 6 months (0-5 months)** | | | | | | | | | | | | | | | | | | | | |
| --- | --- | --- | --- | --- | --- | --- | --- | --- | --- | --- | --- | --- | --- | --- | --- | --- | --- | --- | --- | --- | --- |
|  | **Wealth quintiles** | | | | | | | | | | | | | | | | | | | | **National prevalence (%)** |
|  | **Poorest** | | | | **Second** | | | | **Third** | | | | **Fourth** | | | | **Wealthiest** | | | |  |
|  | **%** | **95% CI** | | **N** | **%** | **95% CI** | | **N** | **%** | **95% CI** | | **N** | **%** | **95% CI** | | **N** | **%** | **95% CI** | | **N** |  |
| Afghanistan | 4.2 | 2.6 | 6.7 | 528 | 7.9 | 4.9 | 12.4 | 754 | 7.4 | 5.0 | 10.6 | 759 | 9.4 | 6.5 | 13.4 | 710 | 13.8 | 9.0 | 20.5 | 452 | 8.5 |
| Albania | 14.7 | 7.4 | 26.9 | 84 | 27.6 | 14.5 | 46.0 | 60 | 18.8 | 7.8 | 38.6 | 55 | 33.8 | 20.5 | 50.4 | 59 | 24.2 | 8.8 | 51.5 | 27 | 24.0 |
| Algeria | 11.5 | 7.6 | 16.9 | 399 | 12.5 | 7.2 | 20.7 | 344 | 8.6 | 5.6 | 12.8 | 316 | 10.7 | 6.9 | 16.2 | 312 | 10.1 | 6.6 | 15.2 | 253 | 10.8 |
| Angola | 1.3 | 0.5 | 3.5 | 413 | 1.7 | 0.7 | 4.1 | 448 | 5.5 | 2.4 | 12.0 | 418 | 12.6 | 7.8 | 19.6 | 211 | 19.7 | 12.7 | 29.2 | 130 | 7.0 |
| Argentina | 29.0 | 21.0 | 38.5 | 214 | 35.6 | 23.9 | 49.3 | 143 | 50.8 | 39.1 | 62.4 | 159 | 57.0 | 44.1 | 69.1 | 149 | 32.8 | 20.8 | 47.5 | 123 | 39.0 |
| Armenia | 7.4 | 2.1 | 23.2 | 28 | 4.5 | 1.1 | 16.4 | 37 | 2.5 | 0.3 | 16.5 | 39 | 2.4 | 0.4 | 14.1 | 35 | 1.7 | 0.2 | 11.1 | 38 | 3.4 |
| Bangladesh | 9.5 | 3.9 | 21.6 | 122 | 3.5 | 1.2 | 9.3 | 130 | 5.9 | 2.2 | 14.7 | 121 | 14.4 | 8.3 | 23.8 | 120 | 17.4 | 10.5 | 27.6 | 139 | 10.1 |
| Belarus | 46.3 | 25.5 | 68.6 | 36 | 57.0 | 38.2 | 74.0 | 51 | 45.5 | 25.8 | 66.7 | 41 | 62.6 | 44.0 | 78.1 | 52 | 43.5 | 29.4 | 58.7 | 67 | 50.3 |
| Belize | 27.9 | 13.0 | 50.1 | 40 | 34.0 | 19.1 | 53.0 | 37 | 51.9 | 29.9 | 73.2 | 34 | 42.9 | 22.3 | 66.3 | 33 | 58.6 | 33.6 | 79.8 | 21 | 42.0 |
| Benin | 1.7 | 0.7 | 4.4 | 311 | 2.6 | 1.2 | 5.9 | 285 | 2.0 | 0.7 | 5.6 | 281 | 3.1 | 1.6 | 6.0 | 251 | 9.0 | 5.9 | 13.4 | 253 | 3.5 |
| Bhutan | 2.8 | 0.5 | 14.0 | 123 | 2.9 | 0.8 | 9.6 | 115 | 5.3 | 2.5 | 10.7 | 149 | 11.6 | 6.0 | 21.1 | 119 | 9.2 | 3.7 | 21.4 | 102 | 6.7 |
| Burkina Faso | 0.0 | - | - | 275 | 0.0 | - | - | 324 | 0.7 | 0.2 | 2.9 | 329 | 0.0 | - | - | 318 | 4.2 | 1.9 | 9.0 | 208 | 0.8 |
| Burundi | 0.0 | - | - | 233 | 0.0 | - | - | 241 | 0.0 | - | - | 222 | 0.0 | - | - | 236 | 2.7 | 1.2 | 5.8 | 304 | 0.5 |
| CAR^†^ | 1.4 | 0.6 | 3.3 | 277 | 4.4 | 2.4 | 8.2 | 314 | 5.2 | 3.1 | 8.6 | 325 | 7.3 | 3.9 | 13.4 | 233 | 11.2 | 5.9 | 20.4 | 134 | 5.6 |
| Cambodia | 4.2 | 1.7 | 9.8 | 156 | 5.0 | 2.2 | 10.8 | 121 | 8.3 | 4.3 | 15.4 | 119 | 9.6 | 5.6 | 16.0 | 134 | 34.2 | 24.5 | 45.3 | 158 | 11.5 |
| Cameroon | 0.0 | - | - | 117 | 2.0 | 0.8 | 5.0 | 153 | 4.9 | 2.6 | 9.1 | 174 | 13.6 | 6.6 | 26.0 | 147 | 32.3 | 23.7 | 42.4 | 112 | 8.7 |
| Chad | 0.9 | 0.4 | 2.4 | 355 | 0.4 | 0.1 | 0.8 | 356 | 0.7 | 0.3 | 1.9 | 375 | 1.2 | 0.5 | 2.9 | 405 | 21.8 | 17.6 | 26.6 | 332 | 4.7 |
| Colombia | 28.1 | 22.8 | 34.0 | 567 | 29.8 | 24.3 | 35.8 | 413 | 43.1 | 36.1 | 50.4 | 282 | 36.0 | 27.8 | 45.1 | 171 | 46.1 | 34.1 | 58.5 | 82 | 35.2 |
| Comoros | 7.7 | 3.5 | 16.0 | 89 | 30.6 | 18.3 | 46.5 | 66 | 24.0 | 14.7 | 36.6 | 61 | 35.5 | 23.7 | 49.4 | 54 | 43.3 | 28.7 | 59.2 | 57 | 26.5 |
| Congo Brazzaville | 4.6 | 2.7 | 7.7 | 432 | 9.0 | 4.1 | 18.4 | 219 | 10.0 | 4.7 | 20.2 | 112 | 15.6 | 7.6 | 29.3 | 78 | 15.6 | 7.8 | 28.9 | 58 | 10.4 |
| CDR^‡^ | 0.2 | 0.0 | 1.4 | 535 | 0.0 | 0.0 | 0.1 | 434 | 0.6 | 0.2 | 2.1 | 369 | 3.1 | 1.2 | 7.3 | 333 | 13.3 | 9.0 | 19.3 | 263 | 3.2 |
| Costa Rica | 34.2 | 20.6 | 51.0 | 90 | 40.2 | 16.2 | 70.1 | 48 | 28.5 | 11.6 | 54.9 | 32 | 65.5 | 36.7 | 86.2 | 24 | 33.1 | 13.5 | 61.1 | 22 | 38.0 |
| Cote d’Ivoire | 0.1 | 0.0 | 0.9 | 316 | 2.2 | 0.5 | 9.6 | 253 | 4.3 | 1.9 | 9.7 | 219 | 10.1 | 3.8 | 24.0 | 119 | 20.1 | 11.4 | 32.9 | 74 | 5.6 |
| Dominican Republic | 22.0 | 16.2 | 29.3 | 552 | 26.5 | 19.7 | 34.7 | 377 | 36.2 | 25.9 | 48.0 | 293 | 30.7 | 23.3 | 39.2 | 253 | 42.2 | 32.0 | 53.1 | 181 | 30.0 |
| Ecuador | 17.1 | 10.7 | 26.2 | 317 | 23.2 | 15.0 | 34.1 | 224 | 36.9 | 24.5 | 51.3 | 170 | 25.3 | 15.1 | 39.3 | 117 | 46.4 | 30.1 | 63.5 | 92 | 27.9 |
| Egypt | 15.4 | 11.2 | 20.6 | 299 | 12.2 | 8.3 | 17.5 | 263 | 14.4 | 10.5 | 19.3 | 324 | 16.1 | 11.7 | 21.7 | 314 | 21.3 | 15.8 | 28.0 | 287 | 15.6 |
| El Salvador | 18.7 | 11.5 | 28.9 | 144 | 31.8 | 20.9 | 45.2 | 104 | 35.4 | 23.2 | 49.8 | 98 | 37.9 | 24.8 | 53.0 | 86 | 39.5 | 24.8 | 56.3 | 83 | 31.0 |
| Eswatini | 5.4 | 1.9 | 14.4 | 65 | 6.5 | 2.3 | 16.7 | 59 | 18.8 | 9.7 | 33.2 | 56 | 19.2 | 6.2 | 46.0 | 30 | 37.1 | 19.1 | 59.7 | 25 | 15.1 |
| Ethiopia | 0.2 | 0.0 | 0.9 | 418 | 0.2 | 0.0 | 1.2 | 174 | 0.1 | 0.0 | 0.7 | 129 | 0.1 | 0.0 | 0.4 | 145 | 5.3 | 2.3 | 11.9 | 226 | 0.9 |
| Gabon | 41.6 | 35.0 | 48.5 | 300 | 66.3 | 53.2 | 77.2 | 137 | 73.4 | 60.6 | 83.2 | 88 | 64.5 | 43.8 | 80.9 | 60 | 84.2 | 63.1 | 94.3 | 46 | 65.3 |
| Gambia | 0.5 | 0.1 | 3.8 | 230 | 0.2 | 0.0 | 1.1 | 235 | 0.9 | 0.2 | 3.6 | 192 | 5.8 | 2.8 | 11.7 | 177 | 3.9 | 1.1 | 12.5 | 117 | 2.2 |
| Ghana | 0.0 | - | - | 210 | 5.3 | 2.0 | 13.3 | 121 | 10.9 | 5.6 | 20.2 | 112 | 15.6 | 8.9 | 25.8 | 91 | 21.1 | 12.1 | 34.3 | 72 | 9.7 |
| Guatemala | 3.4 | 1.8 | 6.3 | 318 | 8.0 | 5.2 | 11.9 | 287 | 20.3 | 15.0 | 26.8 | 222 | 40.3 | 32.1 | 49.0 | 206 | 56.3 | 46.2 | 65.9 | 142 | 20.9 |
| Guinea | 0.0 | - | - | 168 | 3.4 | 1.5 | 7.6 | 162 | 5.6 | 2.3 | 13.2 | 139 | 10.0 | 5.8 | 16.7 | 133 | 21.6 | 13.2 | 33.3 | 84 | 7.2 |
| Guinea Bissau | 0.9 | 0.3 | 2.6 | 260 | 0.6 | 0.1 | 3.9 | 209 | 0.5 | 0.1 | 3.2 | 198 | 1.8 | 0.3 | 11.8 | 94 | 6.9 | 2.6 | 17.1 | 69 | 1.8 |
| Guyana | 19.3 | 10.0 | 34.0 | 114 | 52.7 | 34.8 | 69.8 | 59 | 54.2 | 36.0 | 71.3 | 50 | 59.0 | 36.9 | 77.9 | 33 | 64.0 | 44.7 | 79.6 | 34 | 45.3 |
| Haiti | 8.0 | 4.4 | 14.3 | 200 | 13.4 | 8.4 | 20.7 | 189 | 17.7 | 11.6 | 26.0 | 141 | 26.1 | 17.5 | 36.9 | 105 | 45.7 | 33.0 | 59.1 | 65 | 18.9 |
| Honduras | 5.8 | 3.6 | 9.1 | 341 | 15.0 | 10.6 | 20.8 | 270 | 33.4 | 25.4 | 42.4 | 185 | 39.6 | 31.4 | 48.5 | 178 | 46.4 | 34.9 | 58.2 | 110 | 26.1 |
| India | 2.0 | 1.5 | 2.6 | 5829 | 3.6 | 2.7 | 4.8 | 5494 | 3.9 | 3.1 | 4.9 | 4508 | 4.5 | 3.6 | 5.5 | 3660 | 5.4 | 4.2 | 6.8 | 3135 | 3.7 |
| Indonesia | 13.8 | 10.5 | 18.0 | 469 | 24.5 | 19.3 | 30.5 | 346 | 33.4 | 27.6 | 39.8 | 309 | 35.2 | 28.5 | 42.5 | 290 | 40.0 | 32.6 | 47.8 | 252 | 28.9 |
| Iraq | 31.6 | 25.9 | 38.0 | 439 | 47.9 | 41.1 | 54.7 | 387 | 44.3 | 37.5 | 51.4 | 352 | 46.3 | 39.2 | 53.5 | 275 | 55.0 | 46.9 | 62.7 | 228 | 44.5 |
| Jamaica | 37.2 | 21.9 | 55.6 | 28 | 30.9 | 17.3 | 48.9 | 39 | 68.9 | 53.1 | 81.3 | 47 | 59.9 | 39.4 | 77.5 | 30 | 65.5 | 40.3 | 84.2 | 23 | 52.9 |
| Jordan | 40.0 | 32.5 | 48.1 | 441 | 48.2 | 40.2 | 56.3 | 312 | 55.5 | 45.7 | 64.8 | 241 | 53.9 | 44.8 | 62.7 | 164 | 55.2 | 37.7 | 71.5 | 60 | 49.5 |
| Kazakhstan | 14.3 | 8.3 | 23.7 | 82 | 25.0 | 14.2 | 40.1 | 90 | 22.9 | 15.2 | 33.1 | 142 | 12.8 | 7.5 | 21.1 | 102 | 23.1 | 11.7 | 40.5 | 92 | 20.0 |
| Kenya | 0.8 | 0.1 | 4.8 | 320 | 0.0 | - | - | 167 | 0.9 | 0.2 | 3.8 | 140 | 0.8 | 0.2 | 3.2 | 119 | 0.5 | 0.1 | 2.4 | 110 | 0.6 |
| Kosovo | 21.2 | 10.7 | 37.6 | 38 | 18.9 | 7.4 | 40.5 | 24 | 22.5 | 9.9 | 43.5 | 28 | 38.4 | 20.0 | 60.8 | 25 | 20.3 | 8.3 | 41.7 | 30 | 23.8 |
| Kyrgyzstan | 13.9 | 6.4 | 27.6 | 78 | 11.6 | 5.4 | 22.9 | 72 | 16.7 | 9.2 | 28.5 | 76 | 15.0 | 7.3 | 28.3 | 104 | 10.5 | 4.2 | 23.9 | 60 | 13.6 |
| Lao | 2.6 | 1.0 | 6.7 | 348 | 8.1 | 4.8 | 13.2 | 250 | 16.6 | 11.4 | 23.5 | 213 | 22.6 | 15.8 | 31.4 | 171 | 39.4 | 30.5 | 49.2 | 152 | 15.9 |
| Lesotho | 4.4 | 1.7 | 11.0 | 82 | 9.2 | 4.1 | 19.4 | 76 | 10.9 | 4.9 | 22.4 | 67 | 23.5 | 13.0 | 38.8 | 59 | 49.3 | 27.1 | 71.8 | 43 | 17.4 |
| Liberia | 0.9 | 0.2 | 3.4 | 256 | 0.4 | 0.1 | 1.7 | 194 | 6.1 | 2.1 | 16.3 | 148 | 9.9 | 4.1 | 22.1 | 77 | 12.6 | 5.1 | 27.9 | 42 | 5.1 |
| Malawi | 2.6 | 1.0 | 6.4 | 379 | 0.9 | 0.3 | 2.4 | 358 | 0.7 | 0.2 | 2.2 | 317 | 2.7 | 1.0 | 7.4 | 293 | 2.8 | 1.0 | 8.0 | 289 | 1.9 |
| Mali | 0.6 | 0.1 | 2.2 | 345 | 0.7 | 0.2 | 2.2 | 362 | 0.5 | 0.2 | 1.6 | 357 | 2.2 | 1.0 | 4.6 | 303 | 4.1 | 2.4 | 7.2 | 296 | 1.5 |
| Mauritania | 2.5 | 1.0 | 6.2 | 198 | 3.7 | 1.4 | 9.5 | 220 | 5.7 | 3.1 | 10.5 | 190 | 18.7 | 10.9 | 30.1 | 147 | 30.9 | 21.7 | 41.9 | 160 | 10.7 |
| Mexico | 40.4 | 29.9 | 51.7 | 189 | 49.6 | 38.9 | 60.3 | 203 | 41.0 | 23.7 | 60.8 | 134 | 63.7 | 47.9 | 77.0 | 93 | 66.3 | 45.9 | 82.0 | 47 | 48.8 |
| Moldova | 4.6 | 1.1 | 17.4 | 21 | 19.5 | 9.5 | 36.0 | 38 | 27.7 | 10.4 | 55.9 | 27 | 24.3 | 12.4 | 42.3 | 34 | 26.6 | 16.7 | 39.6 | 56 | 21.2 |
| Mongolia | 3.3 | 1.4 | 7.8 | 146 | 7.7 | 4.3 | 13.4 | 150 | 18.4 | 11.8 | 27.7 | 129 | 28.4 | 20.0 | 38.7 | 108 | 32.8 | 24.0 | 43.0 | 111 | 17.4 |
| Myanmar | 2.0 | 0.5 | 7.8 | 119 | 10.9 | 5.3 | 21.3 | 101 | 4.1 | 1.2 | 12.7 | 87 | 3.9 | 1.3 | 11.4 | 87 | 11.7 | 4.9 | 25.2 | 74 | 6.2 |
| Namibia | 6.8 | 3.1 | 14.0 | 122 | 6.8 | 3.1 | 14.4 | 124 | 11.0 | 5.3 | 21.4 | 116 | 29.5 | 19.9 | 41.3 | 97 | 35.4 | 24.1 | 48.5 | 66 | 15.6 |
| Nepal | 1.0 | 0.1 | 6.5 | 131 | 3.4 | 0.9 | 12.4 | 88 | 0.0 | - | - | 98 | 5.4 | 2.0 | 13.5 | 82 | 15.7 | 7.0 | 31.6 | 68 | 4.5 |
| Niger | 0.2 | 0.0 | 1.3 | 268 | 0.2 | 0.0 | 1.7 | 241 | 0.0 | - | - | 243 | 0.9 | 0.2 | 5.4 | 264 | 5.7 | 3.1 | 10.0 | 287 | 1.2 |
| Nigeria | 0.8 | 0.4 | 1.7 | 611 | 2.6 | 1.6 | 4.3 | 638 | 5.2 | 3.0 | 8.8 | 525 | 11.5 | 8.0 | 16.2 | 551 | 15.1 | 11.8 | 19.2 | 423 | 6.4 |
| Pakistan | 6.1 | 3.1 | 11.7 | 256 | 6.7 | 3.8 | 11.8 | 220 | 10.3 | 6.2 | 16.7 | 204 | 15.3 | 10.0 | 22.6 | 210 | 23.0 | 16.8 | 30.7 | 227 | 12.2 |
| Panama | 18.8 | 12.7 | 27.0 | 285 | 45.0 | 29.6 | 61.5 | 86 | 80.9 | 62.8 | 91.4 | 67 | 71.4 | 46.4 | 87.8 | 42 | 78.6 | 53.7 | 92.1 | 30 | 55.0 |
| Paraguay | 13.7 | 7.5 | 23.8 | 105 | 23.2 | 13.5 | 36.9 | 92 | 31.8 | 20.1 | 46.3 | 80 | 44.7 | 28.0 | 62.7 | 68 | 44.1 | 25.7 | 64.2 | 49 | 28.9 |
| Peru | 6.6 | 4.3 | 10.0 | 564 | 13.3 | 9.7 | 17.8 | 444 | 22.9 | 17.8 | 28.8 | 326 | 31.1 | 24.3 | 38.9 | 244 | 47.0 | 37.0 | 57.3 | 159 | 20.7 |
| Rwanda | 0.0 | - | - | 152 | 0.0 | - | - | 141 | 1.0 | 0.1 | 6.8 | 124 | 0.0 | - | - | 131 | 2.1 | 0.8 | 5.2 | 155 | 0.6 |
| Senegal | 1.2 | 0.3 | 4.1 | 350 | 1.4 | 0.5 | 3.8 | 306 | 7.5 | 4.2 | 13.0 | 244 | 5.4 | 2.3 | 12.0 | 151 | 16.2 | 9.0 | 27.4 | 91 | 5.3 |
| Serbia | 23.6 | 7.5 | 54.2 | 17 | 54.4 | 29.6 | 77.1 | 23 | 33.6 | 17.6 | 54.4 | 40 | 61.0 | 33.5 | 82.9 | 39 | 26.6 | 14.1 | 44.5 | 50 | 40.9 |
| Sierra Leone | 1.9 | 0.9 | 3.9 | 312 | 1.4 | 0.6 | 3.6 | 287 | 2.9 | 1.5 | 5.5 | 287 | 10.8 | 6.3 | 17.9 | 166 | 34.8 | 25.6 | 45.2 | 118 | 8.9 |
| South Africa | 30.2 | 19.1 | 44.4 | 68 | 23.8 | 15.3 | 35.0 | 86 | 41.1 | 29.6 | 53.6 | 89 | 29.4 | 17.6 | 44.7 | 60 | 31.8 | 17.2 | 51.2 | 43 | 31.4 |
| South Sudan | 5.4 | 2.7 | 10.5 | 156 | 7.0 | 3.6 | 13.4 | 164 | 3.9 | 1.5 | 9.8 | 166 | 9.9 | 6.4 | 15.1 | 195 | 12.2 | 8.2 | 17.8 | 196 | 7.9 |
| State of Palestine | 39.6 | 31.0 | 48.8 | 154 | 45.1 | 35.6 | 55.0 | 128 | 36.1 | 27.9 | 45.3 | 121 | 40.7 | 32.1 | 49.9 | 154 | 38.4 | 29.6 | 48.1 | 108 | 40.2 |
| Sudan | 2.6 | 1.4 | 4.8 | 367 | 6.3 | 3.3 | 11.9 | 435 | 1.8 | 1.0 | 3.5 | 352 | 1.1 | 0.4 | 3.1 | 223 | 6.7 | 3.1 | 13.8 | 166 | 3.6 |
| Suriname | 54.0 | 42.8 | 64.8 | 112 | 55.9 | 40.3 | 70.5 | 79 | 72.0 | 56.5 | 83.6 | 60 | 50.0 | 32.7 | 67.4 | 50 | 66.9 | 44.4 | 83.7 | 34 | 58.9 |
| Tajikistan | 6.4 | 3.1 | 12.7 | 99 | 5.4 | 2.1 | 13.1 | 79 | 10.9 | 6.5 | 17.7 | 112 | 11.4 | 6.6 | 19.0 | 121 | 12.7 | 7.6 | 20.4 | 142 | 9.7 |
| Tanzania | 0.0 | - | - | 258 | 0.1 | 0.0 | 0.6 | 209 | 0.0 | - | - | 179 | 0.3 | 0.0 | 2.5 | 214 | 4.1 | 1.5 | 10.9 | 155 | 0.7 |
| Thailand | 37.7 | 22.7 | 55.4 | 151 | 40.4 | 26.3 | 56.4 | 153 | 41.4 | 26.6 | 57.9 | 136 | 61.0 | 44.0 | 75.6 | 130 | 36.0 | 19.3 | 56.8 | 91 | 44.9 |
| Timor Leste | 2.1 | 0.6 | 7.5 | 126 | 4.0 | 1.7 | 9.1 | 150 | 9.2 | 4.6 | 17.4 | 158 | 12.2 | 7.2 | 20.0 | 160 | 18.3 | 11.1 | 28.5 | 149 | 9.5 |
| Togo | 0.0 | - | - | 199 | 1.2 | 0.3 | 5.1 | 114 | 1.7 | 0.2 | 11.3 | 102 | 1.9 | 0.5 | 7.6 | 97 | 6.0 | 2.5 | 13.6 | 91 | 2.1 |
| Tunisia | 38.2 | 26.4 | 51.5 | 56 | 47.8 | 34.7 | 61.2 | 62 | 49.8 | 37.2 | 62.3 | 65 | 51.1 | 38.2 | 63.8 | 70 | 72.9 | 57.3 | 84.4 | 46 | 51.5 |
| Turkmenistan | 16.4 | 7.9 | 30.9 | 49 | 6.4 | 2.4 | 16.4 | 59 | 12.7 | 6.1 | 24.4 | 70 | 5.6 | 2.4 | 12.6 | 84 | 21.9 | 13.1 | 34.2 | 80 | 12.3 |
| Uganda | 0.0 | 0.0 | 0.3 | 400 | 0.1 | 0.0 | 0.4 | 331 | 0.4 | 0.1 | 2.3 | 286 | 0.7 | 0.1 | 4.5 | 255 | 1.7 | 0.5 | 5.7 | 210 | 0.5 |
| Ukraine | 26.1 | 16.4 | 39.0 | 65 | 29.9 | 17.2 | 46.6 | 76 | 19.0 | 9.2 | 35.2 | 49 | 41.2 | 25.4 | 59.2 | 57 | 32.8 | 16.6 | 54.5 | 60 | 30.5 |
| Vietnam | 13.8 | 6.8 | 26.1 | 89 | 36.3 | 24.7 | 49.7 | 68 | 39.9 | 27.5 | 53.7 | 74 | 51.0 | 37.3 | 64.6 | 68 | 49.9 | 36.8 | 63.0 | 59 | 37.5 |
| Zambia | 0.3 | 0.0 | 1.8 | 288 | 0.0 | - | - | 309 | 0.3 | 0.0 | 2.1 | 251 | 1.6 | 0.6 | 4.2 | 203 | 10.4 | 5.6 | 18.4 | 138 | 1.8 |
| Zimbabwe | 0.6 | 0.1 | 4.0 | 120 | 0.0 | - | - | 117 | 0.0 | - | - | 99 | 4.9 | 2.3 | 10.1 | 161 | 10.4 | 4.5 | 22.0 | 106 | 2.8 |

^*^DHS: Demographic Health Survey; MICS: Multiple Indicator Cluster Survey; ENSANUT: Encuesta Nacional de Salud y Nutrición; ENDES: Encuesta Demográfica y de Salud Familiar; ^†^CAR: Central African Republic; ^‡^CDR: Congo Democratic Republic.

**Supplementary table 6. Percentage of children between 6-23 months who were fed formula at the time of the survey by wealth quintiles. Source: DHS, MICS, ENSANUT, and ENDES, 2010-2018.^a^**

| **Country** | **Formula consumption between 6-23 months** | | | | | | | | | | | | | | | | | | | | |
| --- | --- | --- | --- | --- | --- | --- | --- | --- | --- | --- | --- | --- | --- | --- | --- | --- | --- | --- | --- | --- | --- |
|  | **Wealth quintiles** | | | | | | | | | | | | | | | | | | | | **National prevalence (%)** |
|  | **Poorest** | | | | **Second** | | | | **Third** | | | | **Fourth** | | | | **Wealthiest** | | | |  |
|  | **%** | **95% CI** | | **N** | **%** | **95% CI** | | **N** | **%** | **95% CI** | | **N** | **%** | **95% CI** | | **N** | **%** | **95% CI** | | **N** |  |
| Afghanistan | 6.1 | 4.3 | 8.4 | 1408 | 8.5 | 6.8 | 10.6 | 1801 | 11.2 | 9.0 | 13.8 | 1796 | 10.7 | 8.2 | 13.8 | 1829 | 24.9 | 20.5 | 29.9 | 1242 | 12.4 |
| Albania | 6.4 | 3.1 | 12.8 | 243 | 7.9 | 4.3 | 14.0 | 184 | 14.9 | 8.5 | 24.8 | 144 | 9.8 | 5.5 | 16.9 | 121 | 23.8 | 13.2 | 38.9 | 74 | 12.2 |
| Algeria | 12.3 | 9.4 | 15.9 | 1018 | 13.9 | 11.2 | 17.1 | 937 | 15.3 | 12.4 | 18.7 | 907 | 19.5 | 15.8 | 23.8 | 865 | 23.8 | 20.1 | 27.9 | 671 | 16.6 |
| Angola | 1.4 | 0.7 | 2.9 | 943 | 1.5 | 0.9 | 2.4 | 1159 | 6.3 | 4.5 | 8.8 | 1000 | 8.3 | 5.7 | 12.0 | 532 | 22.2 | 16.6 | 29.1 | 374 | 6.9 |
| Argentina | 16.2 | 12.3 | 21.1 | 672 | 25.0 | 19.4 | 31.6 | 523 | 31.1 | 24.5 | 38.6 | 512 | 27.9 | 22.1 | 34.5 | 476 | 47.4 | 38.8 | 56.2 | 455 | 27.9 |
| Armenia | 1.8 | 0.4 | 7.8 | 95 | 2.8 | 0.9 | 8.9 | 112 | 5.7 | 2.2 | 14.3 | 107 | 8.5 | 3.5 | 19.4 | 96 | 6.2 | 2.3 | 15.5 | 89 | 5.0 |
| Bangladesh | 0.7 | 0.3 | 2.0 | 493 | 2.1 | 1.0 | 4.5 | 434 | 8.7 | 4.8 | 15.1 | 467 | 7.4 | 5.0 | 10.8 | 496 | 16.6 | 12.5 | 21.6 | 446 | 6.9 |
| Belarus | 38.3 | 27.0 | 51.0 | 147 | 33.7 | 25.0 | 43.7 | 199 | 44.0 | 35.3 | 53.1 | 206 | 42.3 | 34.0 | 51.1 | 243 | 44.0 | 37.4 | 50.9 | 320 | 41.3 |
| Belize | 31.4 | 24.2 | 39.7 | 185 | 44.9 | 36.6 | 53.5 | 169 | 52.0 | 41.3 | 62.4 | 136 | 47.3 | 37.0 | 57.9 | 140 | 59.8 | 44.3 | 73.5 | 100 | 45.5 |
| Benin | 3.5 | 2.1 | 5.7 | 829 | 3.9 | 2.4 | 6.2 | 778 | 2.6 | 1.5 | 4.3 | 775 | 4.2 | 2.7 | 6.3 | 770 | 11.9 | 9.3 | 14.9 | 730 | 5.0 |
| Bhutan | 2.1 | 0.9 | 4.5 | 408 | 5.7 | 3.5 | 9.0 | 439 | 4.7 | 2.6 | 8.4 | 394 | 7.0 | 4.4 | 11.0 | 382 | 16.6 | 12.1 | 22.2 | 305 | 7.2 |
| Burkina Faso | 0.7 | 0.2 | 2.1 | 775 | 0.4 | 0.1 | 1.1 | 843 | 0.6 | 0.2 | 1.5 | 923 | 2.1 | 1.2 | 3.7 | 906 | 9.3 | 6.5 | 13.0 | 701 | 2.2 |
| Burundi | 0.0 | - | - | 797 | 0.2 | 0.0 | 1.1 | 807 | 0.4 | 0.1 | 1.5 | 785 | 0.1 | 0.0 | 0.7 | 704 | 0.6 | 0.2 | 1.4 | 765 | 0.2 |
| CAR^†^ | 2.5 | 1.5 | 4.0 | 766 | 3.4 | 2.2 | 5.3 | 818 | 6.6 | 4.8 | 9.0 | 765 | 13.1 | 9.2 | 18.3 | 580 | 15.0 | 10.4 | 21.1 | 337 | 7.5 |
| Cambodia | 4.1 | 2.4 | 6.8 | 501 | 5.6 | 3.7 | 8.5 | 404 | 7.9 | 5.1 | 12.1 | 320 | 13.5 | 9.8 | 18.3 | 371 | 37.9 | 32.6 | 43.5 | 531 | 13.4 |
| Cameroon | 0.0 | - | - | 368 | 0.8 | 0.3 | 2.3 | 489 | 4.1 | 2.4 | 7.1 | 482 | 6.8 | 4.6 | 10.1 | 432 | 25.6 | 19.1 | 33.5 | 357 | 6.3 |
| Chad | 1.0 | 0.5 | 2.0 | 867 | 1.1 | 0.4 | 3.0 | 938 | 1.1 | 0.6 | 2.0 | 940 | 1.6 | 0.9 | 2.8 | 916 | 19.3 | 16.1 | 22.9 | 742 | 4.1 |
| Colombia | 25.8 | 22.8 | 29.0 | 1840 | 39.0 | 35.5 | 42.6 | 1339 | 44.6 | 40.6 | 48.7 | 920 | 51.6 | 46.7 | 56.5 | 572 | 66.1 | 60.4 | 71.4 | 352 | 42.5 |
| Comoros | 6.0 | 3.4 | 10.4 | 218 | 13.1 | 8.5 | 19.7 | 176 | 16.7 | 11.3 | 24.0 | 187 | 15.2 | 10.0 | 22.3 | 154 | 36.2 | 28.7 | 44.5 | 134 | 16.6 |
| Congo Brazzaville | 2.3 | 1.5 | 3.5 | 1220 | 10.0 | 6.8 | 14.4 | 729 | 13.1 | 9.4 | 18.1 | 374 | 18.4 | 13.3 | 24.9 | 243 | 24.7 | 18.0 | 32.9 | 199 | 12.7 |
| CDR^‡^ | 0.0 | 0.0 | 0.2 | 1324 | 0.2 | 0.0 | 0.8 | 1123 | 1.1 | 0.6 | 2.1 | 1031 | 3.2 | 1.5 | 6.4 | 872 | 10.8 | 8.1 | 14.2 | 641 | 2.7 |
| Costa Rica | 43.3 | 30.4 | 57.3 | 222 | 35.9 | 24.4 | 49.2 | 180 | 46.0 | 30.9 | 61.9 | 124 | 60.4 | 45.8 | 73.4 | 99 | 51.3 | 28.7 | 73.3 | 63 | 45.1 |
| Cote d’Ivoire | 1.0 | 0.5 | 2.0 | 783 | 2.4 | 1.2 | 4.6 | 681 | 2.5 | 1.4 | 4.4 | 565 | 7.2 | 4.7 | 10.8 | 384 | 14.8 | 9.6 | 22.2 | 255 | 4.8 |
| Dominican Republic | 9.2 | 7.0 | 12.0 | 2055 | 12.7 | 10.2 | 15.8 | 1383 | 10.1 | 7.8 | 13.0 | 1170 | 10.8 | 8.2 | 14.2 | 895 | 11.3 | 8.6 | 14.8 | 732 | 10.7 |
| Ecuador | 11.4 | 7.6 | 16.7 | 964 | 24.2 | 18.3 | 31.3 | 787 | 25.2 | 19.5 | 31.8 | 616 | 36.5 | 29.0 | 44.6 | 485 | 48.3 | 38.9 | 57.7 | 341 | 27.5 |
| Egypt | 3.8 | 2.6 | 5.5 | 824 | 4.4 | 3.2 | 6.2 | 897 | 3.1 | 2.1 | 4.4 | 985 | 4.5 | 3.2 | 6.4 | 1056 | 8.0 | 6.1 | 10.3 | 1072 | 4.7 |
| El Salvador | 8.9 | 5.8 | 13.3 | 535 | 9.6 | 6.6 | 13.7 | 481 | 11.6 | 8.3 | 15.8 | 456 | 21.1 | 15.9 | 27.3 | 434 | 31.5 | 25.3 | 38.5 | 360 | 15.3 |
| Eswatini | 12.4 | 8.4 | 17.9 | 216 | 11.6 | 7.3 | 17.9 | 194 | 20.2 | 13.9 | 28.4 | 174 | 22.1 | 14.1 | 32.8 | 105 | 41.6 | 30.4 | 53.7 | 100 | 20.3 |
| Ethiopia | 0.7 | 0.1 | 3.5 | 950 | 1.0 | 0.3 | 3.7 | 474 | 0.7 | 0.2 | 2.8 | 423 | 1.2 | 0.4 | 3.6 | 347 | 5.4 | 3.1 | 9.2 | 627 | 1.6 |
| Gabon | 12.6 | 9.5 | 16.4 | 806 | 24.3 | 18.8 | 30.8 | 373 | 41.4 | 32.0 | 51.3 | 244 | 40.3 | 30.3 | 51.0 | 175 | 63.4 | 51.8 | 73.6 | 121 | 34.5 |
| Gambia | 2.4 | 1.2 | 4.5 | 605 | 1.3 | 0.6 | 3.0 | 653 | 3.2 | 1.2 | 8.3 | 532 | 7.7 | 4.0 | 14.4 | 352 | 9.9 | 6.0 | 16.0 | 281 | 4.6 |
| Ghana | 0.4 | 0.1 | 1.4 | 542 | 1.3 | 0.5 | 3.1 | 369 | 2.9 | 1.3 | 6.3 | 287 | 5.7 | 3.2 | 9.9 | 256 | 15.1 | 10.8 | 20.8 | 202 | 4.7 |
| Guatemala | 1.3 | 0.7 | 2.5 | 943 | 2.9 | 1.9 | 4.3 | 804 | 5.7 | 4.1 | 7.8 | 720 | 13.3 | 10.1 | 17.3 | 610 | 24.3 | 19.6 | 29.7 | 432 | 7.8 |
| Guinea | 2.0 | 0.9 | 4.4 | 469 | 3.2 | 1.8 | 5.6 | 469 | 6.4 | 3.3 | 12.3 | 450 | 10.3 | 7.3 | 14.3 | 417 | 25.2 | 20.2 | 30.9 | 317 | 8.8 |
| Guinea Bissau | 7.5 | 5.6 | 10.1 | 688 | 5.4 | 3.5 | 8.2 | 530 | 7.2 | 4.9 | 10.3 | 524 | 17.2 | 11.9 | 24.3 | 348 | 25.7 | 17.5 | 36.0 | 178 | 11.5 |
| Guyana | 14.2 | 10.3 | 19.2 | 373 | 21.5 | 13.7 | 32.0 | 201 | 23.5 | 16.7 | 32.1 | 163 | 29.7 | 21.0 | 40.3 | 150 | 41.7 | 30.8 | 53.4 | 147 | 24.1 |
| Haiti | 4.1 | 2.4 | 6.7 | 543 | 7.9 | 5.5 | 11.3 | 380 | 13.6 | 9.7 | 18.8 | 321 | 22.2 | 16.3 | 29.5 | 231 | 46.0 | 38.2 | 54.0 | 177 | 15.6 |
| Honduras | 0.9 | 0.5 | 1.6 | 1069 | 2.4 | 1.4 | 4.2 | 763 | 3.9 | 2.4 | 6.4 | 589 | 4.7 | 3.0 | 7.2 | 469 | 14.5 | 10.3 | 20.2 | 347 | 4.5 |
| India | 5.9 | 5.4 | 6.4 | 18220 | 8.1 | 7.5 | 8.8 | 16608 | 11.8 | 11.0 | 12.8 | 14567 | 14.8 | 13.9 | 15.9 | 12234 | 14.9 | 13.7 | 16.1 | 10116 | 10.6 |
| Indonesia | 16.6 | 13.9 | 19.6 | 1324 | 26.6 | 23.3 | 30.2 | 990 | 34.3 | 30.7 | 38.0 | 912 | 46.8 | 42.9 | 50.8 | 927 | 59.1 | 55.2 | 62.9 | 880 | 36.9 |
| Iraq | 32.4 | 28.3 | 36.9 | 1297 | 42.5 | 36.6 | 48.5 | 1109 | 51.0 | 46.2 | 55.7 | 942 | 52.0 | 47.0 | 56.9 | 790 | 53.8 | 41.9 | 65.2 | 648 | 45.8 |
| Jamaica | 47.3 | 36.9 | 57.9 | 129 | 62.6 | 50.6 | 73.2 | 100 | 67.2 | 55.7 | 77.0 | 88 | 55.2 | 42.1 | 67.6 | 85 | 60.5 | 44.2 | 74.8 | 74 | 58.2 |
| Jordan | 35.9 | 31.6 | 40.5 | 991 | 44.3 | 38.6 | 50.2 | 720 | 52.0 | 45.5 | 58.4 | 565 | 53.2 | 45.0 | 61.2 | 293 | 61.5 | 48.5 | 73.1 | 111 | 46.8 |
| Kazakhstan | 6.9 | 4.4 | 10.7 | 306 | 13.7 | 9.5 | 19.3 | 312 | 13.1 | 9.5 | 17.9 | 386 | 15.2 | 11.2 | 20.4 | 338 | 15.4 | 11.5 | 20.4 | 290 | 12.8 |
| Kenya | 2.1 | 1.2 | 3.5 | 990 | 2.3 | 1.3 | 4.0 | 584 | 5.3 | 3.0 | 9.3 | 442 | 7.0 | 4.3 | 11.4 | 428 | 12.1 | 8.1 | 17.7 | 365 | 5.6 |
| Kosovo | 0.0 | - | - | 105 | 3.4 | 1.2 | 9.2 | 101 | 6.8 | 3.3 | 13.5 | 103 | 9.5 | 5.0 | 17.2 | 99 | 13.7 | 7.7 | 23.4 | 82 | 6.4 |
| Kyrgyzstan | 11.6 | 7.1 | 18.5 | 222 | 11.5 | 6.8 | 18.8 | 170 | 14.3 | 8.3 | 23.5 | 190 | 21.9 | 16.5 | 28.4 | 245 | 31.4 | 24.3 | 39.6 | 165 | 17.0 |
| Lao | 3.1 | 2.0 | 4.7 | 913 | 8.6 | 6.6 | 11.2 | 843 | 20.3 | 16.7 | 24.4 | 645 | 27.6 | 23.3 | 32.3 | 549 | 48.6 | 43.7 | 53.4 | 478 | 19.7 |
| Lesotho | 1.2 | 0.4 | 3.9 | 264 | 6.5 | 3.4 | 12.3 | 193 | 8.0 | 4.8 | 13.0 | 203 | 22.5 | 15.5 | 31.4 | 157 | 36.3 | 25.5 | 48.7 | 131 | 13.3 |
| Liberia | 0.5 | 0.2 | 1.5 | 779 | 2.7 | 1.1 | 6.5 | 562 | 2.8 | 0.5 | 14.8 | 430 | 7.0 | 3.5 | 13.2 | 240 | 21.0 | 13.7 | 30.7 | 144 | 5.7 |
| Malawi | 1.1 | 0.6 | 2.0 | 1120 | 1.4 | 0.8 | 2.4 | 1053 | 1.8 | 1.0 | 3.0 | 920 | 1.8 | 1.1 | 3.0 | 852 | 8.6 | 5.4 | 13.2 | 802 | 2.6 |
| Mali | 1.7 | 0.8 | 3.5 | 997 | 3.1 | 2.0 | 4.7 | 976 | 2.5 | 1.6 | 3.9 | 940 | 4.7 | 3.1 | 7.0 | 1036 | 15.5 | 12.6 | 19.0 | 916 | 5.2 |
| Mauritania | 2.2 | 1.1 | 4.2 | 603 | 3.3 | 1.9 | 5.5 | 668 | 5.8 | 4.0 | 8.4 | 711 | 12.3 | 9.1 | 16.4 | 631 | 31.8 | 25.3 | 39.1 | 571 | 10.6 |
| Mexico | 23.7 | 19.0 | 29.2 | 724 | 35.3 | 27.7 | 43.7 | 573 | 49.0 | 40.5 | 57.7 | 453 | 45.4 | 35.9 | 55.2 | 353 | 67.3 | 55.5 | 77.2 | 208 | 40.4 |
| Moldova | 15.3 | 8.0 | 27.0 | 73 | 13.1 | 7.5 | 21.9 | 95 | 20.7 | 13.3 | 30.7 | 94 | 33.2 | 24.2 | 43.6 | 111 | 37.0 | 30.0 | 44.7 | 218 | 24.8 |
| Mongolia | 1.5 | 0.7 | 3.3 | 436 | 3.4 | 1.7 | 6.6 | 332 | 7.6 | 5.2 | 11.1 | 340 | 10.9 | 7.6 | 15.3 | 331 | 10.5 | 7.6 | 14.4 | 363 | 6.8 |
| Myanmar | 3.3 | 1.6 | 6.7 | 376 | 4.0 | 2.1 | 7.8 | 299 | 2.3 | 0.8 | 6.5 | 253 | 6.7 | 3.9 | 11.3 | 241 | 10.9 | 6.6 | 17.7 | 170 | 5.1 |
| Namibia | 3.1 | 1.6 | 6.0 | 280 | 5.3 | 2.8 | 10.0 | 306 | 9.2 | 6.1 | 13.6 | 295 | 17.1 | 12.0 | 23.9 | 253 | 32.8 | 25.1 | 41.7 | 168 | 11.7 |
| Nepal | 0.8 | 0.3 | 2.2 | 355 | 0.8 | 0.2 | 2.7 | 321 | 3.0 | 1.6 | 5.8 | 316 | 2.7 | 1.3 | 5.4 | 292 | 6.8 | 3.5 | 12.8 | 179 | 2.5 |
| Niger | 0.5 | 0.1 | 1.6 | 520 | 0.2 | 0.0 | 1.2 | 570 | 0.9 | 0.4 | 2.5 | 603 | 0.5 | 0.2 | 1.2 | 636 | 6.1 | 4.6 | 8.0 | 930 | 1.6 |
| Nigeria | 2.4 | 1.8 | 3.4 | 1789 | 3.0 | 2.2 | 4.1 | 1822 | 4.2 | 3.2 | 5.5 | 1578 | 7.6 | 6.3 | 9.3 | 1519 | 17.6 | 15.3 | 20.1 | 1511 | 6.6 |
| Pakistan | 3.3 | 2.0 | 5.6 | 562 | 4.5 | 2.7 | 7.3 | 510 | 7.0 | 4.7 | 10.2 | 510 | 7.4 | 4.9 | 11.1 | 468 | 11.2 | 7.8 | 15.8 | 515 | 6.6 |
| Panama | 19.1 | 14.1 | 25.4 | 998 | 36.8 | 28.4 | 46.1 | 359 | 51.9 | 41.5 | 62.1 | 250 | 61.4 | 49.6 | 72.0 | 221 | 58.4 | 42.0 | 73.1 | 97 | 40.9 |
| Paraguay | 8.0 | 5.5 | 11.5 | 412 | 8.5 | 5.6 | 12.8 | 319 | 12.4 | 8.5 | 17.6 | 272 | 12.4 | 8.1 | 18.6 | 262 | 23.3 | 16.7 | 31.4 | 178 | 11.7 |
| Peru | 6.3 | 5.0 | 7.8 | 1908 | 10.8 | 9.2 | 12.7 | 1699 | 20.1 | 17.5 | 23.0 | 1282 | 30.2 | 26.6 | 34.0 | 939 | 40.4 | 35.7 | 45.2 | 706 | 19.4 |
| Rwanda | 0.7 | 0.3 | 1.7 | 593 | 0.4 | 0.1 | 1.4 | 475 | 0.7 | 0.2 | 2.2 | 433 | 1.6 | 0.7 | 3.3 | 402 | 3.4 | 2.0 | 5.6 | 450 | 1.2 |
| Senegal | 0.6 | 0.2 | 1.8 | 1072 | 1.3 | 0.7 | 2.4 | 871 | 1.2 | 0.6 | 2.4 | 768 | 5.3 | 2.5 | 10.8 | 469 | 16.1 | 11.1 | 22.7 | 309 | 4.2 |
| Serbia | 4.1 | 1.8 | 9.0 | 123 | 7.3 | 2.7 | 18.4 | 128 | 6.3 | 3.6 | 10.7 | 164 | 10.7 | 6.6 | 17.0 | 175 | 14.5 | 10.1 | 20.5 | 205 | 8.9 |
| Sierra Leone | 2.0 | 1.3 | 3.2 | 1028 | 2.1 | 1.3 | 3.3 | 836 | 5.0 | 3.3 | 7.4 | 732 | 18.5 | 14.8 | 23.0 | 460 | 33.5 | 27.7 | 39.8 | 355 | 10.4 |
| South Africa | 28.2 | 21.4 | 36.2 | 237 | 33.5 | 26.8 | 41.0 | 213 | 34.6 | 27.0 | 43.0 | 180 | 52.9 | 42.0 | 63.5 | 164 | 64.0 | 50.8 | 75.3 | 83 | 40.2 |
| South Sudan | 5.4 | 3.6 | 7.9 | 482 | 5.6 | 3.7 | 8.4 | 490 | 6.4 | 4.3 | 9.2 | 491 | 11.8 | 8.9 | 15.4 | 560 | 22.7 | 18.5 | 27.5 | 530 | 10.6 |
| State of Palestine | 11.7 | 9.1 | 15.0 | 557 | 17.2 | 13.5 | 21.5 | 422 | 27.6 | 23.5 | 32.1 | 500 | 28.4 | 24.5 | 32.6 | 486 | 39.0 | 34.0 | 44.2 | 361 | 23.4 |
| Sudan | 2.5 | 1.6 | 3.8 | 854 | 5.0 | 3.7 | 6.8 | 1087 | 2.2 | 1.4 | 3.5 | 914 | 3.7 | 2.2 | 6.0 | 689 | 4.1 | 2.2 | 7.5 | 520 | 3.5 |
| Suriname | 51.7 | 43.4 | 59.9 | 326 | 65.8 | 55.6 | 74.7 | 273 | 72.1 | 64.3 | 78.8 | 229 | 72.5 | 63.2 | 80.2 | 212 | 72.0 | 59.5 | 81.7 | 142 | 64.9 |
| Tajikistan | 8.1 | 5.3 | 12.3 | 295 | 13.5 | 9.9 | 18.3 | 309 | 10.5 | 7.5 | 14.6 | 351 | 9.6 | 6.6 | 13.7 | 356 | 17.8 | 14.2 | 22.2 | 411 | 11.7 |
| Tanzania | 0.5 | 0.2 | 1.5 | 682 | 0.4 | 0.1 | 1.6 | 609 | 0.7 | 0.2 | 2.3 | 572 | 0.7 | 0.2 | 2.6 | 645 | 3.7 | 2.1 | 6.3 | 511 | 1.1 |
| Thailand | 38.9 | 31.2 | 47.1 | 690 | 47.7 | 40.1 | 55.4 | 725 | 54.4 | 45.9 | 62.7 | 688 | 64.4 | 55.2 | 72.6 | 702 | 66.6 | 53.4 | 77.6 | 417 | 53.8 |
| Timor Leste | 3.5 | 1.9 | 6.5 | 381 | 7.4 | 5.1 | 10.7 | 413 | 8.6 | 6.1 | 12.0 | 414 | 14.8 | 11.5 | 18.9 | 439 | 27.0 | 20.9 | 34.3 | 303 | 11.9 |
| Togo | 0.2 | 0.0 | 1.1 | 590 | 0.2 | 0.0 | 1.1 | 425 | 0.4 | 0.1 | 1.6 | 419 | 2.1 | 1.0 | 4.4 | 331 | 9.6 | 6.7 | 13.6 | 305 | 2.3 |
| Tunisia | 4.5 | 2.1 | 9.3 | 199 | 14.5 | 10.2 | 20.3 | 224 | 13.3 | 9.0 | 19.4 | 184 | 13.4 | 9.2 | 19.0 | 204 | 20.7 | 14.9 | 28.0 | 135 | 13.1 |
| Turkmenistan | 5.0 | 2.8 | 8.7 | 198 | 8.6 | 5.0 | 14.3 | 215 | 8.6 | 5.3 | 13.8 | 251 | 6.8 | 4.1 | 11.3 | 258 | 20.9 | 15.9 | 26.9 | 247 | 9.5 |
| Uganda | 0.2 | 0.0 | 0.7 | 1126 | 0.3 | 0.1 | 0.8 | 913 | 0.3 | 0.1 | 1.1 | 781 | 0.0 | - | - | 673 | 1.0 | 0.5 | 2.2 | 665 | 0.4 |
| Ukraine | 25.5 | 18.5 | 33.9 | 268 | 31.8 | 23.4 | 41.6 | 301 | 43.6 | 32.7 | 55.2 | 197 | 48.7 | 37.7 | 59.8 | 240 | 44.2 | 34.5 | 54.4 | 250 | 38.8 |
| Vietnam | 17.1 | 12.2 | 23.5 | 264 | 31.2 | 24.6 | 38.7 | 200 | 30.9 | 24.6 | 38.1 | 194 | 59.4 | 50.5 | 67.8 | 231 | 67.2 | 59.3 | 74.2 | 229 | 41.8 |
| Zambia | 0.1 | 0.0 | 0.6 | 920 | 0.7 | 0.2 | 2.0 | 870 | 0.2 | 0.0 | 0.9 | 847 | 2.3 | 1.1 | 4.5 | 617 | 6.4 | 4.1 | 9.9 | 468 | 1.5 |
| Zimbabwe | 0.3 | 0.1 | 1.4 | 369 | 0.4 | 0.1 | 1.5 | 301 | 0.0 | - | - | 268 | 1.8 | 0.7 | 4.1 | 395 | 7.3 | 4.6 | 11.5 | 295 | 1.6 |

^*^DHS: Demographic Health Survey; MICS: Multiple Indicator Cluster Survey; ENSANUT: Encuesta Nacional de Salud y Nutrición; ENDES: Encuesta Demográfica y de Salud Familiar; ^†^CAR: Central African Republic; ^‡^CDR: Congo Democratic Republic.

**Supplementary table 7. Percentage of children under 6 months who were fed other non-human milk (other than formula) at the time of the survey by wealth quintiles. Source: DHS, MICS, ENSANUT, and ENDES, 2010-2018.^a^**

| **Country** | **Other non-human milk consumption under 6 months (0-5 months)** | | | | | | | | | | | | | | | | | | | | |
| --- | --- | --- | --- | --- | --- | --- | --- | --- | --- | --- | --- | --- | --- | --- | --- | --- | --- | --- | --- | --- | --- |
|  | **Wealth quintiles** | | | | | | | | | | | | | | | | | | | | **National prevalence (%)** |
|  | **Poorest** | | | | **Second** | | | | **Third** | | | | **Fourth** | | | | **Wealthiest** | | | |  |
|  | **%** | **95% CI** | | **N** | **%** | **95% CI** | | **N** | **%** | **95% CI** | | **N** | **%** | **95% CI** | | **N** | **%** | **95% CI** | | **N** |  |
| Afghanistan | 40.6 | 34.0 | 47.7 | 528 | 34.7 | 27.4 | 42.7 | 754 | 31.9 | 26.4 | 38.0 | 759 | 28.0 | 20.8 | 36.5 | 710 | 28.0 | 20.0 | 37.9 | 452 | 32.5 |
| Albania | 17.3 | 9.6 | 29.1 | 84 | 19.8 | 8.6 | 39.4 | 60 | 17.6 | 7.0 | 37.7 | 55 | 21.0 | 10.4 | 37.7 | 59 | 19.0 | 5.9 | 47.0 | 27 | 19.0 |
| Algeria | 39.9 | 33.0 | 47.3 | 399 | 34.7 | 28.7 | 41.3 | 344 | 41.0 | 34.3 | 48.1 | 316 | 45.4 | 38.2 | 52.8 | 312 | 37.8 | 30.9 | 45.2 | 253 | 39.6 |
| Angola | 2.4 | 1.3 | 4.3 | 413 | 0.9 | 0.3 | 3.1 | 448 | 3.9 | 1.3 | 11.2 | 418 | 7.8 | 3.2 | 17.7 | 211 | 6.7 | 2.9 | 14.9 | 130 | 4.0 |
| Argentina | 16.6 | 10.9 | 24.6 | 214 | 19.0 | 9.9 | 33.4 | 143 | 2.1 | 0.9 | 4.8 | 159 | 11.4 | 5.1 | 23.6 | 149 | 1.6 | 0.5 | 4.7 | 123 | 11.0 |
| Armenia | 17.2 | 6.7 | 37.5 | 28 | 16.8 | 7.5 | 33.7 | 37 | 13.6 | 5.7 | 28.8 | 39 | 7.0 | 2.0 | 21.9 | 35 | 23.4 | 12.0 | 40.8 | 38 | 16.1 |
| Bangladesh | 11.6 | 5.4 | 23.2 | 122 | 9.9 | 4.7 | 19.4 | 130 | 6.6 | 2.8 | 14.9 | 121 | 4.9 | 2.2 | 10.5 | 120 | 8.5 | 3.1 | 21.5 | 139 | 8.4 |
| Belarus | 8.1 | 1.1 | 40.7 | 36 | 5.3 | 1.8 | 14.3 | 51 | 0.0 | - | - | 41 | 2.3 | 0.3 | 15.1 | 52 | 4.1 | 1.4 | 11.5 | 67 | 3.8 |
| Belize | 11.8 | 4.1 | 29.5 | 40 | 15.7 | 6.2 | 34.4 | 37 | 9.8 | 3.9 | 22.4 | 34 | 4.4 | 1.3 | 13.8 | 33 | 13.7 | 4.3 | 36.2 | 21 | 11.1 |
| Benin | 3.1 | 1.5 | 6.3 | 311 | 2.9 | 1.5 | 5.6 | 285 | 3.0 | 1.4 | 6.5 | 281 | 2.6 | 1.1 | 6.0 | 251 | 3.6 | 1.9 | 6.8 | 253 | 3.0 |
| Bhutan | 9.6 | 4.2 | 20.4 | 123 | 4.8 | 1.6 | 13.1 | 115 | 7.2 | 3.4 | 14.7 | 149 | 4.4 | 2.0 | 9.8 | 119 | 2.3 | 0.4 | 11.8 | 102 | 5.6 |
| Burkina Faso | 2.2 | 0.8 | 6.0 | 275 | 1.5 | 0.6 | 3.7 | 324 | 0.9 | 0.2 | 3.4 | 329 | 0.8 | 0.2 | 3.3 | 318 | 1.4 | 0.4 | 5.6 | 208 | 1.3 |
| Burundi | 0.0 | - | - | 233 | 0.4 | 0.1 | 3.0 | 241 | 0.6 | 0.1 | 4.0 | 222 | 0.1 | 0.0 | 0.9 | 236 | 6.5 | 3.6 | 11.5 | 304 | 1.4 |
| CAR^†^ | 0.1 | 0.0 | 0.8 | 277 | 0.1 | 0.0 | 0.5 | 314 | 0.8 | 0.2 | 2.8 | 325 | 2.9 | 1.1 | 7.3 | 233 | 7.2 | 3.2 | 15.4 | 134 | 1.9 |
| Cambodia | 2.8 | 0.8 | 9.8 | 156 | 1.9 | 0.5 | 6.5 | 121 | 4.1 | 1.7 | 9.6 | 119 | 2.5 | 0.9 | 7.2 | 134 | 7.5 | 3.4 | 15.9 | 158 | 3.7 |
| Cameroon | 4.6 | 1.4 | 13.6 | 117 | 2.5 | 0.6 | 9.9 | 153 | 3.8 | 1.5 | 8.9 | 174 | 3.1 | 1.5 | 6.6 | 147 | 15.4 | 8.6 | 26.0 | 112 | 5.3 |
| Chad | 6.2 | 3.9 | 9.7 | 355 | 11.0 | 7.5 | 15.8 | 356 | 10.9 | 7.7 | 15.1 | 375 | 15.4 | 11.6 | 20.3 | 405 | 18.7 | 13.7 | 25.0 | 332 | 12.2 |
| Colombia | 12.7 | 9.1 | 17.5 | 567 | 7.9 | 5.2 | 11.7 | 413 | 3.0 | 1.6 | 5.4 | 282 | 3.7 | 1.5 | 9.1 | 171 | 0.0 | - | - | 82 | 6.4 |
| Comoros | 4.1 | 1.4 | 11.1 | 89 | 23.3 | 12.1 | 40.2 | 66 | 12.3 | 5.4 | 25.6 | 61 | 13.2 | 6.5 | 24.9 | 54 | 27.4 | 17.5 | 40.1 | 57 | 15.2 |
| Congo Brazzaville | 5.2 | 3.3 | 8.0 | 432 | 12.5 | 6.7 | 22.2 | 219 | 14.8 | 7.3 | 27.5 | 112 | 27.3 | 16.6 | 41.4 | 78 | 16.7 | 8.3 | 30.7 | 58 | 14.7 |
| CDR^‡^ | 0.8 | 0.3 | 2.7 | 535 | 0.4 | 0.1 | 1.3 | 434 | 3.0 | 0.7 | 11.5 | 369 | 2.5 | 1.0 | 6.2 | 333 | 7.6 | 4.0 | 14.2 | 263 | 2.7 |
| Costa Rica | 17.9 | 7.7 | 36.4 | 90 | 18.6 | 3.6 | 57.9 | 48 | 1.5 | 0.2 | 10.1 | 32 | 4.8 | 0.6 | 28.3 | 24 | 15.3 | 2.1 | 60.8 | 22 | 13.3 |
| Cote d’Ivoire | 2.1 | 0.8 | 5.0 | 316 | 1.9 | 0.5 | 6.9 | 253 | 6.1 | 3.0 | 12.0 | 219 | 12.3 | 5.2 | 26.3 | 119 | 13.7 | 6.3 | 27.4 | 74 | 6.0 |
| Dominican Republic | 55.2 | 49.3 | 61.0 | 552 | 62.8 | 54.2 | 70.7 | 377 | 56.6 | 45.6 | 66.9 | 293 | 66.9 | 58.5 | 74.4 | 253 | 61.2 | 50.5 | 71.0 | 181 | 60.1 |
| Ecuador | 4.0 | 1.7 | 9.2 | 317 | 0.1 | 0.0 | 0.5 | 224 | 3.2 | 1.1 | 9.2 | 170 | 1.1 | 0.2 | 4.7 | 117 | 0.9 | 0.2 | 3.6 | 92 | 1.9 |
| Egypt | 8.2 | 4.8 | 13.9 | 299 | 2.7 | 1.1 | 6.3 | 263 | 8.6 | 5.6 | 12.9 | 324 | 5.5 | 3.2 | 9.3 | 314 | 4.8 | 2.5 | 9.0 | 287 | 6.2 |
| El Salvador | 6.3 | 3.2 | 12.2 | 144 | 7.7 | 3.5 | 16.3 | 104 | 5.0 | 1.5 | 15.3 | 98 | 3.3 | 1.4 | 7.5 | 86 | 5.7 | 1.1 | 24.2 | 83 | 5.7 |
| Eswatini | 4.5 | 1.0 | 17.8 | 65 | 0.6 | 0.1 | 4.6 | 59 | 1.1 | 0.1 | 7.3 | 56 | 0.0 | - | - | 30 | 0.0 | - | - | 25 | 1.5 |
| Ethiopia | 6.8 | 4.3 | 10.5 | 418 | 2.4 | 0.9 | 6.1 | 174 | 8.9 | 4.1 | 18.4 | 129 | 5.1 | 2.3 | 11.3 | 145 | 6.1 | 3.3 | 11.0 | 226 | 5.7 |
| Gabon | 6.2 | 3.7 | 10.0 | 300 | 10.0 | 3.7 | 24.2 | 137 | 21.4 | 10.1 | 40.0 | 88 | 7.0 | 1.9 | 22.1 | 60 | 7.3 | 2.6 | 19.2 | 46 | 10.8 |
| Gambia | 1.6 | 0.5 | 5.2 | 230 | 0.2 | 0.0 | 1.1 | 235 | 1.6 | 0.5 | 4.7 | 192 | 3.1 | 0.9 | 10.2 | 177 | 4.8 | 1.5 | 14.3 | 117 | 2.1 |
| Ghana | 2.0 | 0.7 | 5.5 | 210 | 5.3 | 2.3 | 12.1 | 121 | 8.2 | 3.5 | 18.0 | 112 | 10.9 | 5.5 | 20.3 | 91 | 12.9 | 5.4 | 27.8 | 72 | 7.4 |
| Guatemala | 0.4 | 0.1 | 2.5 | 318 | 0.3 | 0.0 | 2.1 | 287 | 2.3 | 0.9 | 5.8 | 222 | 5.1 | 2.4 | 10.5 | 206 | 1.4 | 0.3 | 5.4 | 142 | 1.7 |
| Guinea | 6.5 | 3.7 | 11.3 | 168 | 3.7 | 1.7 | 8.0 | 162 | 12.4 | 5.9 | 23.9 | 139 | 6.4 | 3.4 | 11.7 | 133 | 6.4 | 2.9 | 13.5 | 84 | 7.0 |
| Guinea Bissau | 3.8 | 1.9 | 7.7 | 260 | 3.7 | 1.6 | 8.2 | 209 | 3.9 | 1.6 | 9.5 | 198 | 12.1 | 5.9 | 23.1 | 94 | 20.8 | 12.5 | 32.6 | 69 | 7.5 |
| Guyana | 33.1 | 22.2 | 46.2 | 114 | 47.1 | 29.3 | 65.7 | 59 | 44.0 | 27.1 | 62.4 | 50 | 50.6 | 29.0 | 71.9 | 33 | 59.6 | 39.4 | 77.0 | 34 | 44.4 |
| Haiti | 20.0 | 13.8 | 28.0 | 200 | 13.0 | 8.2 | 20.2 | 189 | 14.7 | 8.6 | 24.0 | 141 | 21.7 | 14.6 | 30.9 | 105 | 20.6 | 11.4 | 34.5 | 65 | 17.3 |
| Honduras | 13.9 | 10.0 | 18.9 | 341 | 23.2 | 17.9 | 29.6 | 270 | 22.5 | 16.0 | 30.7 | 185 | 21.4 | 14.9 | 29.8 | 178 | 12.9 | 7.0 | 22.6 | 110 | 19.0 |
| India | 13.0 | 12.0 | 14.2 | 5829 | 13.7 | 12.6 | 15.0 | 5494 | 15.5 | 14.1 | 17.0 | 4508 | 17.8 | 15.9 | 19.9 | 3660 | 19.9 | 17.8 | 22.2 | 3135 | 15.5 |
| Indonesia | 1.0 | 0.3 | 2.9 | 469 | 1.2 | 0.4 | 3.5 | 346 | 0.6 | 0.1 | 4.0 | 309 | 1.0 | 0.3 | 2.9 | 290 | 0.0 | 0.0 | 0.3 | 252 | 0.8 |
| Iraq | 3.1 | 1.8 | 5.2 | 439 | 3.6 | 1.4 | 8.9 | 387 | 2.9 | 1.4 | 5.8 | 352 | 3.2 | 1.4 | 6.9 | 275 | 1.8 | 0.7 | 4.4 | 228 | 3.0 |
| Jamaica | 12.8 | 4.5 | 31.1 | 28 | 2.5 | 0.5 | 10.5 | 39 | 7.4 | 2.7 | 18.6 | 47 | 6.7 | 0.9 | 35.2 | 30 | 4.5 | 1.0 | 17.4 | 23 | 6.4 |
| Jordan | 10.0 | 6.6 | 14.7 | 441 | 6.5 | 3.8 | 11.1 | 312 | 6.5 | 3.6 | 11.5 | 241 | 11.5 | 6.8 | 18.6 | 164 | 12.1 | 5.0 | 26.5 | 60 | 8.9 |
| Kazakhstan | 6.4 | 1.8 | 20.6 | 82 | 6.4 | 2.6 | 14.8 | 90 | 3.2 | 1.2 | 8.2 | 142 | 3.3 | 1.0 | 10.2 | 102 | 1.5 | 0.4 | 5.5 | 92 | 4.2 |
| Kenya | 16.2 | 11.6 | 22.2 | 320 | 20.7 | 13.9 | 29.7 | 167 | 21.6 | 14.1 | 31.5 | 140 | 10.0 | 3.3 | 26.7 | 119 | 9.9 | 5.0 | 18.8 | 110 | 16.0 |
| Kosovo | 22.3 | 10.7 | 40.8 | 38 | 5.8 | 0.8 | 32.0 | 24 | 15.1 | 4.8 | 38.6 | 28 | 11.9 | 3.7 | 32.1 | 25 | 2.8 | 0.4 | 18.5 | 30 | 12.4 |
| Kyrgyzstan | 1.5 | 0.4 | 6.0 | 78 | 3.5 | 1.2 | 10.2 | 72 | 1.8 | 0.2 | 11.8 | 76 | 3.4 | 1.0 | 10.3 | 104 | 1.8 | 0.4 | 7.3 | 60 | 2.5 |
| Lao | 0.6 | 0.2 | 2.1 | 348 | 1.6 | 0.7 | 3.9 | 250 | 0.0 | - | - | 213 | 1.9 | 0.5 | 6.5 | 171 | 4.7 | 2.0 | 10.4 | 152 | 1.6 |
| Lesotho | 2.7 | 0.7 | 9.7 | 82 | 1.7 | 0.2 | 11.3 | 76 | 0.0 | - | - | 67 | 7.3 | 1.8 | 25.3 | 59 | 12.4 | 3.4 | 36.5 | 43 | 4.4 |
| Liberia | 0.5 | 0.1 | 3.4 | 256 | 1.2 | 0.2 | 6.4 | 194 | 6.4 | 2.7 | 14.5 | 148 | 7.5 | 2.4 | 21.5 | 77 | 11.4 | 4.2 | 27.2 | 42 | 4.7 |
| Malawi | 0.7 | 0.2 | 2.2 | 379 | 1.6 | 0.7 | 3.8 | 358 | 0.8 | 0.2 | 3.1 | 317 | 0.9 | 0.2 | 3.7 | 293 | 1.2 | 0.4 | 3.8 | 289 | 1.0 |
| Mali | 11.7 | 8.4 | 16.1 | 345 | 6.3 | 4.2 | 9.4 | 362 | 7.1 | 4.4 | 11.2 | 357 | 6.6 | 4.2 | 10.4 | 303 | 11.8 | 8.4 | 16.4 | 296 | 8.6 |
| Mauritania | 17.4 | 12.5 | 23.7 | 198 | 21.2 | 15.6 | 28.2 | 220 | 23.0 | 16.4 | 31.4 | 190 | 30.0 | 20.9 | 41.0 | 147 | 30.3 | 21.3 | 41.2 | 160 | 23.5 |
| Mexico | 4.4 | 1.3 | 13.6 | 189 | 9.2 | 3.9 | 20.0 | 203 | 11.7 | 3.4 | 33.5 | 134 | 5.8 | 2.7 | 12.0 | 93 | 1.6 | 0.2 | 10.9 | 47 | 7.7 |
| Moldova | 17.4 | 5.2 | 44.8 | 21 | 19.6 | 9.3 | 36.8 | 38 | 9.7 | 2.5 | 31.1 | 27 | 10.2 | 3.2 | 28.1 | 34 | 6.0 | 2.1 | 15.5 | 56 | 12.8 |
| Mongolia | 16.1 | 11.1 | 22.6 | 146 | 9.4 | 5.8 | 15.0 | 150 | 8.5 | 4.5 | 15.4 | 129 | 12.2 | 6.8 | 20.9 | 108 | 7.6 | 4.0 | 14.0 | 111 | 10.7 |
| Myanmar | 0.0 | - | - | 119 | 7.8 | 3.0 | 18.8 | 101 | 1.9 | 0.6 | 6.2 | 87 | 4.2 | 1.2 | 13.2 | 87 | 10.1 | 4.4 | 21.7 | 74 | 4.5 |
| Namibia | 4.9 | 2.0 | 11.4 | 122 | 5.7 | 2.6 | 11.9 | 124 | 13.8 | 7.3 | 24.4 | 116 | 22.5 | 13.8 | 34.6 | 97 | 21.0 | 11.9 | 34.3 | 66 | 12.3 |
| Nepal | 10.7 | 5.9 | 18.8 | 131 | 13.4 | 7.5 | 22.9 | 88 | 4.4 | 1.7 | 10.8 | 98 | 16.9 | 9.9 | 27.4 | 82 | 20.5 | 11.4 | 34.1 | 68 | 12.6 |
| Niger | 2.8 | 1.3 | 5.8 | 268 | 3.4 | 1.0 | 10.5 | 241 | 2.4 | 1.0 | 5.9 | 243 | 4.5 | 2.2 | 8.9 | 264 | 6.1 | 3.4 | 10.9 | 287 | 3.7 |
| Nigeria | 9.1 | 6.7 | 12.1 | 611 | 6.3 | 4.5 | 8.9 | 638 | 5.1 | 3.4 | 7.5 | 525 | 12.0 | 8.5 | 16.6 | 551 | 10.9 | 8.0 | 14.8 | 423 | 8.5 |
| Pakistan | 19.6 | 14.2 | 26.4 | 256 | 23.3 | 16.6 | 31.7 | 220 | 22.1 | 15.5 | 30.5 | 204 | 30.3 | 22.6 | 39.3 | 210 | 26.3 | 17.9 | 36.9 | 227 | 24.2 |
| Panama | 12.5 | 7.3 | 20.6 | 285 | 3.9 | 1.2 | 11.6 | 86 | 11.5 | 4.1 | 28.3 | 67 | 1.4 | 0.2 | 9.5 | 42 | 1.6 | 0.4 | 5.3 | 30 | 6.7 |
| Paraguay | 17.8 | 10.0 | 29.6 | 105 | 8.6 | 3.9 | 17.7 | 92 | 9.9 | 2.6 | 30.9 | 80 | 2.9 | 0.8 | 9.3 | 68 | 1.8 | 0.2 | 12.2 | 49 | 9.5 |
| Peru | 4.5 | 2.7 | 7.3 | 564 | 3.1 | 1.7 | 5.5 | 444 | 8.1 | 5.1 | 12.8 | 326 | 3.6 | 1.6 | 7.8 | 244 | 9.1 | 4.7 | 17.0 | 159 | 5.4 |
| Rwanda | 2.6 | 0.8 | 8.0 | 152 | 2.1 | 0.7 | 6.4 | 141 | 4.8 | 2.2 | 10.2 | 124 | 2.1 | 0.7 | 6.3 | 131 | 5.8 | 3.1 | 10.8 | 155 | 3.5 |
| Senegal | 5.4 | 2.8 | 10.1 | 350 | 2.7 | 1.2 | 5.9 | 306 | 2.0 | 0.7 | 5.5 | 244 | 0.4 | 0.1 | 2.9 | 151 | 3.1 | 0.8 | 11.7 | 91 | 2.9 |
| Serbia | 24.3 | 6.1 | 61.4 | 17 | 10.4 | 3.4 | 27.9 | 23 | 5.5 | 0.7 | 31.3 | 40 | 2.4 | 0.8 | 7.3 | 39 | 2.4 | 0.5 | 11.4 | 50 | 5.7 |
| Sierra Leone | 1.6 | 0.7 | 3.7 | 312 | 3.0 | 1.3 | 6.7 | 287 | 4.4 | 2.4 | 7.9 | 287 | 5.7 | 2.7 | 11.6 | 166 | 14.5 | 8.9 | 22.7 | 118 | 5.3 |
| South Africa | 8.8 | 3.0 | 23.1 | 68 | 10.3 | 3.8 | 25.3 | 86 | 7.4 | 3.0 | 17.0 | 89 | 8.7 | 3.8 | 18.7 | 60 | 19.3 | 8.5 | 38.1 | 43 | 10.6 |
| South Sudan | 8.5 | 5.0 | 13.9 | 156 | 17.1 | 11.8 | 24.2 | 164 | 9.7 | 5.8 | 15.7 | 166 | 10.9 | 6.6 | 17.4 | 195 | 6.4 | 3.6 | 11.3 | 196 | 10.4 |
| State of Palestine | 0.0 | - | - | 154 | 1.6 | 0.4 | 6.4 | 128 | 2.3 | 0.7 | 7.2 | 121 | 2.2 | 0.8 | 5.6 | 154 | 5.8 | 2.7 | 12.3 | 108 | 2.1 |
| Sudan | 10.9 | 7.2 | 16.1 | 367 | 11.7 | 6.8 | 19.3 | 435 | 7.2 | 4.5 | 11.3 | 352 | 9.3 | 5.5 | 15.4 | 223 | 5.8 | 2.8 | 11.5 | 166 | 9.3 |
| Suriname | 18.8 | 11.2 | 29.9 | 112 | 4.1 | 1.3 | 12.1 | 79 | 21.2 | 9.0 | 42.3 | 60 | 12.5 | 3.7 | 34.9 | 50 | 13.3 | 3.9 | 36.8 | 34 | 14.3 |
| Tajikistan | 8.3 | 4.3 | 15.4 | 99 | 10.5 | 5.0 | 20.8 | 79 | 13.5 | 7.3 | 23.6 | 112 | 10.3 | 5.8 | 17.5 | 121 | 9.3 | 4.8 | 17.1 | 142 | 10.5 |
| Tanzania | 7.4 | 4.7 | 11.4 | 258 | 4.8 | 2.3 | 9.6 | 209 | 4.8 | 2.6 | 8.5 | 179 | 4.9 | 2.5 | 9.6 | 214 | 8.4 | 4.7 | 14.4 | 155 | 6.0 |
| Thailand | 11.4 | 3.1 | 34.0 | 151 | 9.6 | 3.6 | 23.3 | 153 | 9.2 | 3.6 | 21.8 | 136 | 25.2 | 13.0 | 43.2 | 130 | 1.5 | 0.4 | 5.4 | 91 | 12.6 |
| Timor Leste | 1.0 | 0.1 | 6.9 | 126 | 3.1 | 0.9 | 10.0 | 150 | 6.1 | 2.9 | 12.3 | 158 | 6.2 | 2.6 | 14.3 | 160 | 21.4 | 13.7 | 31.9 | 149 | 8.0 |
| Togo | 0.4 | 0.1 | 2.7 | 199 | 0.5 | 0.1 | 3.2 | 114 | 0.0 | - | - | 102 | 1.2 | 0.2 | 7.9 | 97 | 2.4 | 0.8 | 7.2 | 91 | 0.9 |
| Tunisia | 7.5 | 2.7 | 18.9 | 56 | 0.8 | 0.1 | 5.5 | 62 | 8.9 | 3.8 | 19.2 | 65 | 6.9 | 2.6 | 17.2 | 70 | 2.6 | 0.4 | 16.8 | 46 | 5.6 |
| Turkmenistan | 0.0 | - | - | 49 | 2.1 | 0.3 | 13.4 | 59 | 4.5 | 1.1 | 16.2 | 70 | 0.0 | - | - | 84 | 1.5 | 0.2 | 10.2 | 80 | 1.6 |
| Uganda | 4.8 | 2.7 | 8.5 | 400 | 7.3 | 4.8 | 11.0 | 331 | 9.3 | 5.8 | 14.5 | 286 | 17.6 | 12.7 | 23.8 | 255 | 19.2 | 14.2 | 25.5 | 210 | 11.0 |
| Ukraine | 18.1 | 9.4 | 31.9 | 65 | 14.8 | 6.0 | 32.1 | 76 | 11.8 | 3.5 | 32.9 | 49 | 6.4 | 2.4 | 16.1 | 57 | 10.3 | 2.2 | 37.0 | 60 | 11.9 |
| Vietnam | 9.5 | 3.7 | 22.0 | 89 | 21.0 | 11.4 | 35.5 | 68 | 23.9 | 14.3 | 37.1 | 74 | 11.1 | 5.5 | 21.2 | 68 | 13.8 | 6.6 | 26.7 | 59 | 16.2 |
| Zambia | 0.0 | - | - | 288 | 1.2 | 0.4 | 3.3 | 309 | 0.3 | 0.1 | 1.1 | 251 | 0.6 | 0.1 | 2.6 | 203 | 3.7 | 1.4 | 9.1 | 138 | 0.9 |
| Zimbabwe | 0.0 | - | - | 120 | 0.0 | - | - | 117 | 0.0 | - | - | 99 | 1.7 | 0.5 | 5.6 | 161 | 3.6 | 1.0 | 12.7 | 106 | 0.9 |

^*^DHS: Demographic Health Survey; MICS: Multiple Indicator Cluster Survey; ENSANUT: Encuesta Nacional de Salud y Nutrición; ENDES: Encuesta Demográfica y de Salud Familiar; ^†^CAR: Central African Republic; ^‡^CDR: Congo Democratic Republic.

**Supplementary table 8. Percentage of children between 6-23 months who were fed other non-human milk (other than formula) at the time of the survey by wealth quintiles. Source: DHS, MICS, ENSANUT, and ENDES, 2010-2018.^a^**

| **Country** | **Other non-human milk consumption between 6-23 months** | | | | | | | | | | | | | | | | | | | | |
| --- | --- | --- | --- | --- | --- | --- | --- | --- | --- | --- | --- | --- | --- | --- | --- | --- | --- | --- | --- | --- | --- |
|  | **Wealth quintiles** | | | | | | | | | | | | | | | | | | | | **National prevalence (%)** |
|  | **Poorest** | | | | **Second** | | | | **Third** | | | | **Fourth** | | | | **Wealthiest** | | | |  |
|  | **%** | **95% CI** | | **N** | **%** | **95% CI** | | **N** | **%** | **95% CI** | | **N** | **%** | **95% CI** | | **N** | **%** | **95% CI** | | **N** |  |
| Afghanistan | 41.5 | 37.3 | 45.9 | 1408 | 41.5 | 37.3 | 45.8 | 1801 | 32.6 | 27.9 | 37.6 | 1796 | 31.6 | 27.6 | 35.9 | 1829 | 40.4 | 33.6 | 47.5 | 1242 | 37.2 |
| Albania | 51.0 | 41.7 | 60.2 | 243 | 42.0 | 32.2 | 52.5 | 184 | 44.0 | 32.7 | 55.9 | 144 | 41.9 | 29.2 | 55.7 | 121 | 26.2 | 15.3 | 40.9 | 74 | 41.6 |
| Algeria | 75.8 | 72.2 | 79.1 | 1018 | 78.2 | 74.5 | 81.6 | 937 | 75.8 | 71.9 | 79.2 | 907 | 73.1 | 69.2 | 76.7 | 865 | 77.0 | 72.2 | 81.3 | 671 | 76.0 |
| Angola | 2.9 | 1.9 | 4.6 | 943 | 2.8 | 1.9 | 4.2 | 1159 | 11.2 | 8.6 | 14.5 | 1000 | 16.2 | 12.3 | 21.0 | 532 | 31.6 | 24.7 | 39.5 | 374 | 11.4 |
| Argentina | 59.1 | 53.1 | 64.9 | 672 | 60.8 | 53.6 | 67.5 | 523 | 57.7 | 50.3 | 64.8 | 512 | 54.3 | 46.5 | 61.8 | 476 | 46.6 | 38.0 | 55.4 | 455 | 56.4 |
| Armenia | 31.5 | 22.4 | 42.2 | 95 | 39.8 | 30.4 | 50.1 | 112 | 33.0 | 24.3 | 43.1 | 107 | 29.4 | 19.7 | 41.4 | 96 | 36.6 | 27.9 | 46.2 | 89 | 34.4 |
| Bangladesh | 19.7 | 15.6 | 24.7 | 493 | 25.8 | 20.1 | 32.4 | 434 | 34.1 | 27.3 | 41.7 | 467 | 37.5 | 32.0 | 43.4 | 496 | 32.5 | 27.4 | 38.2 | 446 | 29.8 |
| Belarus | 63.2 | 52.8 | 72.4 | 147 | 49.1 | 38.4 | 59.8 | 199 | 47.3 | 38.1 | 56.7 | 206 | 50.4 | 41.7 | 59.0 | 243 | 39.9 | 33.4 | 46.7 | 320 | 47.4 |
| Belize | 35.7 | 27.6 | 44.8 | 185 | 47.5 | 38.9 | 56.3 | 169 | 49.3 | 38.0 | 60.7 | 136 | 41.8 | 31.6 | 52.7 | 140 | 55.3 | 42.0 | 67.8 | 100 | 45.1 |
| Benin | 23.3 | 18.8 | 28.6 | 829 | 13.4 | 10.6 | 16.7 | 778 | 11.5 | 8.6 | 15.4 | 775 | 8.5 | 6.5 | 11.0 | 770 | 11.8 | 9.1 | 15.1 | 730 | 13.8 |
| Bhutan | 24.6 | 19.3 | 30.8 | 408 | 26.2 | 21.5 | 31.5 | 439 | 20.3 | 16.0 | 25.5 | 394 | 18.4 | 13.4 | 24.8 | 382 | 26.7 | 21.1 | 33.2 | 305 | 23.1 |
| Burkina Faso | 14.2 | 11.2 | 17.7 | 775 | 9.4 | 7.3 | 12.1 | 843 | 6.1 | 4.3 | 8.6 | 923 | 5.0 | 3.4 | 7.1 | 906 | 12.5 | 9.7 | 16.0 | 701 | 9.2 |
| Burundi | 0.8 | 0.3 | 2.1 | 797 | 1.1 | 0.5 | 2.2 | 807 | 2.9 | 1.9 | 4.4 | 785 | 4.5 | 3.1 | 6.4 | 704 | 22.4 | 19.1 | 25.9 | 765 | 5.4 |
| CAR^†^ | 0.9 | 0.3 | 2.4 | 766 | 1.1 | 0.6 | 2.0 | 818 | 3.6 | 2.3 | 5.7 | 765 | 9.7 | 6.8 | 13.8 | 580 | 24.0 | 19.4 | 29.4 | 337 | 6.8 |
| Cambodia | 10.4 | 7.3 | 14.7 | 501 | 15.3 | 11.7 | 19.8 | 404 | 19.8 | 15.3 | 25.2 | 320 | 30.9 | 25.6 | 36.8 | 371 | 36.7 | 31.6 | 42.1 | 531 | 22.0 |
| Cameroon | 11.7 | 7.2 | 18.6 | 368 | 13.8 | 9.0 | 20.8 | 489 | 17.0 | 12.6 | 22.6 | 482 | 27.8 | 22.5 | 33.8 | 432 | 41.5 | 35.3 | 47.9 | 357 | 20.8 |
| Chad | 14.3 | 11.4 | 17.7 | 867 | 15.9 | 13.0 | 19.3 | 938 | 22.4 | 18.8 | 26.5 | 940 | 28.0 | 23.6 | 32.8 | 916 | 26.9 | 23.5 | 30.5 | 742 | 21.0 |
| Colombia | 48.0 | 44.8 | 51.2 | 1840 | 45.7 | 42.2 | 49.3 | 1339 | 46.4 | 42.3 | 50.5 | 920 | 43.2 | 38.6 | 47.9 | 572 | 37.9 | 31.9 | 44.2 | 352 | 45.0 |
| Comoros | 11.1 | 6.9 | 17.3 | 218 | 16.0 | 9.8 | 25.1 | 176 | 17.2 | 11.2 | 25.4 | 187 | 17.9 | 12.1 | 25.6 | 154 | 26.9 | 19.3 | 36.1 | 134 | 17.3 |
| Congo Brazzaville | 14.6 | 12.0 | 17.8 | 1220 | 41.8 | 36.2 | 47.7 | 729 | 50.1 | 43.3 | 56.8 | 374 | 58.6 | 50.8 | 66.0 | 243 | 69.4 | 60.6 | 76.9 | 199 | 44.7 |
| CDR^‡^ | 0.3 | 0.1 | 0.9 | 1324 | 2.2 | 0.8 | 5.8 | 1123 | 2.0 | 1.2 | 3.3 | 1031 | 8.3 | 6.0 | 11.4 | 872 | 25.2 | 20.9 | 30.1 | 641 | 6.7 |
| Costa Rica | 44.8 | 31.9 | 58.4 | 222 | 47.9 | 35.1 | 61.0 | 180 | 47.0 | 32.0 | 62.6 | 124 | 45.2 | 29.8 | 61.5 | 99 | 49.1 | 29.0 | 69.5 | 63 | 46.6 |
| Cote d’Ivoire | 7.7 | 5.6 | 10.6 | 783 | 7.2 | 5.2 | 10.0 | 681 | 14.4 | 10.7 | 18.9 | 565 | 19.5 | 15.1 | 24.9 | 384 | 38.9 | 32.7 | 45.4 | 255 | 15.8 |
| Dominican Republic | 67.7 | 64.5 | 70.8 | 2055 | 80.0 | 76.6 | 83.0 | 1383 | 82.6 | 79.0 | 85.7 | 1170 | 84.5 | 80.4 | 87.9 | 895 | 89.7 | 86.1 | 92.4 | 732 | 79.5 |
| Ecuador | 25.1 | 20.7 | 30.1 | 964 | 27.5 | 21.9 | 33.8 | 787 | 31.8 | 25.5 | 38.8 | 616 | 30.2 | 24.4 | 36.7 | 485 | 31.2 | 24.0 | 39.6 | 341 | 28.9 |
| Egypt | 26.7 | 22.3 | 31.6 | 824 | 17.6 | 14.7 | 20.9 | 897 | 25.2 | 22.1 | 28.4 | 985 | 23.6 | 20.5 | 26.9 | 1056 | 27.4 | 23.9 | 31.3 | 1072 | 24.0 |
| El Salvador | 31.2 | 26.9 | 35.8 | 535 | 39.0 | 33.5 | 44.8 | 481 | 45.2 | 39.1 | 51.4 | 456 | 45.7 | 40.0 | 51.5 | 434 | 45.3 | 37.6 | 53.2 | 360 | 40.6 |
| Eswatini | 12.7 | 8.1 | 19.4 | 216 | 21.5 | 15.3 | 29.3 | 194 | 19.6 | 13.9 | 26.9 | 174 | 30.9 | 22.3 | 41.0 | 105 | 41.3 | 29.3 | 54.4 | 100 | 24.0 |
| Ethiopia | 16.7 | 13.3 | 20.7 | 950 | 11.0 | 7.9 | 15.1 | 474 | 13.3 | 10.0 | 17.6 | 423 | 13.9 | 10.2 | 18.7 | 347 | 34.2 | 26.2 | 43.1 | 627 | 16.9 |
| Gabon | 24.0 | 20.1 | 28.4 | 806 | 38.1 | 30.5 | 46.3 | 373 | 46.7 | 39.0 | 54.5 | 244 | 42.7 | 30.9 | 55.5 | 175 | 37.8 | 26.8 | 50.2 | 121 | 37.9 |
| Gambia | 7.7 | 5.3 | 11.1 | 605 | 9.4 | 6.6 | 13.1 | 653 | 7.9 | 4.8 | 12.6 | 532 | 14.3 | 9.5 | 21.0 | 352 | 18.0 | 12.5 | 25.3 | 281 | 11.1 |
| Ghana | 4.5 | 2.7 | 7.5 | 542 | 8.4 | 4.8 | 14.0 | 369 | 11.2 | 7.4 | 16.6 | 287 | 21.9 | 16.3 | 28.7 | 256 | 31.1 | 23.7 | 39.5 | 202 | 14.7 |
| Guatemala | 5.8 | 4.1 | 8.1 | 943 | 11.2 | 8.8 | 14.0 | 804 | 19.8 | 16.6 | 23.5 | 720 | 31.7 | 27.2 | 36.6 | 610 | 40.1 | 34.0 | 46.6 | 432 | 19.2 |
| Guinea | 9.0 | 6.3 | 12.6 | 469 | 9.1 | 6.4 | 12.8 | 469 | 15.8 | 11.4 | 21.5 | 450 | 17.0 | 13.6 | 21.0 | 417 | 22.0 | 16.7 | 28.4 | 317 | 14.3 |
| Guinea Bissau | 5.5 | 3.8 | 8.1 | 688 | 8.2 | 5.0 | 13.1 | 530 | 8.1 | 5.6 | 11.6 | 524 | 13.1 | 9.0 | 18.6 | 348 | 38.9 | 30.6 | 47.9 | 178 | 12.8 |
| Guyana | 66.6 | 59.7 | 72.8 | 373 | 70.9 | 60.9 | 79.2 | 201 | 75.1 | 66.5 | 82.2 | 163 | 82.0 | 72.3 | 88.8 | 150 | 79.2 | 69.0 | 86.6 | 147 | 73.4 |
| Haiti | 29.9 | 25.1 | 35.1 | 543 | 21.5 | 17.0 | 26.8 | 380 | 28.4 | 23.1 | 34.4 | 321 | 36.7 | 29.8 | 44.1 | 231 | 43.2 | 34.9 | 51.8 | 177 | 30.7 |
| Honduras | 29.1 | 26.1 | 32.2 | 1069 | 45.8 | 41.7 | 49.9 | 763 | 56.1 | 51.7 | 60.3 | 589 | 67.8 | 63.2 | 72.1 | 469 | 68.2 | 62.1 | 73.7 | 347 | 51.4 |
| India | 31.8 | 30.9 | 32.7 | 18220 | 40.4 | 39.3 | 41.5 | 16608 | 46.2 | 44.9 | 47.4 | 14567 | 53.1 | 51.6 | 54.6 | 12234 | 58.9 | 57.3 | 60.4 | 10116 | 44.6 |
| Indonesia | 10.8 | 8.5 | 13.6 | 1324 | 11.5 | 9.1 | 14.6 | 990 | 14.2 | 11.6 | 17.2 | 912 | 11.5 | 9.4 | 14.1 | 927 | 12.3 | 10.0 | 15.2 | 880 | 12.1 |
| Iraq | 22.3 | 18.6 | 26.4 | 1297 | 21.1 | 15.3 | 28.5 | 1109 | 18.7 | 12.7 | 26.5 | 942 | 18.4 | 14.9 | 22.5 | 790 | 21.6 | 13.3 | 33.1 | 648 | 20.5 |
| Jamaica | 33.2 | 24.1 | 43.8 | 129 | 42.2 | 29.5 | 56.0 | 100 | 38.2 | 25.8 | 52.3 | 88 | 31.0 | 20.4 | 44.0 | 85 | 29.0 | 17.3 | 44.2 | 74 | 35.2 |
| Jordan | 41.6 | 37.1 | 46.3 | 991 | 40.2 | 34.8 | 45.7 | 720 | 45.9 | 39.5 | 52.4 | 565 | 45.8 | 38.0 | 53.8 | 293 | 45.2 | 31.9 | 59.3 | 111 | 43.3 |
| Kazakhstan | 56.2 | 47.4 | 64.6 | 306 | 59.7 | 54.0 | 65.1 | 312 | 54.6 | 46.2 | 62.6 | 386 | 41.7 | 33.5 | 50.3 | 338 | 38.3 | 32.1 | 45.0 | 290 | 51.0 |
| Kenya | 46.5 | 42.1 | 51.0 | 990 | 40.9 | 36.2 | 45.8 | 584 | 47.4 | 41.6 | 53.2 | 442 | 53.3 | 46.9 | 59.6 | 428 | 61.7 | 55.2 | 67.8 | 365 | 49.8 |
| Kosovo | 46.7 | 36.2 | 57.4 | 105 | 53.2 | 42.3 | 63.9 | 101 | 50.8 | 41.1 | 60.5 | 103 | 48.7 | 38.0 | 59.6 | 99 | 56.3 | 44.7 | 67.3 | 82 | 50.9 |
| Kyrgyzstan | 31.5 | 23.7 | 40.4 | 222 | 36.4 | 27.4 | 46.5 | 170 | 40.7 | 32.5 | 49.6 | 190 | 45.6 | 38.2 | 53.1 | 245 | 42.8 | 34.0 | 52.1 | 165 | 38.8 |
| Lao | 8.2 | 6.1 | 11.1 | 913 | 15.2 | 12.3 | 18.5 | 843 | 21.7 | 18.1 | 25.7 | 645 | 33.6 | 29.2 | 38.4 | 549 | 38.8 | 34.0 | 43.9 | 478 | 22.1 |
| Lesotho | 8.7 | 5.6 | 13.3 | 264 | 20.2 | 14.3 | 27.8 | 193 | 16.1 | 10.8 | 23.2 | 203 | 28.2 | 20.7 | 37.1 | 157 | 46.1 | 37.0 | 55.5 | 131 | 22.1 |
| Liberia | 1.9 | 0.7 | 5.2 | 779 | 2.8 | 1.4 | 5.5 | 562 | 5.8 | 2.3 | 13.7 | 430 | 12.1 | 5.6 | 24.1 | 240 | 24.1 | 17.9 | 31.7 | 144 | 8.1 |
| Malawi | 2.6 | 1.6 | 4.3 | 1120 | 3.4 | 2.0 | 5.5 | 1053 | 4.8 | 3.0 | 7.5 | 920 | 5.4 | 3.8 | 7.4 | 852 | 13.9 | 10.8 | 17.8 | 802 | 5.4 |
| Mali | 36.7 | 32.2 | 41.4 | 997 | 27.4 | 23.6 | 31.6 | 976 | 25.5 | 22.0 | 29.4 | 940 | 26.9 | 23.7 | 30.3 | 1036 | 37.3 | 33.8 | 41.0 | 916 | 30.5 |
| Mauritania | 62.5 | 56.8 | 67.8 | 603 | 57.7 | 52.6 | 62.7 | 668 | 60.5 | 55.9 | 64.9 | 711 | 62.6 | 56.3 | 68.5 | 631 | 65.4 | 59.9 | 70.5 | 571 | 61.7 |
| Mexico | 42.4 | 36.2 | 48.9 | 724 | 42.7 | 35.6 | 50.2 | 573 | 44.3 | 35.1 | 54.0 | 453 | 49.4 | 39.5 | 59.4 | 353 | 40.1 | 28.2 | 53.3 | 208 | 43.8 |
| Moldova | 45.8 | 33.8 | 58.4 | 73 | 51.7 | 40.9 | 62.3 | 95 | 46.4 | 35.9 | 57.3 | 94 | 53.3 | 42.4 | 63.9 | 111 | 34.8 | 28.2 | 42.0 | 218 | 45.6 |
| Mongolia | 51.8 | 46.2 | 57.2 | 436 | 46.8 | 40.9 | 52.7 | 332 | 42.2 | 36.7 | 47.9 | 340 | 45.3 | 39.6 | 51.2 | 331 | 53.9 | 49.1 | 58.7 | 363 | 48.3 |
| Myanmar | 9.8 | 6.3 | 14.8 | 376 | 11.5 | 7.5 | 17.3 | 299 | 7.9 | 4.5 | 13.5 | 253 | 11.9 | 7.9 | 17.6 | 241 | 23.0 | 16.8 | 30.6 | 170 | 12.3 |
| Namibia | 9.0 | 5.5 | 14.6 | 280 | 8.8 | 5.7 | 13.4 | 306 | 12.9 | 9.2 | 17.8 | 295 | 25.5 | 18.4 | 34.2 | 253 | 41.0 | 31.1 | 51.6 | 168 | 17.3 |
| Nepal | 46.4 | 40.4 | 52.6 | 355 | 39.8 | 33.4 | 46.6 | 321 | 42.9 | 36.8 | 49.2 | 316 | 52.2 | 44.9 | 59.3 | 292 | 67.6 | 57.8 | 76.0 | 179 | 48.3 |
| Niger | 10.5 | 7.7 | 14.2 | 520 | 8.8 | 6.5 | 11.9 | 570 | 7.8 | 5.6 | 10.9 | 603 | 10.5 | 7.9 | 13.7 | 636 | 17.0 | 14.0 | 20.4 | 930 | 10.9 |
| Nigeria | 16.7 | 14.2 | 19.5 | 1789 | 12.8 | 10.8 | 15.0 | 1822 | 16.7 | 14.1 | 19.7 | 1578 | 27.6 | 24.0 | 31.6 | 1519 | 45.0 | 41.7 | 48.4 | 1511 | 23.1 |
| Pakistan | 32.0 | 25.6 | 39.1 | 562 | 44.5 | 37.9 | 51.4 | 510 | 50.6 | 44.4 | 56.8 | 510 | 56.8 | 50.4 | 62.9 | 468 | 62.9 | 56.7 | 68.8 | 515 | 49.1 |
| Panama | 41.4 | 35.7 | 47.2 | 998 | 44.5 | 36.1 | 53.3 | 359 | 47.2 | 35.9 | 58.8 | 250 | 35.9 | 26.9 | 46.0 | 221 | 44.9 | 30.8 | 59.9 | 97 | 42.5 |
| Paraguay | 50.3 | 42.9 | 57.8 | 412 | 59.3 | 52.0 | 66.3 | 319 | 58.8 | 50.4 | 66.7 | 272 | 68.9 | 61.5 | 75.5 | 262 | 56.6 | 47.1 | 65.7 | 178 | 58.5 |
| Peru | 31.1 | 28.4 | 34.0 | 1908 | 40.1 | 37.0 | 43.2 | 1699 | 47.7 | 44.2 | 51.1 | 1282 | 43.8 | 39.7 | 48.0 | 939 | 39.5 | 34.8 | 44.4 | 706 | 39.9 |
| Rwanda | 11.5 | 9.0 | 14.6 | 593 | 14.8 | 11.7 | 18.6 | 475 | 17.2 | 13.8 | 21.1 | 433 | 31.5 | 26.8 | 36.6 | 402 | 44.3 | 38.6 | 50.1 | 450 | 22.4 |
| Senegal | 22.8 | 19.4 | 26.7 | 1072 | 22.1 | 18.4 | 26.3 | 871 | 24.8 | 20.3 | 29.9 | 768 | 33.0 | 26.9 | 39.8 | 469 | 32.6 | 26.3 | 39.5 | 309 | 26.4 |
| Serbia | 57.9 | 35.7 | 77.2 | 123 | 72.5 | 61.2 | 81.6 | 128 | 67.7 | 56.3 | 77.3 | 164 | 68.1 | 59.4 | 75.7 | 175 | 56.3 | 44.7 | 67.3 | 205 | 64.1 |
| Sierra Leone | 4.0 | 2.9 | 5.4 | 1028 | 4.6 | 3.3 | 6.4 | 836 | 8.4 | 6.1 | 11.5 | 732 | 18.2 | 14.5 | 22.5 | 460 | 36.7 | 31.8 | 41.8 | 355 | 12.5 |
| South Africa | 15.6 | 10.3 | 22.8 | 237 | 26.0 | 18.4 | 35.3 | 213 | 31.0 | 23.6 | 39.6 | 180 | 52.1 | 43.2 | 60.8 | 164 | 39.3 | 28.2 | 51.6 | 83 | 31.4 |
| South Sudan | 25.2 | 21.4 | 29.5 | 482 | 22.2 | 18.0 | 27.0 | 490 | 23.3 | 19.4 | 27.7 | 491 | 21.9 | 17.6 | 26.9 | 560 | 26.4 | 22.7 | 30.5 | 530 | 23.8 |
| State of Palestine | 36.6 | 32.2 | 41.2 | 557 | 35.5 | 31.1 | 40.1 | 422 | 32.6 | 28.5 | 37.0 | 500 | 39.9 | 35.3 | 44.6 | 486 | 39.0 | 33.9 | 44.3 | 361 | 36.6 |
| Sudan | 47.8 | 42.5 | 53.1 | 854 | 45.2 | 40.8 | 49.6 | 1087 | 55.4 | 51.2 | 59.6 | 914 | 57.7 | 48.6 | 66.3 | 689 | 68.9 | 64.2 | 73.3 | 520 | 54.3 |
| Suriname | 13.0 | 9.4 | 17.7 | 326 | 22.4 | 15.8 | 30.7 | 273 | 21.5 | 15.4 | 29.2 | 229 | 22.0 | 15.7 | 29.9 | 212 | 15.3 | 10.0 | 22.9 | 142 | 18.7 |
| Tajikistan | 43.4 | 36.6 | 50.5 | 295 | 39.6 | 33.3 | 46.2 | 309 | 45.1 | 39.7 | 50.7 | 351 | 34.2 | 28.6 | 40.2 | 356 | 40.1 | 34.5 | 45.9 | 411 | 40.5 |
| Tanzania | 22.9 | 19.0 | 27.3 | 682 | 12.9 | 10.1 | 16.4 | 609 | 11.6 | 8.7 | 15.3 | 572 | 14.8 | 11.8 | 18.4 | 645 | 26.1 | 21.6 | 31.2 | 511 | 17.7 |
| Thailand | 59.3 | 50.8 | 67.3 | 690 | 61.5 | 53.9 | 68.6 | 725 | 60.3 | 51.8 | 68.2 | 688 | 56.4 | 46.1 | 66.1 | 702 | 51.2 | 39.0 | 63.2 | 417 | 58.3 |
| Timor Leste | 3.2 | 1.8 | 5.7 | 381 | 6.2 | 4.0 | 9.5 | 413 | 8.6 | 5.8 | 12.7 | 414 | 14.4 | 10.4 | 19.5 | 439 | 27.3 | 21.0 | 34.7 | 303 | 11.5 |
| Togo | 5.1 | 3.3 | 8.0 | 590 | 1.4 | 0.7 | 2.8 | 425 | 1.8 | 0.8 | 3.9 | 419 | 5.3 | 3.0 | 8.9 | 331 | 11.9 | 8.6 | 16.4 | 305 | 5.0 |
| Tunisia | 54.0 | 45.8 | 62.0 | 199 | 68.8 | 62.2 | 74.7 | 224 | 67.6 | 60.1 | 74.4 | 184 | 69.7 | 62.4 | 76.1 | 204 | 65.0 | 56.2 | 72.9 | 135 | 65.3 |
| Turkmenistan | 51.8 | 44.2 | 59.2 | 198 | 44.7 | 37.6 | 52.0 | 215 | 46.4 | 38.9 | 54.0 | 251 | 42.2 | 35.4 | 49.2 | 258 | 51.6 | 44.8 | 58.4 | 247 | 47.3 |
| Uganda | 13.1 | 10.9 | 15.7 | 1126 | 22.1 | 18.6 | 26.0 | 913 | 23.3 | 20.0 | 27.1 | 781 | 35.5 | 31.0 | 40.2 | 673 | 48.8 | 43.6 | 54.0 | 665 | 28.0 |
| Ukraine | 76.9 | 69.2 | 83.1 | 268 | 71.0 | 62.0 | 78.6 | 301 | 72.6 | 61.9 | 81.3 | 197 | 71.1 | 60.4 | 79.8 | 240 | 52.3 | 42.9 | 61.6 | 250 | 68.4 |
| Vietnam | 36.7 | 29.2 | 44.8 | 264 | 61.9 | 54.0 | 69.1 | 200 | 61.4 | 53.6 | 68.6 | 194 | 65.4 | 57.5 | 72.6 | 231 | 54.0 | 46.7 | 61.2 | 229 | 56.1 |
| Zambia | 3.0 | 1.8 | 4.7 | 920 | 2.9 | 1.8 | 4.6 | 870 | 4.9 | 3.3 | 7.1 | 847 | 4.9 | 3.1 | 7.5 | 617 | 10.4 | 7.5 | 14.2 | 468 | 4.8 |
| Zimbabwe | 5.9 | 3.7 | 9.3 | 369 | 5.4 | 3.5 | 8.2 | 301 | 4.3 | 2.3 | 7.9 | 268 | 5.5 | 3.3 | 9.0 | 395 | 14.4 | 10.0 | 20.2 | 295 | 6.6 |

^*^DHS: Demographic Health Survey; MICS: Multiple Indicator Cluster Survey; ENSANUT: Encuesta Nacional de Salud y Nutrición; ENDES: Encuesta Demográfica y de Salud Familiar; ^†^CAR: Central African Republic; ^‡^CDR: Congo Democratic Republic.
